# Changes in admissions, care processes and outcomes for very and extremely preterm infants in England and Wales: an 11-year whole population study

**DOI:** 10.1136/bmjph-2025-004256

**Published:** 2026-03-31

**Authors:** Mohammad Chehrazi, Kayleigh Ougham, Chris Gale, Cheryl Battersby, Sabita Uthaya, Neena Modi

**Affiliations:** 1 Section of Neonatal Medicine, School of Public Health, Imperial College London, London, UK; 2 Centre for Paediatrics and Child Health, Imperial College London, London, England, UK

**Keywords:** Community Health, Comorbidity, Public Health, statistics and numerical data, trends

## Abstract

**Introduction:**

Preterm birth is a major public health concern with lifelong consequences. National policies in England and Wales aim to reduce deaths, improve outcomes and address disparities. We examined changes in admissions, care processes and outcomes among extremely (EPT) and very preterm (VPT) infants overall and by maternal ethnicity.

**Methods:**

We conducted a retrospective, population-based cohort study using the National Neonatal Research Database and Office for National Statistics data, covering all National Health Service neonatal units from 2013 to 2023. Temporal trends in care processes and clinical outcomes were estimated using modified Poisson regression. Models were adjusted for gestational age, sex, multiplicity and birth weight z-score. Results are presented as adjusted risk ratios with 95% CIs.

**Results:**

There were 26 132 EPT and 55 789 VPT admissions, with no significant change in overall admission rates. Admissions of babies born below 24 weeks gestation almost doubled. Mothers of black ethnicity consistently had the highest rates of EPT and VPT admissions. Deliveries by emergency caesarean section increased (EPT: 1.04, 1.03 to 1.04; VPT: 1.02, 1.01 to 1.02). Delivery room intubation (EPT: 0.96, 0.96 to 0.97; VPT: 0.93, 0.93 to 0.93), intubated respiratory support (EPT: 0.99, 0.99 to 0.99; VPT: 0.96, 0.96 to 0.97), and surgical or device closure of patent ductus arteriosus closure (EPT: 0.82, 0.80 to 0.84; VPT: 0.86, 0.81 to 0.92) decreased. In EPT infants, maternal milk uses at discharge (1.01, 1.01 to 1.02) and late-onset bloodstream infection increased (1.03, 1.02 to 1.04) and early postnatal transfers (0.98, 0.97 to 0.99) and mortality decreased (0.98, 0.97 to 0.99). In VPT infants, severe necrotising enterocolitis (0.96, 0.94 to 0.98) and survival without major morbidity decreased (0.99, 0.99 to 0.99).

**Conclusions:**

Over the last decade, EPT and VPT admission rates in England and Wales have not changed and ethnic disparities remain evident. Delivery of some evidence-based care processes improved, but clinical outcomes showed only modest gains.

**WHAT IS ALREADY KNOWN ON THIS TOPIC**

- Preterm birth is a major public health and societal issue necessitating highly specialised care, with survivors at substantial risk of life-long physical and mental health problems.
- Reducing preterm births are national policy targets but globally rates have remained static for the last decade.

**WHAT THIS STUDY ADDS**

- Over the 11 years to December 2023, national rates of extremely and very preterm admissions to neonatal units in England and Wales remained largely unchanged and continue highest for mothers of black ethnicity.
- Delivery of some evidence-based care processes increased, and extremely preterm mortality decreased, but there has been little substantive reduction in major morbidities or improvement in survival without major morbidity.

**HOW THIS STUDY MIGHT AFFECT RESEARCH, PRACTICE OR POLICY**

- Our study indicates that a focus for future research should be identification of socioeconomic and environmental determinants of preterm birth as public health policies may prove to be more effective than healthcare interventions.

## Introduction

 Preterm birth is a major public health and societal issue and the largest contributor globally to loss of disability adjusted life years.^1^ Worldwide, the complications of preterm birth are the leading cause of mortality in children under the age of 5 years. On average, around 1 in 10 of all births are preterm (less than 37 weeks gestation). Worldwide, approximately 15% of all preterm births occur at less than 32 weeks gestation, of which around one-third are <28 weeks (extremely preterm; EPT) and around two-thirds are 28–31^+6^ (very preterm; VPT).^2^

Babies born EPT and VPT require care that is highly specialised and often prolonged. Mortality and morbidity can be high, especially in the most immature infants, born below 24 weeks gestation, often referred to as the margins of viability. In England and Wales, neonatal care is delivered through National Health Service (NHS) Operational Delivery Networks. Each network includes a variable number of neonatal units; designated a special care unit, local neonatal unit or neonatal intensive care unit (NICU). These respectively provide increasing intensity of care. Depending on care needs, a baby may undergo transfer between neonatal units usually within a regional network. NHS hospitals deliver care for all EPT and most VPT infants.

Neonatal care influences life-long health; hence, survival and morbidities to neonatal unit discharge are important intermediary outcome measures, widely considered indicative of quality of care. Reducing preterm birth and its consequences, and ethnic disparities in care and outcomes are major national goals.^3 4^ The UK Government, in its response^5^ to a House of Lords Committee^6^ on preterm birth, has stated that it remains committed to reducing preterm births and improving outcomes for those born preterm. National initiatives include the establishment of maternal medicine networks that provide specialist care for women with high-risk medical conditions, such as diabetes, hypertension, epilepsy and heart and renal disease. More than £20 million has been allocated to local equity and equality action plans for tailored interventions targeting women and babies from minority ethnic backgrounds and those living in the most deprived areas. A third version of the ‘Saving Babies’ Lives Care Bundle’ has been implemented nationally.^7^ This provides guidance for maternity care providers to help reduce preterm births and optimise care where this cannot be avoided. Recommendations include reducing smoking in pregnancy, recognition and management of fetal growth restriction and reduced fetal movements, fetal monitoring during labour, optimising perinatal care and management of diabetes in pregnancy. We therefore aimed to investigate changes in EPT and VPT admissions, and key care processes and outcomes, overall and by maternal ethnicity over an 11-year period in England and Wales.

## Methods

### Data

We used data from the National Neonatal Research Database (NNRD). This is a National Information Asset containing a standard data extract (the Neonatal Data Set, an NHS Information Standard; DAPB1595) from the electronic patient records of all admissions to NHS neonatal units.^8^ The NNRD holds detailed, de-identified demographic (eg, gestational age), daily (eg, daily respiratory support), episodic (eg, transfer within 48 hours), diagnostic (eg, results of ophthalmic examinations) and outcome (eg, mortality) data. Episodes of transfer of an infant between neonatal units are linked together so that each infant record in the NNRD is a complete representation from admission to final neonatal unit discharge. Data undergo a quality assurance process prior to inclusion in the NNRD. The team managing the NNRD notifies neonatal units of potentially erroneous or missing data (eg, those failing out-of-range, internal consistency and logic checks) to enable correction of the infants electronic patient record entry. This is then provided to the NNRD team in the next quarterly extract.

We obtained denominator data from the Office for National Statistics (ONS) for total births, live births and stillbirths,^9^ and from the NNRD for total number of infants admitted to neonatal care by gestational age category and maternal ethnicity. We also obtained ONS denominator data for live births and stillbirths by infant ethnicity. ONS data cover the period 1 January 2013 to 31 December 2023. We evaluated changes in stillbirth rates to determine if this might explain any changes in EPT or VPT admissions. We did not undertake any individual infant level linkage between NNRD and ONS data.

### Population

We included all admissions to neonatal units in England and Wales over the period 1 January 2013 to 31 December 2023 in the analysis. The NNRD contains data from 2007, but we limited the start of this analysis to 1 January 2013 to coincide with the complete coverage of all neonatal units in England and Wales. We grouped admissions by gestational age and maternal ethnicity. Babies were excluded from analyses by maternal ethnicity if that information was missing from the NNRD. We categorised gestational age in accordance with WHO definitions (EPT: <28^+0^ (weeks ^days^); VPT: 28^+0^ to 31^+6^). We categorised infant ethnicity using ONS definitions.^10^ These were Asian (Indian, Pakistani, Bangladeshi, Chinese, other Asian background); black (African, Caribbean, other black background); mixed (white and black Caribbean, white and black African, white and Asian, other mixed background); other (Arab, any other background); white (English/Welsh/Scottish/Northern Irish/British, Irish, Gypsy or Irish traveller, other white background).

### Outcomes

We examined eight key care processes, and seven core clinical outcomes, nationally and by maternal ethnicity. The care processes were any antenatal steroid exposure; birth by emergency caesarean section; birth in a hospital with an NICU (providing tertiary neonatal care); intubation in the delivery room; any intubated respiratory support during hospital stay; receiving any own mother’s milk at discharge; patent ductus arteriosus (PDA) surgical ligation or closure by device, and postnatal transfers in the first 48 hours after birth (upwards, downwards and horizontal). Upward transfer is movement from a lower to higher designation neonatal unit; downward transfer is from a higher to lower designation unit; horizontal transfer is movement between units of the same designation.

The clinical outcomes were mortality; treated retinopathy of prematurity (ROP); severe necrotising enterocolitis (NEC) defined as requiring surgery or resulting in death^10^; bronchopulmonary dysplasia (BPD) defined as receiving any respiratory support or added oxygen at 36 weeks postmenstrual age; severe brain injury using the UK Department of Health definition as relevant to EPT and VPT infants^11^ (intracranial haemorrhage grade 3 or 4, perinatal stroke, central nervous system infection, kernicterus, seizures or white matter injury); late onset bloodstream infection, defined as a pure growth positive blood culture after 72 hours from birth; and survival to discharge without severe morbidity (treated ROP, severe NEC, BPD, severe brain injury).

### Statistical analyses

We report the number of EPT and VPT admissions to neonatal care by maternal ethnicity, both as absolute counts and as rates per 1000 total births (live births and stillbirths). Admission rates by ethnicity were calculated using baby ethnicity as the denominator as this is the only ethnicity variable available in ONS data. For each clinical outcome and care process, we reported proportions relative to the total number of neonatal care admissions by each gestational age category. We also report the number of missing values for each analysis.

Babies born at the margins of viability can disproportionately influence aggregated outcomes due to their higher rates of mortality and morbidity. To account for potential changes over time in the number of such admissions, we conducted a sensitivity analysis excluding babies born before 24+0 weeks’ gestation.

Annual admission rates were analysed using year-specific counts of EPT and VPT admissions fitted with Poisson regression models incorporating a log-offset for the number of live births in each year. The birth year was modelled as a continuous variable so that the estimated coefficient represents the average annual relative change in admission rates. Offset-based rate models were used only for admission counts, as these represent a population-level outcomes rather than an individual-level variable.

Temporal trends in individual-level care processes and clinical outcomes over the study period (2013–2023) were estimated using modified Poisson regression with a log link and robust variance (sandwich) estimation, as described by Zou.^12^ Models were adjusted for sex, gestational age (in weeks), birth weight z-score and multiplicity. We report adjusted risk ratio (aRR) with 95% CIs, with birth year modelled as a continuous variable to estimate the average year-on-year change.

Model adequacy was assessed using the Pearson goodness-of-fit statistic, reported as the Pearson χ^2^ statistic divided by the df. Robust variance estimators were applied for all modified Poisson models to ensure valid inference irrespective of dispersion. To confirm the appropriateness of modelling birth year as a continuous variable, model fit was compared with alternative specifications in which birth year was entered as a categorical variable. As the results and fit statistics were highly similar the continuous specification was retained for parsimony and interpretability. Model-fit statistics for both approaches are provided in the online supplemental table 10.

As a sensitivity analysis for individual-level care process and clinical outcomes, we additionally fitted negative binomial regression models to allow for extra-Poisson variation. These analyses yielded estimates consistent in direction and magnitude with those from the modified Poisson models.

Smoothed trend lines in figures were generated using locally fitted regression means. Ethnicity-specific annual admission rates and pointwise 95% CI were estimated from Poisson regression models with a log-offset for live births. Smoothed trend lines shown in population-level figures are descriptive and are included to aid visual interpretation; formal inference on temporal trends is based on the regression models described above. Missing data were not imputed. As maternal ethnicity and a small number of baseline covariates had missing values, primary analyses were conducted using complete cases. The extent of missing data and a comparison of complete and incomplete cases are reported in online supplemental table 12. To assess the potential for selection bias, we compared baseline characteristics of infants with complete data to those with missing data on any baseline covariate. All data preparation and statistical analysis were conducted using Stata (V.18) (online supplemental Analysis Code).

### Patient and public involvement

A parent/patient advisory group supports the NNRD and the NNRD Steering Board includes parent representatives. Our engagement with parents has shown that they wish to see maximal use of NNRD data and consider ethnic disparities of major concern.

## Results

**Figure 1:**
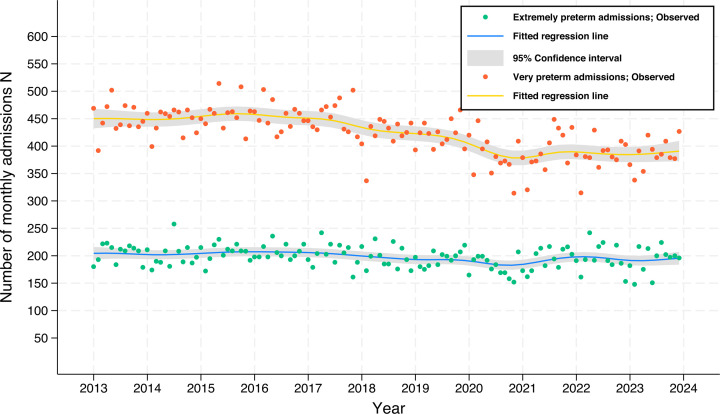
Dots represent the number of extremely preterm and very preterm admissions per month in England and Wales, 2013 to 2022. Solid lines and shading represent the mean and 95% CI.

Admissions, and total, live and stillbirths (figure 1, table 1): There were 1 011 905 infants admitted to neonatal care over the 11-year period 1 January 2013 to 31 December 2023. Of these, 26 132 (2.6%) were EPT and 55 789 (5.5%) were VPT. Nationally, across the 11-year period, there was no statistically significant change in the rate of EPT (aRR 1.01; 95% CI 1.0 to 1.02) and VPT admissions (aRR 0.99; 95% CI 0.99 to 1.0). The admission rate for babies born below 24 weeks’ gestation increased significantly (aRR 1.08; 95% CI 1.07 to 1.09), with the absolute number of admissions almost doubling, from 211 in 2013, to 355 in 2023.

**Table 1:** Total births, live births, stillbirths and admissions by year, England and Wales

	2013	2014	2015	2016	2017	2018	2019	2020	2021	2022	2023	Overall trend
Total births	701 796	698 487	700 999	699 383	681 979	659 765	642 892	616 307	627 425	607 912	592 959	
Live births	698 512	695 233	697 852	696 271	679 106	657 076	640 370	613 936	624 828	605 479	590 637	
Stillbirths N (% of total births); RR (95% CI)	3284 (0.47) Ref	3254 (0.47) 0.99 (0.95 to 1.04)	3147 (0.45) 0.96 (0.91 to 1.01)	3112 (0.45) 0.95 (0.91 to 0.99)	2873 (0.42) 0.90 (0.86 to 0.95)	2689 (0.41) 0.87 (0.83 to 0.92)	2522 (0.39) 0.84 (0.80 to 0.88)	2371 (0.39) 0.82 (0.78 to 0.87)	2597 (0.41) 0.88 (0.84 to 0.93)	2433 (0.40) 0.85 (0.81 to 0.90)	2322 (0.39) 0.84 (0.79 to 0.88)	0.98 (0.97 to 0.98)
EPT admissions (21 w to <28 w gestation) N (% of live births); RR (95% CI)	2458 (0.35) Ref	2407 (0.35) 0.99 (0.90 to 1.07)	2484 (0.36) 1.01 (0.94 to 1.08)	2496 (0.36) 1.02 (0.96 to 1.08)	2418 (0.36) 1.01 (0.93 to 1.10)	2373 (0.36) 1.03 (0.94 to 1.11)	2346 (0.37) 1.04 (0.97 to 1.11)	2164 (0.35) 1.0 (0.92 to 1.08)	2330 (0.37) 1.06 (0.98 to 1.15)	2353 (0.39) 1.10 (1.0 to 1.22)	2810 (0.48) 1.11 (1.01 to 1.21)	1.01 (1.0 to 1.02)
VPT admissions (28 w to <32 w gestation) N (% of live births); RR (95% CI)	5410 (0.77) Ref	5352 (0.77) 0.99 (0.94 to 1.05)	5526 (0.79) 1.02 (0.96 to 1.08)	5452 (0.78) 1.01 (0.96 to 1.06)	5440 (0.80) 1.03 (0.98 to 1.09)	5058 (0.77) 0.99 (0.93 to 1.06)	5053 (0.79) 1.02 (0.96 to 1.07)	4581 (0.75) 0.96 (0.89 to 1.03)	4698 (0.75) 0.97 (0.90 to 1.04)	4570 (0.75) 0.97 (0.92 to 1.04)	4607 (0.78) 1.01 (0.95 to 1.07)	0.99 (0.99 to 1.0)
<24^+0^ w gestation admissions N (% of live births); RR (95% CI)	211 (0.03) Ref	237 (0.03) 1.13 (0.94 to 1.36)	225 (0.03) 1.07 (0.88 to 1.29)	223 (0.03) 1.06 (0.88 to 1.28)	261 (0.04) 1.27 (1.06 to 1.52)	260 (0.04) 1.31 (1.09 to 1.57)	285 (0.04) 1.47 (1.23 to 1.76)	292 (0.05) 1.57 (1.32 to 1.88)	312 (0.05) 1.65 (1.39 to 1.97)	383 (0.06) 2.09 (1.77 to 2.48)	355 (0.06) 1.99 (1.68 to 2.36)	1.08 (1.07 to 1.09)

The ‘Overall trend’ column represents the RR for each 1 year increase in birth year, derived from Poisson regression models incorporating a log-offset for the number of live births in each year.

estimated by modified Poisson regression; the reference year is 2013

EPT, extremely preterm; RR, risk ratio; VPT, very preterm; w, weeks.

The number of total births in England and Wales fell from 701 796 in 2013 to 592 959 in 2023. Statistically significant reductions occurred in the stillbirth rate from 2016 onwards. Figure 1 shows the total number of monthly EPT and VPT admissions. Table 1 summarises annual births, live births, stillbirths and neonatal admissions, including those below 24 weeks gestation.

**Figure 2:**
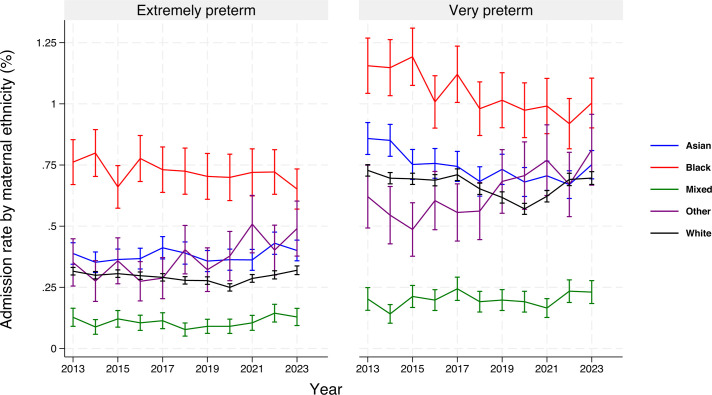
Extremely preterm and very preterm admissions as a proportion of live births by maternal ethnicity and year, 2013–2023. Office for National Statistics data are provided by baby ethnicity as reported by the mother. Ethnicity-specific live births are used as denominators for percentage calculations. Pointwise 95% CIs were calculated for annual admission rates using the corresponding live birth denominators.

Admissions by maternal ethnicity (figure 2, online supplemental tables 1A,B and 2): Throughout the study period, mothers of black ethnicity had the highest rates of EPT and VPT admissions, followed by Asian, white and mixed ethnicity mothers (figure 2). There was a significant decrease over time in the rate of VPT admissions in Asian, black and white mothers; EPT admission rates remained stable (online supplemental table 1A,B). Stillbirth rates were consistently highest among mothers of black ethnicity (online supplemental table 2).

Care processes (online supplemental figure S1, online supplemental table 13,14 and table 3): Between 2013 and 2023, births by emergency caesarean section increased markedly in EPT and VPT admissions (EPT: 37.6% to 50.4%; aRR 1.04, 95% CI 1.03 to 1.04; VPT: 56.4% to 68.0%; 1.02 (1.01 to 1.02). Among EPT admissions, receipt of any own mother’s milk at discharge rose (2013: 49.2%; 2023: 55.1%; 1.01, 1.01 to 1.02).

Across both EPT and VPT infants, delivery room intubation declined on average by 4% and 7% per year, respectively, use of any intubated respiratory support by 1% and 4% per year and PDA closure by surgery or device by 18% and 14% per year.

There were 5063 (19.3%) EPT and 5749 (10.3%) VPT transfers within the first 48 hours of birth over the study period. On average, one in five EPT and one in ten VPT admissions were transferred within the first 48 hours each year (online supplemental figure S1). Among EPT admissions, the proportion of transfers declined significantly, with average annual decreases of 19% for downward transfers, 9% for horizontal transfers and 2% for upward transfers. Among VPT admissions, downward and horizontal transfers decreased on average by 4% and 3% per year, respectively. Sensitivity analyses excluding infants born <24 weeks yielded similar findings (online supplemental table 3).

**Figure 3:**
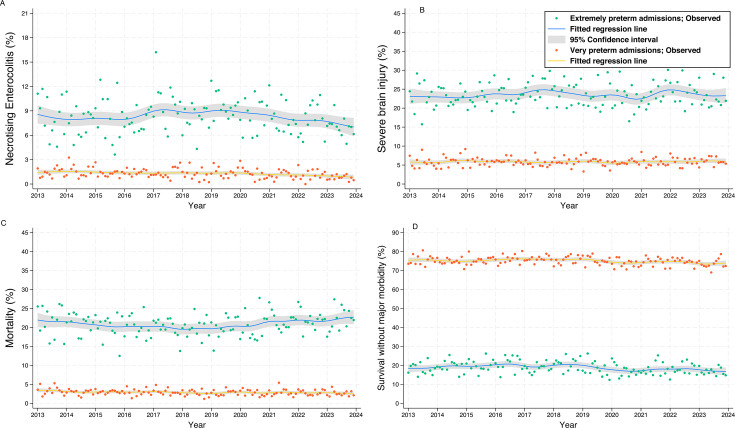
Dots represent the proportion with (**A**) severe necrotising enterocolitis, (**B**) severe brain injury, (**C**) mortality, (**D**) survival without major morbidity, for extremely preterm and very preterm admissions in England and Wales, 2013–2022; solid lines and shading represent the mean and 95% CI.

Clinical outcomes (figures3  4, online supplemental tables 4A and B, table 5): Among EPT infants, mortality decreased on average by 2% per year and late-onset bloodstream infection increased by 3% per year. These findings were unchanged when infants born <24 weeks were excluded (online supplemental table 5).

**Figure 4:**
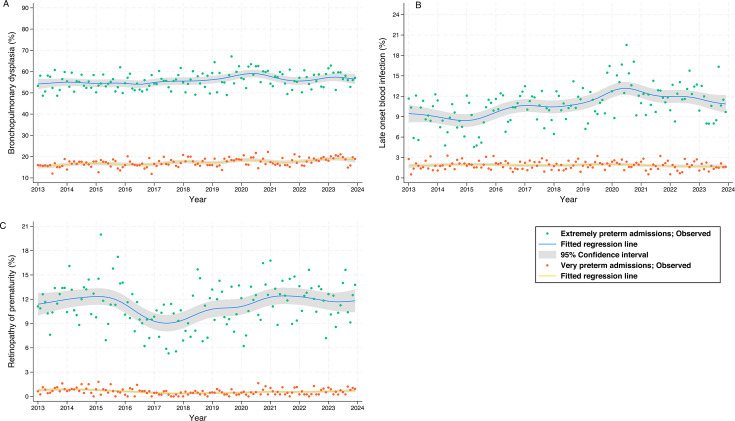
Dots represent the proportion with (**A**) bronchopulmonary dysplasia, (**B**) late onset bloodstream infection and (**C**) treated retinopathy of prematurity for extremely preterm and very preterm admissions in England and Wales, 2013–2022; solid lines and shading represent the mean and 95% CI.

Among VPT infants, severe NEC decreased on average by 4% per year (aRR 0.55 (95% CI 0.37 to 0.81). However, survival to discharge without major morbidity decreased by an average of 1% per year. There were no significant temporal trends in BPD, treated ROP or severe brain injury in either EPT or VPT admissions.

Care processes by maternal ethnicity (online supplemental tables 6A and B, table 7): For both EPT and VPT admissions, deliveries by emergency caesarean section increased across all ethnic groups, with average annual rises of 4% and 2% among Asian infants, 5% and 1% among black infants, 3% and 3% among mixed, 6% and 3% among other and 3% and 2% among white, respectively. Declines in intubated respiratory support and delivery room intubation were also observed across all ethnic groups. Receipt of any maternal milk at discharge increased among EPT infants of mixed (4% per year) and white (2% per year) mothers, and among VPT infants of white mothers (1% per year). Sensitivity analyses excluding infants born <24 weeks gestation yielded consistent findings (online supplemental tables 6A and B, table 7).

Clinical outcomes by maternal ethnicity (online supplemental tables 8A and B, table 9): Among EPT admissions, mortality declined significantly for those born to white mothers. In VPT infants, BPD increased on average by 2% per year in both Asian and white groups, while severe NEC decreased by 4% per year among infants of white mothers. Survival without major morbidity declined by approximately 1% per year among VPT infants born to Asian and white mothers. In sensitivity analyses excluding infants <24 weeks, severe NEC increased on average by 20% per year among EPT infants of mixed ethnicity.

Results from the negative binomial models (online supplemental table 11) were consistent with those from the modified Poisson regression, with similar direction, magnitude and statistical inference across modelling approaches. Baseline characteristics, including gestational age, birth weight z-score, sex, multiplicity and antenatal steroid exposure, were similar between infants included in complete-case analyses and those with missing baseline data (online supplemental table 12).

## Discussion

We examined trends in EPT and VPT admissions to neonatal units, care processes and outcomes in England and Wales over an 11-year period. We found evidence of persisting ethnic variation, changing clinical practice and modest changes in clinical outcomes. Overall, EPT and VPT admissions have not fallen, but the number of admissions of babies born below 24 weeks gestation has almost doubled. Mothers of black ethnicity had EPT admission rates around two times that of Asian mothers, and almost 2.5 times higher than white mothers. There has been an increase in the proportion of EPT and VPT admissions delivered by emergency caesarean section, and a decrease in delivery room intubation, use of intubated respiratory support and PDA closure by surgery or device. These observations are consistent with clinical practice that has moved towards more frequent obstetrical intervention and less invasive postnatal neonatal care. We also identified improvements in two care processes known to benefit outcomes: more infants were born in hospitals with tertiary designation, and a greater proportion received their own mother’s milk at discharge in 2023 compared with 2013. However, changes in clinical outcomes were mixed, and in particular, the lack of progress in survival without major morbidity is disappointing.

Our study has several strengths. We used quality-assured real-world data from a national database that has a high level of completeness and reliability.^13 14^ In England and Wales, NHS hospitals provide neonatal care for all EPT and most VPT infants; hence, we were able to include all admissions to neonatal care for England and achieved similarly complete geographically defined population coverage for Wales from 2013. The availability of gestational age data enabled results to be reported separately for EPT and VPT admissions and for sensitivity analyses to be conducted excluding infants below 24 weeks gestation. To assess whether alterations in stillbirth rates might have influenced change in admissions, we included data from the ONS on total live and stillbirths over the study period. We used objective metrics and as the NNRD incorporates a consistent approach to derive complex outcomes such as BPD, NEC and severe brain injury, any likelihood of reporting bias is minimised.

We also acknowledge limitations. The longitudinal trends we report were derived from models that included birth year as a continuous variable, providing an estimate of the average annual relative change for each outcome. Because outcomes represent a prespecified set of clinically interrelated neonatal indicators, we focused on the direction and magnitude of temporal change rather than formal multiple-testing corrections, which would have inflated the type II error rate and obscured meaningful epidemiological patterns. 17% of maternal ethnicity data were missing. However, comparisons of baseline clinical characteristics between complete and incomplete cases did not suggest systematic differences reducing any concern that the complete-case analysis materially biased the conclusions. We do not know the proportion of missing data for late onset bloodstream infection where a null entry in the NNRD may represent non-occurrence or non-entry into the patient electronic patient record. We therefore did not include late onset bloodstream infection in the composite ‘survival without major morbidity’ and are unable to exclude the possibility that the significant increase in late onset infection in EPT admissions might be due to changes in data entry and better case ascertainment.

Our study, extending over 11 years, highlights persisting ethnic disparity in the lack of change in the higher rates of EPT and VPT admissions born to mothers of black ethnicity. This mirrors previous studies that have shown that compared with mothers of white ethnicity, stillbirth rates are around two times as high for mothers of black ethnicity and 60% higher for Asian mothers.^15^ It is important to note that we relied on standard NHS ethnicity classification, which in principle is self-defined. However, this construct has major weaknesses as it assumes a shared set of cultural attributes, which in modern-day Britain may not hold true. Additionally, healthcare professionals often assign ethnicity, which increases the possibility of misclassification. Taken together, our data highlight the need for research to identify the separate contributions of genetic, cultural and economic influences on perinatal outcomes.

Several national ‘ambitions’ and targets were set during the period covered by our study. These include a national ambition announced by the UK Secretary of State for Health in 2015 to reduce perinatal brain injuries by 20% by 2020 and 50% by 2030 and halve stillbirths and neonatal deaths by 2030.^16^ The ‘Saving Babies Lives’ care bundle, initiated in 2014 aimed to halve stillbirths in England from 4.7 per thousand to 2.3 per thousand by 2030.^7^ In 2019, the NHS Long Term Plan included a target to halve neonatal deaths by 2025.^17^ In the same year, following an evidence review, NHS England launched an implementation plan to improve neonatal services and reduce disparities.^18^ In addition, since 2007, Healthcare Quality Improvement Partnership has commissioned the UK National Neonatal Audit Programme to evaluate adherence to key care processes and monitor outcomes such as ROP and breast milk at discharge for preterm infants.^19^ Given these initiatives, we may have expected to see substantially improved outcomes. However, these national data indicate that though audit and quality improvement programmes can be helpful, an inherent assumption of benefit may not be justified, given their costs, the workload they can impose on clinical teams and that benefits seen in some settings may not necessarily translate to other locations, such programmes should incorporate formal evaluation of impact.

Early postnatal transfers decreased over time but remained high at around 17% of EPT and 10% of VPT admissions. This is a concern as postnatal transfer in the first days after birth increases the risk of severe brain injury in EPT infants.^20^ Our data indicate a need to improve processes for transfer of mothers to a centre with tertiary neonatal care prior to delivery of a high-risk infant.

Our data also highlight the profound impact of decreasing gestational age on the likelihood of intact survival, with less than one-fifth of EPT admissions surviving to discharge without major morbidity. This points to the need for greater effort to reduce preterm births. Though preterm birth is widely perceived as a medical issue, healthcare interventions have been largely ineffective in reducing rates. Of note, we identified a highly significant fall in EPT births during the COVID-19 pandemic.^21^ We therefore suggest that a major focus for future research should be identification of the causal determinants underpinning the strong associations between preterm birth and socioeconomic and environmental conditions, as public health policies may prove to be more effective than medical interventions.

International comparisons of perinatal and preterm outcomes are hampered by a lack of consistency in definitions and denominators, and hospital as opposed to population-based reports. Thus, data for treated ROP from the USA refer to rates of 6–7% in ‘high-risk’ or ‘low birthweight’ categories, making precise comparison with our data difficult.^22^ Caution is needed for both within and between country comparisons of outcomes in extremely low gestational age infants. Differences may not necessarily reflect quality of care, but rather varying attitudes to non-initiation or withdrawal of life-sustaining interventions and whether data on these infants are included in national statistics.

In conclusion, ethnic disparities in EPT and VPT admissions in England and Wales have persisted over the last decade. There have been modest improvements in some outcomes, but these have not translated into meaningful gains in survival without major morbidity. Our findings highlight the continuing challenge of very and extremely preterm birth.

## Supplementary material

10.1136/bmjph-2025-004256 online supplemental figure 1

10.1136/bmjph-2025-004256 online supplemental table 1

10.1136/bmjph-2025-004256 online supplemental file 1

## Data Availability

Data are available upon reasonable request.
