# Supplementary material for: Changes in admissions, care processes and outcomes for very and extremely preterm infants in England and Wales: an 11-year whole population study
Source: BMJ Public Health. 2026 Mar 31;4(1):e004256. doi: 10.1136/bmjph-2025-004256 (PMC13052741; doi:10.1136/bmjph-2025-004256)
Supplement: online supplemental table 1 [file bmjph-4-1-s002.docx]

**Supplementary Tables**

**Supplementary Table 1A**

**Extremely preterm (EPT) (<28w) admissions as a proportion of live births by maternal ethnicity and year**

Data from the Office of National Statistics for 2024 were not available at the time of writing; risk ratio and 95% confidence interval (RR; 95% CI) were estimated by modified Poisson regression; this analysis includes 21907 EPT admissions; 4225 EPT admissions were excluded because of missing maternal ethnicity data

| **Maternal Ethnicity** | **2013**  **N/livebirths (%)** | **2014**  **N/livebirths (%)** | **2015**  **N/livebirths (%)** | **2016**  **N/livebirths (%)** | **2017**  **N/livebirths (%)** | **2018**  **N/livebirths (%)** | **2019**  **N/livebirths (%)** | **2020**  **N/livebirths (%)** | **2021**  **N/livebirths (%)** | **2022**  **N/livebirths (%)** | **2023**  **N/livebirths (%)** | **Overall trend, RR (95% CI)** |
| --- | --- | --- | --- | --- | --- | --- | --- | --- | --- | --- | --- | --- |
| **Asian** | 300/77,246 (0.39) | 271/76,877 (0.35) | 280/76,976 (0.36) | 289/78,639 (0.37) | 313/76,045 (0.41) | 284/72,787 (0.39) | 264/73,874 (0.36) | 271/74,653 (0.36) | 275/75,923 (0.36) | 345/80167 (0.43) | 345/86115 (0.40) | 1.01 (0.99 to 1.02) |
| **Black** | 265/34,782 (0.76) | 268/33,541 (0.8) | 221/33,461 (0.66) | 262/33,736 (0.78) | 238/32,565 (0.73) | 227/31,321 (0.72) | 217/30,846 (0.7) | 209/29,882 (0.7) | 215/29,866 (0.72) | 241/33405 (0.72) | 243/37275 (0.65) | 1.01 (0.99 to 1.02) |
| **Mixed** | 46/36,046 (0.13) | 33/37,450 (0.09) | 49/40,433 (0.12) | 44/41,931 (0.1) | 47/41,381 (0.11) | 32/41,291 (0.08) | 38/41,918 (0.09) | 37/40,751 (0.09) | 45/42,928 (0.10) | 61/42245 (0.14) | 52/40333 (0.13) | 1.01 (0.98 to 1.04) |
| **Other** | 51/14,497 (0.35) | 42/15,232 (0.28) | 56/15,625 (0.36) | 45/16,374 (0.27) | 45/15,646 (0.29) | 64/15,845 (0.4) | 50/15,523 (0.32) | 54/14,293 (0.38) | 72/14,161 (0.51) | 60/14917 (0.39) | 73/14894 (0.49) | 1.05 (1.02 to 1.08) |
| **White** | 1617/511,750 (0.32) | 1512/504,858 (0.3) | 1554/507,829 (0.31) | 1487/499,677 (0.3) | 1403/482,439 (0.29) | 1305/468,456 (0.28) | 1255/452,248 (0.28) | 1078/431,652 (0.25) | 1261/440,320 (0.29) | 1244/413334 (0.30) | 1252/391473 (0.32) | 0.99 (0.98 to 1.0) |
| **Missing** | 179 | 281 | 324 | 369 | 372 | 461 | 522 | 514 | 461 | 402 | 340 |  |

**Supplementary Table 1B**

**Very preterm (VPT) (28w to <32w) admissions as a proportion of live births by maternal ethnicity and year**

Data from the Office of National Statistics for 2024 were not available at the time of writing; risk ratio and 95% confidence interval (RR; 95% CI) were estimated by modified Poisson regression; this analysis includes 46325 VPT admissions; 9464 VPT admissions were excluded because of missing maternal ethnicity data

| **Maternal Ethnicity** | **2013**  **N/livebirths (%)** | **2014**  **N/livebirths (%)** | **2015**  **N/livebirths (%)** | **2016**  **N/livebirths (%)** | **2017**  **N/livebirths (%)** | **2018**  **N/livebirths (%)** | **2019**  **N/livebirths (%)** | **2020**  **N/livebirths (%)** | **2021**  **N/livebirths (%)** | **2022**  **N/livebirths (%)** | **2023**  **N/livebirths (%)** | **Overall trend, RR (95% CI)** |
| --- | --- | --- | --- | --- | --- | --- | --- | --- | --- | --- | --- | --- |
| **Asian** | 663/77,246 (0.86) | 654/76,877 (0.85) | 579/76,976 (0.75) | 595/78,639 (0.76) | 566/76,045 (0.74) | 497/72,787 (0.68) | 541/73,874 (0.73) | 508/74,653 (0.68) | 536/75,923 (0.7) | 537/80167 (0.67) | 647/86115 (0.75) | 0.98 (0.97 to 0.99) |
| **Black** | 402/34,782 (1.16) | 385/33,541 (1.15) | 399/33,461 (1.19) | 340/33,736 (1.01) | 365/32,565 (1.12) | 307/31,321 (0.98) | 313/30,846 (1.01) | 291/29,882 (0.97) | 296/29,866 (0.99) | 307/33405 (0.91) | 374/37275 (1.0) | 0.98 (0.97 to 0.99) |
| **Mixed** | 73/36,046 (0.2) | 53/37,450 (0.14) | 86/40,433 (0.21) | 83/41,931 (0.2) | 101/41,381 (0.24) | 79/41,291 (0.19) | 83/41,918 (0.2) | 78/40,751 (0.19) | 71/42,928 (0.17) | 99/42245 (0.23) | 93/40333 (0.23) | 1.01 (0.99 to 1.04) |
| **Other** | 90/14,497 (0.62) | 83/15,232 (0.54) | 76/15,625 (0.49) | 99/16,374 (0.6) | 87/15,646 (0.56) | 89/15,845 (0.56) | 106/15,523 (0.68) | 101/14,293 (0.71) | 109/14,161 (0.76) | 100/14917 (0.67) | 121/14894 (0.81) | 1.04 (1.02 to 1.06) |
| **White** | 3726/511,750 (0.73) | 3514/504,858 (0.7) | 3522/507,829 (0.69) | 3437/499,677 (0.69) | 3427/482,439 (0.71) | 3058/468,456 (0.65) | 2792/452,248 (0.62) | 2464/431,652 (0.57) | 2740/440,320 (0.62) | 2856/413334 (0.69) | 2727/391473 (0.70) | 0.99 (0.98 to 0.99) |
| **Missing** | 456 | 663 | 864 | 898 | 894 | 1028 | 1218 | 1139 | 945 | 702 | 657 |  |

**Supplementary Table 2**

**Stillbirths by maternal ethnicity and year**

Data from the Office of National Statistics for 2024 were not available at the time of writing. Absolute number and rate per thousand total births above 24w gestation (data from the Office for National Statistics)

|  | **2013**  **N (Rate per thousand total births)** | **2014**  **N (Rate per thousand total births)** | **2015**  **N (Rate per thousand total births)** | **2016**  **N (Rate per thousand total births)** | **2017**  **N (Rate per thousand total births)** | **2018**  **N (Rate per thousand total births)** | **2019**  **N (Rate per thousand total births)** | **2020**  **N (Rate per thousand total births)** | **2021**  **N (Rate per thousand total births)** | **2022**  **N (Rate per thousand total births)** | **2023**  **N (Rate per thousand total births)** |
| --- | --- | --- | --- | --- | --- | --- | --- | --- | --- | --- | --- |
| **Asian** | 494 (6.4) | 496 (6.4) | 460 (5.9) | 458 (5.8) | 441 (5.8) | 384 (5.2) | 381 (5.1) | 390 (5.2) | 382 (5.0) | 377 (4.7) | 450 (5.2) |
| **Black** | 261 (7.4) | 249 (7.4) | 279 (8.3) | 258 (7.6) | 236 (7.2) | 229 (7.3) | 221 (7.1) | 189 (6.3) | 208 (6.9) | 217 (6.5) | 237 (6.4) |
| **Mixed** | 169 (4.7) | 177 (4.7) | 185 (4.6) | 194 (4.6) | 168 (4.0) | 163 (3.9) | 125 (3.0) | 143 (3.5) | 188 (4.4) | 169 (4.0) | 154 (3.8) |
| **Other** | 78 (5.4) | 69 (4.5) | 72 (4.6) | 96 (5.8) | 70 (4.5) | 87 (5.5) | 59 (3.8) | 56 (3.9) | 64 (4.5) | 67 (4.5) | 70 (4.7) |
| **White** | 2,071 (4.0) | 2,075 (4.1) | 1,962 (3.8) | 1,898 (3.8) | 1,766 (3.6) | 1,640 (3.5) | 1,548 (3.4) | 1,404 (3.2) | 1,588 (3.6) | 1462 (3.5) | 1298 (3.3) |
| **Not stated** | 173 (7.2) | 159 (5.9) | 143 (6.4) | 167 (6.6) | 149 (4.8) | 160 (5.9) | 151 (5.9) | 160 (7.2) | 149 (7.1) | 116 (5.5) |  |

**Supplementary Table 3**

**Sensitivity analysis**

**Extremely preterm (EPT) admissions excluding births <24w gestation; care processes by year**

Adjusted risk ratios and 95% confidence intervals (aRR; 95% CI) estimated by modified Poisson regression; adjustment variables are sex, gestational age (weeks), birth weight z-score, and multiplicity; this analysis includes 23089 EPT admissions

|  | **2013**  **% (95% CI)** | **2014**  **% (95% CI)** | **2015**  **% (95% CI)** | **2016**  **% (95% CI)** | **2017**  **% (95% CI)** | **2018**  **% (95% CI)** | **2019**  **% (95% CI)** | **2020**  **% (95% CI)** | **2021**  **% (95% CI)** | **2022**  **% (95% CI)** | **2023**  **% (95% CI)** | **Overall trend, aRR (95% CI)** |
| --- | --- | --- | --- | --- | --- | --- | --- | --- | --- | --- | --- | --- |
| **Antenatal steroids** | 89.1 (87.6 to 90.5) | 90.7 (89.8 to 91.6) | 89.9 (88.4 to 91.4) | 90.0 (88.4 to 91.6) | 90.8 (89.5 to 92.2) | 91.0 (89.8 to 92.2) | 92.3 (91.3 to 93.3) | 93.6 (91.9 to 95.2) | 93.6 (92.4 to 94.8) | 90.5 (89 to 92) | 92.1 (90.9 to 93.3) | 1.0 (1.0 to 1.0) |
| **Birth in a hospital with a neonatal intensive care unit** | 67.4 (65.6 to 69.3) | 67.6 (66.2 to 69.0) | 68.4 (65.9 to 70.9) | 69.5 (67.5 to 71.5) | 69.0 (67.3 to 70.7) | 71.7 (69.6 to 73.8) | 73.6 (71.5 to 75.7) | 76.1 (73.8 to 78.4) | 75.2 (72.8 to 77.6) | 74.8 (73.3 to 76.2) | 75.2 (73.2 to 77.1) | 1.01 (1.01 to 1.02) |
| **Emergency C-Section** | 39.0 (36.5 to 41.4) | 38.6 (36.5 to 40.6) | 40.4 (38.3 to 42.4) | 40.9 (38.7 to 43.0) | 40.4 (37.6 to 43.1) | 42.3 (40.3 to 44.3) | 44.4 (41.4 to 47.5) | 46.7 (44.3 to 49.0) | 52.2 (49.7 to 54.8) | 51.4 (49.9 to 52.9) | 56.8 (54.5 to 59.0) | 1.03 (1.03 to 1.04) |
| **Intubation in delivery room** | 87.4 (85.7 to 89.1) | 86.4 (85 to 87.8) | 86.7 (85.4 to 87.9) | 83.4 (81.8 to 85.0) | 81.8 (80.4 to 83.2) | 77.4 (76.0 to 78.8) | 74.6 (72.0 to 77.3) | 71.2 (69.2 to 73.2) | 66.0 (62.8 to 69.3) | 64.4 (61.4 to 67.5) | 60.1 (57.9 to 62.3) | 0.96 (0.96 to 0.96) |
| **Transfer within first 48 hours (any direction)** | 21.0  (19.1 to 22.9) | 21.5  (19.7 to 23.3) | 20.9  (18.6 to 23.2) | 20.9  (18.9 to 22.9) | 21.2  (19.7 to 22.7) | 20.4  (18.9 to 21.9) | 18.4  (16.8 to 20) | 15.9  (13.9 to 17.9) | 17.8  (15.4 to 20.2) | 17.3  (16.2 to 18.4) | 17.0 (15.3 to 18.7) | 0.98 (0.97 to 0.99) |
| **Transfer within first 48 hours (upwards)** | 18.7  (17 to 20.4) | 19.3  (17.8 to 20.8) | 18.8  (16.8 to 20.8) | 18.5  (16.7 to 20.3) | 19.1  (17.4 to 20.8) | 18.6  (16.8 to 20.4) | 16.7  (15.1 to 18.3) | 15.2  (13.2 to 17.2) | 16.4  (13.9 to 18.9) | 15.9  (14.9 to 16.9) | 15.6 (14.1 to 17.3) | 0.98 (0.97 to 0.99) |
| **Transfer within first 48 hours (horizontal)** | 1.9 (1.4 to 2.4) | 2.0 (1.3 to 2.7) | 1.9 (1.4 to 2.4) | 2.2 (1.9 to 2.5) | 2.1 (1.5 to 2.7) | 1.6 (1.2 to 2.0) | 1.3 (0.9 to 1.7) | 0.8 (0.6 to 1.0) | 1.4 (0.9 to 1.9) | 1.2 (0.7 to 1.7) | 0.8 (0.4 to 1.3) | 0.91 (0.88 to 0.94) |
| **Transfer within first 48 hours (downwards)** | 1.0 (0.6 to 1.4) | 1.0 (0.7 to 1.3) | 0.5 (0.5 to 0.5) | 0.8 (0.5 to 1.1) | 0.9 (0.5 to 1.3) | 0.8 (0.3 to 1.3) | 1.3 (1.2 to 1.4) | 0.6 (0.6 to 0.6) | 0.6 (0.5 to 0.7) | 0.6 (0.5 to 0.7) | 0.1 (0 to 0.4) | 0.81 (0.73 to 0.90) |
| **Intubated respiratory support** | 96.7 (96.1 to 97.3) | 96.2 (95.5 to 96.9) | 95.6 (94.5 to 96.6) | 94.8 (93.8 to 95.8) | 95.0 (94.0 to 96.0) | 93.5 (92.7 to 94.3) | 92.4 (91.5 to 93.3) | 91.8 (90.7 to 92.9) | 89.0 (87.8 to 90.3) | 88.6 (86.9 to 90.3) | 86.8 (85.2 to 88.3) | 0.99 (0.99 to 0.99) |
| **PDA closure** | 5.3 (4.2 to 6.5) | 3.4 (3.0 to 3.9) | 4.0 (3.0 to 4.9) | 3.2 (2.6 to 3.8) | 2.6 (1.9 to 3.2) | 2.6 (1.8 to 3.4) | 1.6 (1.1 to 2.1) | 1.3 (0.9 to 1.7) | 1.5 (1.1 to 2.0) | 0.6 (0.0 to 1.1) | 0.5 (0.2 to 1.0) | 0.83 (0.80 to 0.85) |
| **Receiving any own mother’s milk at discharge** | 39.2 (37.4 to 41.1) | 38.7 (36.7 to 40.7) | 38.3 (35.9 to 40.7) | 40 (38.0 to 42.1) | 39.8 (37.4 to 42.2) | 42.9 (40.0 to 45.8) | 42.0 (39.0 to 45.0) | 45.6 (42.7 to 48.6) | 42.4 (40.1 to 44.7) | 44.1 (41.5 to 46.8) | 55.6 (53.1 to 58.1) | 1.02 (1.01 to 1.02) |

**Supplementary Table 4A**

**Clinical outcomes by year; extremely preterm (EPT) admissions**

Adjusted risk ratio and 95% confidence interval (aRR; 95% CI) estimated by modified Poisson regression; reference year is 2013; adjustment variables are sex, gestational age (weeks), birth weight z-score, and multiplicity; this analysis included 26132 EPT admissions

|  | **2013**  **% (95% CI)** | **2014**  **% (95% CI)** | **2015**  **% (95% CI)** | **2016**  **% (95% CI)** | **2017**  **% (95% CI)** | **2018**  **% (95% CI)** | **2019**  **% (95% CI)** | **2020**  **% (95% CI)** | **2021**  **% (95% CI)** | **2022**  **% (95% CI)** | **2023**  **% (95% CI)** | **Overall trend** |
| --- | --- | --- | --- | --- | --- | --- | --- | --- | --- | --- | --- | --- |
| **Mortality**  **aRR (95% CI)** | 21.8 (20.1 to 23.4)  **Ref** | 21.2 (20.0 to 22.8)  0.96 (0.87 to 1.06) | 20.2 (18.6 to 21.8)  0.91 (0.82 to 1.0) | 20.0 (18.5 to 21.6)  0.94 (0.85 to 1.04) | 20.1 (18.5 to 21.7)  0.89 (0.81 to 0.99) | 20.0 (18.4 to 21.7)  0.89 (0.81 to 0.98) | 20.3 (18.7 to 22.0)  0.87 (0.79 to 0.96) | 20.1 (18.4 to 21.8)  0.82 (0.74 to 0.92) | 21.8 (20.1 to 23.4)  0.93 (0.85 to 1.03) | 22.1 (20.4 to 23.8)  0.84 (0.76 to 0.93) | 22.3 (20.6 to 24.0)  0.87 (0.79 to 0.96) | 20.8 (20.4 to 21.3)  0.98 (0.97 to 0.99) |
| **Bronchopulmonary dysplasia**  **aRR (95% CI)** | 54.9 (53.0 to 56.9)  **Ref** | 55.7 (53.7 to 57.7)  1.01 (0.97 to 1.06) | 54.7 (52.7 to 56.7)  1.0 (0.96 to 1.04) | 54.5 (52.5 to 56.4)  1.0 (0.96 to 1.04) | 56.2 (54.2 to 58.2)  1.03 (0.98 to 1.07) | 55.2 (53.2 to 57.2)  1.0 (0.96 to 1.04) | 58.4 (56.4 to 60.4)  1.06 (1.02 to 1.10) | 59.6 (57.5 to 61.6)  1.10 (1.05 to 1.14) | 56.5 (54.4 to 58.5)  1.04 (1.0 to 1.08) | 56.2 (54.2 to 58.2)  1.05 (1.0 to 1.09) | 58.1 (56.0 to 60.1)  1.08 (1.04 to 1.12) | 56.1 (55.5 to 56.7)  1.0 (1.0 to 1.01) |
| **Severe brain injury**  **aRR (95% CI)** | 22.8 (21.2 to 24.5)  **Ref** | 23.3 (21.6 to 25.0)  1.02 (0.93 to 1.12) | 23.1 (21.4 to 24.7)  1.0 (0.91 to 1.10) | 23.2 (21.5 to 24.8)  1.02 (0.93 to 1.12) | 25.3 (23.5 to 27.0)  1.10 (1.0 to 1.20) | 24.1 (22.4 to 25.9)  1.04 (0.94 to 1.14) | 23.3 (21.6 to 25.0)  1.0 (0.91 to 1.10) | 22.5 (20.7 to 24.3)  0.96 (0.87 to 1.06) | 24.1 (22.3 to 25.8)  1.05 (0.95 to 1.15) | 24.2 (22.5 to 25.9)  0.99 (0.90 to 1.09) | 23.3 (21.6 to 25.0)  1.01 (0.92 to 1.11) | 23.4 (22.9 to 23.9)  1.0 (0.99 to 1.01) |
| **Late onset bloodstream infection**  **aRR (95% CI)** | 9.6 (8.4 to 10.8)  **Ref** | 8.2 (7.1 to 9.3)  0.85 (0.71 to 1.0) | 8.6 (7.5 to 9.7)  0.88 (0.74 to 1.05) | 10.5 (9.3 to 11.7)  1.10 (0.94 to 1.3) | 10.6 (9.4 to 11.8)  1.10 (0.93 to 1.3) | 10.7 (9.4 to 11.9)  1.08 (0.92 to 1.27) | 11.4 (10.1 to 12.7)  1.15 (0.98 to 1.36) | 13.9 (12.4 to 15.4)  1.40 (1.20 to 1.64) | 11.6 (10.3 to 12.9)  1.19 (1.01 to 1.39) | 11.4 (11.1 to 13.7)  1.24 (1.06 to 1.46) | 10.5 (9.2 to 11.7)  1.06 (0.89 to 1.25) | 10.4 (10.1 to 10.8)  1.03 (1.02 to 1.04) |
| **Severe necrotising enterocolitis**  **aRR (95% CI)** | 8.3 (7.2 to 9.4)  **Ref** | 7.8 (6.8 to 8.9)  0.94 (0.78 to 1.13) | 8.2 (7.1 to 9.3)  0.98 (0.81 to 1.18) | 7.8 (6.7 to 8.8)  0.95 (0.79 to 1.14) | 10.0 (8.8 to 11.1)  1.18 (0.99 to 1.41) | 8.0 (6.9 to 9.1)  0.93 (0.77 to 1.12) | 9.7 (8.5 to 10.9)  1.12 (0.94 to 1.34) | 8.5 (7.3 to 9.6)  0.96 (0.79 to 1.17) | 8.1 (7.0 to 9.2)  0.96 (0.79 to 1.16) | 8.1 (7.0 to 9.2)  0.90 (0.74 to 1.10) | 7.0 (6.0 to 8.1)  0.83 (0.68 to 1.01) | 8.2 (7.9 to 8.5)  0.99 (0.98 to 1.0) |
| **Treated retinopathy of prematurity**  **aRR (95% CI)** | 11.6 (10.3 to 12.9)  **Ref** | 12.1 (10.8 to 13.4)  1.03 (0.89 to 1.19) | 12.8 (11.5 to 14.2)  1.09 (0.94 to 1.25) | 9.5 (8.4 to 10.7)  0.83 (0.71 to 0.97) | 9.0 (7.8 to 10.1)  0.75 (0.64 to 0.88) | 10.0 (8.8 to 11.2)  0.81 (0.69 to 0.95) | 11.3 (10.0 to 12.6)  0.93 (0.80 to 1.08) | 11.3 (9.9 to 12.6)  0.94 (0.81 to 1.10) | 12.3 (11.0 to 13.7)  1.03 (0.89 to 1.19) | 11.4 (10.0 to 13.7)  0.99 (0.86 to 1.15) | 11.8 (10.5 to 13.2)  0.98 (0.84 to 1.13) | 11.2 (10.8 to 11.5)  0.99 (0.98 to 1.0) |
| **Survival without major morbidity**  **aRR (95% CI)** | 18.3 (16.8 to 19.8)  **Ref** | 19.7 (18.1 to 21.2)  1.08 (0.97 to 1.19) | 20.3 (18.7 to 21.9)  1.10 (1.0 to 1.22) | 20.9 (19.3 to 22.6)  1.10 (0.99 to 1.21) | 18.7 (17.1 to 20.3)  1.02 (0.92 to 1.13) | 21.0 (19.4 to 22.7)  1.18 (1.07 to 1.31) | 18.0 (16.4 to 19.5)  1.03 (0.93 to 1.14) | 17.6 (16.0 to 19.2)  0.99 (0.89 to 1.10) | 18.4 (16.8 to 20.0)  1.0 (0.91 to 1.12) | 18.3 (16.7 to 19.8)  1.07 (0.96 to 1.19) | 16.9 (15.4 to 18.5)  0.98 (0.88 to 1.09) | 18.9 (18.5 to 19.4)  0.99 (0.98 to 1.0) |

**Supplementary Table 4B**

**Clinical outcomes by year; very preterm (VPT) admissions**

Adjusted risk ratio and 95% confidence interval (aRR; 95% CI) estimated by modified Poisson regression; reference year is 2013; adjustment variables are sex, gestational age (weeks), birth weight z-score, and multiplicity; this analysis included 55789 VPT admissions

|  | **2013**  **% (95% CI)** | **2014**  **% (95% CI)** | **2015**  **% (95% CI)** | **2016**  **% (95% CI)** | **2017**  **% (95% CI)** | **2018**  **% (95% CI)** | **2019**  **% (95% CI)** | **2020**  **% (95% CI)** | **2021**  **% (95% CI)** | **2022**  **% (95% CI)** | **2023**  **% (95% CI)** | **Overall trend** |
| --- | --- | --- | --- | --- | --- | --- | --- | --- | --- | --- | --- | --- |
| **Mortality**  **aRR (95% CI)** | 3.4 (2.9 to 3.9)  **Ref** | 2.9 (2.4 to 3.3)  0.87 (0.71 to 1.08) | 3.1 (2.6 to 3.5)  0.92 (0.75 to 1.13) | 2.9 (2.5 to 3.4)  0.87 (0.71 to 1.07) | 3.0 (2.6 to 3.5)  0.90 (0.73 to 1.10) | 2.6 (2.2 to 3.1)  0.78 (0.63 to 0.97) | 2.8 (2.4 to 3.3)  0.84 (0.68 to 1.04) | 2.8 (2.3 to 3.2)  0.80 (0.64 to 1.0) | 2.9 (2.4 to 3.4)  0.86 (0.69 to 1.07) | 2.9 (2.4 to 3.4)  0.87 (0.70 to 1.08) | 2.6 (2.2 to 3.1)  0.76 (0.61 to 0.96) | 2.9 (2.7 to 3.0)  0.99 (0.98 to 1.0) |
| **Bronchopulmonary dysplasia**  **aRR (95% CI)** | 15.9 (14.9 to 16.9)  **Ref** | 16.9 (15.9 to 17.9)  1.08 (0.99 to 1.16) | 16.5 (15.5 to 17.5)  1.05 (0.97 to 1.14) | 16.4 (15.4 to 17.4)  1.03 (0.96 to 1.11) | 17.0 (16.0 to 18.0)  1.05 (0.97 to 1.14) | 17.9 (16.8 to 18.9)  1.13 (1.05 to 1.22) | 17.7 (16.6 to 18.7)  1.11 (1.02 to 1.19) | 18.7 (17.6 to 19.9)  1.17 (1.08 to 1.26) | 17.4 (16.3 to 18.5)  1.11 (1.02 to 1.20) | 18.4 (17.3 to 19.6)  1.18 (1.09 to 1.27) | 19.1 (18.0 to 20.2)  1.20 (1.12 to 1.30) | 17.5 (17.2 to 17.8)  1.01 (1.0 to 1.02) |
| **Severe brain Injury**  **aRR (95% CI)** | 5.6 (5.0 to 6.2)  **Ref** | 6.2 (5.6 to 6.9)  1.13 (0.97 to 1.31) | 5.8 (5.2 to 6.4)  1.05 (0.90 to 1.22) | 6.1 (5.4 to 6.7)  1.09 (0.93 to 1.26) | 5.6 (4.9 to 6.2)  0.99 (0.85 to 1.16) | 6.1 (5.4 to 6.7)  1.09 (0.93 to 1.27) | 5.7 (5.1 to 6.3)  1.02 (0.88 to 1.20) | 5.7 (5.0 to 6.3)  1.0 (0.85 to 1.17) | 5.7 (5.1 to 6.4)  1.02 (0.87 to 1.20) | 6.0 (5.3 to 6.7)  1.07 (0.92 to 1.25) | 6.0 (5.3 to 6.7)  1.07 (0.92 to 1.26) | 5.8 (5.6 to 6.0)  0.99 (0.98 to 1.0) |
| **Late onset bloodstream infection**  **aRR (95% CI)** | 1.8 (1.5 to 2.2)  **Ref** | 2.0 (1.6 to 2.4)  1.12 (0.85 to 1.46) | 2.0 (1.6 to 2.3)  1.09 (0.83 to 1.43) | 1.8 (1.4 to 2.1)  0.97 (0.74 to 1.28) | 2.0 (1.6 to 2.3)  1.06 (0.81 to 1.39) | 1.8 (1.4 to 2.1)  0.97 (0.73 to 1.28) | 2.0 (1.6 to 2.4)  1.09 (0.83 to 1.43) | 2.1 (1.7 to 2.5)  1.12 (0.85 to 1.47) | 1.9 (1.5 to 2.3)  1.06 (0.80 to 1.40) | 1.7 (1.3 to 2.0)  0.92 (0.69 to 1.24) | 1.6 (1.2 to 1.9)  0.87 (0.64 to 1.17) | 1.8 (1.7 to 1.9)  0.99 (0.96 to 1.01) |
| **Severe necrotising enterocolitis**  **aRR (95% CI)** | 1.4 (1.1 to 1.7)  **Ref** | 1.6 (1.3 to 1.9)  1.15 (0.85 to 1.55) | 1.4 (1.0 to 1.7)  0.96 (0.70 to 1.32) | 1.4 (1.1 to 1.7)  0.98 (0.72 to 1.34) | 1.4 (1.1 to 1.7)  0.94 (0.68 to 1.29) | 1.1 (0.8 to 1.4)  0.81 (0.58 to 1.13) | 1.4 (1.1 to 1.8)  1.02 (0.75 to 1.40) | 1.3 (1.0 to 1.7)  0.92 (0.66 to 1.29) | 1.1 (0.8 to 1.4)  0.80 (0.56 to 1.13) | 1.2 (0.9 to 1.5)  0.87 (0.62 to 1.22) | 0.8 (0.5 to 1.0)  0.55 (0.37 to 0.81) | 1.3 (1.2 to 1.4)  0.96 (0.94 to 0.98) |
| **Treated retinopathy of prematurity**  **aRR (95% CI)** | 0.8 (0.6 to 1.0)  **Ref** | 0.8 (0.6 to 1.1)  1.01 (0.67 to 1.53) | 0.8 (0.5 to 1.0)  0.91 (0.60 to 1.39) | 0.6 (0.3 to 0.7)  0.67 (0.42 to 1.07) | 0.3 (0.2 to 0.4)  0.35 (0.20 to 0.62) | 0.4 (0.2 to 0.5)  0.46 (0.27 to 0.79) | 0.4 (0.3 to 0.6)  0.55 (0.33 to 0.91) | 0.5 (0.3 to 0.8)  0.66 (0.41 to 1.08) | 0.6 (0.4 to 0.8)  0.74 (0.46 to 1.19) | 0.5 (0.3 to 0.7)  0.60 (0.36 to 0.99) | 0.7 (0.5 to 1.0)  0.88 (0.56 to 1.38) | 0.6 (0.5 to 0.7)  0.96 (0.93 to 1.0) |
| **Survival without major morbidity**  **aRR (95% CI)** | 75.4 (75.2 to 77.5)  **Ref** | 75.4 (74.2 to 76.5)  0.98 (0.97 to 0.99) | 76.2 (75.0 to 77.3)  0.99 (0.98 to 1.0) | 76.4 (75.3 to 77.5)  0.99 (0.98 to 1.01) | 76.4 (75.3 to 77.6)  1.0 (0.99 to 1.02) | 75.8 (74.6 to 77.0)  0.99 (0.97 to 1.0) | 75.8 (74.6 to 77.0)  0.99 (0.98 to 1.01) | 74.2 (73.0 to 75.5)  0.98 (0.96 to 0.99) | 75.8 (74.6 to 77.1)  0.98 (0.97 to 1.0) | 75.1 (73.9 to 76.4)  0.98 (0.97 to 0.99) | 74.2 (72.9 to 75.5)  0.97 (0.95 to 0.98) | 75.6 (75.2 to 76.0)  0.99 (0.99 to 0.99) |

**Supplementary Table 5**

**Sensitivity analysis**

**Extremely preterm (EPT) admissions excluding births <24w gestation, clinical outcomes by year**

Adjusted risk ratios and 95% confidence intervals (aRR; 95% CI) estimated by modified Poisson regression; adjustment variables are sex, gestational age (weeks), birth weight z-score, and multiplicity; this analysis includes 23089 EPT admissions

|  | **2013**  **% (95% CI)** | **2014**  **% (95% CI)** | **2015**  **% (95% CI)** | **2016**  **% (95% CI)** | **2017**  **% (95% CI)** | **2018**  **% (95% CI)** | **2019**  **% (95% CI)** | **2020**  **% (95% CI)** | **2021**  **% (95% CI)** | **2022**  **% (95% CI)** | **2023**  **% (95% CI)** | **Overall trend, aRR (95% CI)** |
| --- | --- | --- | --- | --- | --- | --- | --- | --- | --- | --- | --- | --- |
| **Mortality** | 18.6 (16.6 to 20.6) | 17.4 (16.4 to 18.5) | 16.5 (14.5 to 18.5) | 17 (15.1 to 18.9) | 16.2 (14.9 to 17.6) | 15.5 (13.6 to 17.4) | 15.7 (14.3 to 17.1) | 14.8 (12.6 to 16.9) | 16.1 (14.9 to 17.3) | 14.4 (12.9 to 15.9) | 15.6 (14.0 to 17.3) | 0.98 (0.97 to 0.99) |
| **Bronchopulmonary dysplasia** | 55.9 (53.1 to 58.6) | 56.4 (54.8 to 57.9) | 55.5 (53.5 to 57.5) | 55.2 (53.2 to 57.3) | 57.2 (55.6 to 58.7) | 56.2 (54.2 to 58.3) | 59.4 (56.1 to 62.7) | 61.2 (59.3 to 63.2) | 58 (55.4 to 60.7) | 58.7 (56.5 to 61) | 61.3 (59.0 to 63.4) | 1.0 (1.0 to 1.01) |
| **Severe brain Injury** | 21.6 (19.2 to 24) | 22 (20.6 to 23.3) | 21.9 (20.3 to 23.6) | 21.7 (20.4 to 23) | 23.9 (22.6 to 25.3) | 23.7 (21.7 to 25.6) | 21.7 (20.1 to 23.4) | 20.7 (18.3 to 23.1) | 22.1 (20.1 to 24) | 20.7 (19.2 to 22.1) | 21.5 (19.7 to 23.4) | 0.99 (0.99 to 1.0) |
| **Late onset bloodstream infection** | 9.3 (8.1 to 10.6) | 7.3 (6.3 to 8.4) | 8.4 (6.8 to 10.1) | 9.6 (8.3 to 10.8) | 9.5 (8.4 to 10.7) | 10.4 (9.2 to 11.7) | 11 (9.5 to 12.4) | 12.5 (11.1 to 14) | 10.4 (9.4 to 11.5) | 10.9 (9.6 to 12.3) | 9.2 (8.0 to 10.6) | 1.03 (1.02 to 1.04) |
| **Severe necrotising enterocolitis** | 8.1 (6.7 to 9.5) | 7.3 (6.3 to 8.3) | 7.9 (6.3 to 9.4) | 7.4 (6.8 to 8.0) | 9.2 (7.4 to 11.1) | 7.6 (6.4 to 8.8) | 9.3 (8.6 to 10.1) | 7.4 (6.2 to 8.7) | 7.4 (6.4 to 8.4) | 6.8 (6.0 to 7.6) | 6.4 (5.4 to 7.6) | 0.98 (0.97 to 1.0) |
| **Treated retinopathy of prematurity** | 10.9 (9.9 to 12.0) | 11.1 (9.9 to 12.3) | 12.3 (10.4 to 14.2) | 8.5 (7.5 to 9.5) | 8.3 (6.9 to 9.6) | 8.9 (7.4 to 10.5) | 10.1 (8.9 to 11.3) | 9.8 (8.3 to 11.3) | 11.1 (9.7 to 12.5) | 10.7 (9.4 to 12.0) | 10.5 (9.1 to 11.9) | 0.99 (0.98 to 1.01) |
| **Survival without major morbidity** | 19.7 (17.9 to 21.4) | 21.5 (19.7 to 23.4) | 22 (20.1 to 24.0) | 22.6 (20.2 to 24.9) | 20.7 (19.4 to 22.1) | 23.5 (21.6 to 25.5) | 20.4 (17.9 to 22.8) | 20.2 (18.1 to 22.3) | 20.9 (18.9 to 22.9) | 20.9 (19 to 22.9) | 20.0 (18.2 to 21.8) | 0.99 (0.98 to 1.0) |

**Supplementary Table 6A**

**Extremely preterm (EPT) admissions (<28w gestation), care processes by maternal ethnicity and year**

Adjusted risk ratio and 95% confidence interval (aRR; 95% CI) estimated by modified Poisson regression; adjustment variables are sex, gestational age (weeks), birth weight z-score, and multiplicity; a missing confidence interval indicates that only one individual with that outcome was recorded; this analysis includes 21907 EPT admissions; 4225 EPT admissions were excluded because of missing ethnicity data

|  | **Maternal Ethnicity** | **2013**  **% (95% CI)** | **2014**  **% (95% CI)** | **2015**  **% (95% CI)** | **2016**  **% (95% CI)** | **2017**  **% (95% CI)** | **2018**  **% (95% CI)** | **2019**  **% (95% CI)** | **2020**  **% (95% CI)** | **2021**  **% (95% CI)** | **2022**  **% (95% CI)** | **2023**  **% (95% CI)** | **Overall trend, aRR (95% CI)** |
| --- | --- | --- | --- | --- | --- | --- | --- | --- | --- | --- | --- | --- | --- |
| **Antenatal steroids** | **Asian** | 85.6 (82.4 to 88.9) | 89.6 (84.8 to 94.3) | 89.4 (84.9 to 93.9) | 92  (87.9 to 96) | 87.6 (83.3 to 91.9) | 89.7 (87.2 to 92.3) | 91.9 (87.5 to 96.2) | 93.9 (89.5 to 98.4) | 97.8 (95.5 to 100) | 88.8 (85.7 to 91.8) | 93.6 (90.5 to 96.0) | 1.0 (1.0 to 1.01) |
|  | **Black** | 90.2 (86.4 to 94.1) | 89.4 (86.2 to 92.7) | 89.9 (85.4 to 94.3) | 88.3 (82.7 to 93.9) | 90.5 (86.5 to 94.4) | 89.4 (85.1 to 93.7) | 95.1 (92.4 to 97.8) | 94 (90.4 to 97.5) | 93.2 (89.6 to 96.8) | 94.7 (91.7 to 97.7) | 91.4 (87.1 to 94.6) | 1.0 (1.0 to 1.01) |
|  | **Mixed** | 72.8 (51.8 to 93.7) | 97.7 (93.3 to 102.2) | 72.9 (57 to 88.9) | 87.2 (74.7 to 99.8) | 87.2 (77.4 to 97) | 89.7 (79 to 100.4) | 97.2 (93.5 to 100.9) | 96.2 (91.1 to 101.3) | 91.9 (83.7 to 100.2) | 83.7 (70.9 to 96.4) | 94.2 (84.0 to 98.8) | 1.01 (1.0 to 1.01) |
|  | **Other** | 82.3 (69 to 95.7) | 89.6 (79.8 to 99.3) | 93.2 (85.7 to 100.7) | 76.6 (56.7 to 96.5) | 78.9 (62 to 95.7) | 92.3 (85.5 to 99.1) | 90.5 (79.5 to 101.4) | 92.8 (85.3 to 100.2) | 86.6 (80.1 to 93.1) | 82.9 (73.8 to 91.9) | 86.3 (76.2 to 93.2) | 1.0 (0.99 to 1.01) |
|  | **White** | 88.3 (86.1 to 90.5) | 89.6 (88.2 to 91) | 89.7 (87.7 to 91.7) | 89 (87.8 to 90.2) | 91 (89.5 to 92.5) | 90.3 (89 to 91.6) | 90.6 (89.1 to 92) | 91.7 (90 to 93.5) | 92.2 (90.8 to 93.7) | 89 (86.7 to 91.3) | 89.9 (88.1 to 91.5) | 1.0 (1.0 to 1.0) |
| **Birth in hospital with neonatal intensive care unit** | **Asian** | 72.9 (66.8 to 79) | 75.8 (70.6 to 80.9) | 72 (64.5 to 79.4) | 77.2 (71.9 to 82.4) | 76.4 (71.6 to 81.2) | 75.1 (68.6 to 81.6) | 75.9 (71.1 to 80.7) | 81  (75.9 to 86) | 81.8 (77.7 to 86) | 77.8 (73.3 to 82.3) | 79.4 (74.8 to 83.6) | 1.01 (1.0 to 1.01) |
|  | **Black** | 70.1 (62.8 to 77.4) | 74.5 (70.4 to 78.6) | 74.2 (68.1 to 80.2) | 72.7 (66.8 to 78.5) | 72.6 (65.7 to 79.5) | 73.4 (68.8 to 78) | 80 (76.2 to 83.9) | 76.9 (72.1 to 81.7) | 77.7 (70.6 to 84.7) | 78.8 (72.1 to 85.6) | 86.0 (81.0 to 90.0) | 1.01 (1.0 to 1.02) |
|  | **Mixed** | 71.1 (54.5 to 87.8) | 75.8 (57.5 to 94) | 61.4 (45.9 to 76.9) | 84.6 (72.4 to 96.8) | 76.5 (65.4 to 87.6) | 74.2 (55.9 to 92.4) | 72 (57.3 to 86.6) | 80.3 (60.7 to 99.9) | 84.2 (67.9 to 100.4) | 71.1 (59.2 to 83.1) | 75.0 (61.0 to 86.0) | 1.01 (0.99 to 1.02) |
|  | **Other** | 74.2 (58.8 to 89.7) | 79.6 (64.4 to 94.7) | 70.9 (53.3 to 88.5) | 64.6 (44.1 to 85.1) | 62.5 (44.7 to 80.3) | 83.5 (76.1 to 90.8) | 85.5 (73.2 to 97.8) | 77 (58.6 to 95.3) | 78.4 (69.4 to 87.5) | 68 (51.2 to 84.7) | 74.0 (62.4 to 83.5) | 0.99 (0.98 to 1.01) |
|  | **White** | 66.7 (64.3 to 69) | 66.6 (64.3 to 68.9) | 67.9 (65.4 to 70.4) | 68.2 (66.2 to 70.2) | 69.2 (65.6 to 72.7) | 71.8 (68.7 to 74.9) | 70.7 (67.4 to 74) | 74.3 (71.5 to 77.1) | 72.7 (69.1 to 76.4) | 71.9 (70 to 73.9) | 71.3 (68.7 to 73.8) | 1.01 (1.0 to 1.01) |
| **Emergency C-Section** | **Asian** | 37.1 (32.3 to 41.9) | 33.9 (28.4 to 39.4) | 30.9 (23.3 to 38.6) | 33.5 (27.4 to 39.6) | 31.1 (25.6 to 36.6) | 32.2 (24.2 to 40.2) | 34.5 (27.6 to 41.4) | 43.9 (35.4 to 52.5) | 48  (41 to 55) | 40.9 (34.9 to 46.8) | 47.8 (42.3 to 53.2) | 1.04 (1.03 to 1.05) |
|  | **Black** | 30.8 (25.3 to 36.3) | 30.3 (24.2 to 36.4) | 33 (26.8 to 39.2) | 32.1 (25.3 to 38.8) | 37 (32.3 to 41.6) | 34.5 (27.6 to 41.5) | 42.5 (34.2 to 50.8) | 39.8 (31.9 to 47.8) | 43.8 (36.3 to 51.2) | 43.9 (39.8 to 48) | 53.0 (46.4 to 59.5) | 1.05 (1.04 to 1.07) |
|  | **Mixed** | 42.9 (26.6 to 59.3) | 31.8 (14.7 to 49) | 42.1 (23.6 to 60.5) | 43.3 (24 to 62.5) | 32.8 (16.2 to 49.4) | 22.5 (5.3 to 39.7) | 49.4 (30.7 to 68) | 45.3 (25.8 to 64.8) | 44.4 (21 to 67.8) | 37.7 (25.3 to 50.1) | 55.1 (40.2 to 69.3) | 1.03 (1.01 to 1.06) |
|  | **Other** | 23.7 (12 to 35.4) | 41.5 (20.6 to 62.4) | 31.4 (15.3 to 47.4) | 45.8 (26.7 to 64.8) | 38.9 (18.1 to 59.7) | 42.2 (24.3 to 60.1) | 35.6 (15.5 to 55.7) | 43.4 (22.8 to 63.9) | 45.8 (31.3 to 60.2) | 57.2 (45.3 to 69.1) | 53.5 (41.3 to 65.4) | 1.06 (1.03 to 1.08) |
|  | **White** | 37.2 (34.6 to 39.9) | 36  (33.1 to 39) | 39.2 (37 to 41.5) | 39.7 (37.1 to 42.4) | 37.6 (34.8 to 40.5) | 40 (36.9 to 43.2) | 41.4 (38.2 to 44.7) | 42.5 (38.9 to 46.1) | 49.9 (46 to 53.7) | 45.6 (43.3 to 48) | 50.8 (48.0 to 53.7) | 1.03 (1.03 to 1.04) |
| **Intubation in delivery room** | **Asian** | 91.2 (87.9 to 94.6) | 90.3 (86.4 to 94.3) | 90.2 (87 to 93.5) | 86.5 (81.6 to 91.5) | 85.8 (81.7 to 90) | 85.7 (82.9 to 88.4) | 76.6 (69.6 to 83.5) | 69.6 (63.4 to 75.7) | 68.9 (64 to 73.7) | 69.3 (65.7 to 72.8) | 66.1 (60.8 to 71.1) | 0.96 (0.96 to 097) |
|  | **Black** | 91.3 (88.3 to 94.4) | 90.8 (87 to 94.5) | 93.6 (90.7 to 96.5) | 90.4 (86.5 to 94.2) | 88.6 (84.4 to 92.9) | 86.8 (81.7 to 91.9) | 78.5 (72.2 to 84.7) | 83.4 (79.5 to 87.3) | 75.2 (67.3 to 83) | 74.8 (68.3 to 81.3) | 68.3 (62.1 to 74.1) | 0.97 (0.96 to 0.97) |
|  | **Mixed** | 86.9 (76.9 to 97) | 94.7 (87.6 to 101.8) | 78.8 (62.7 to 94.9) | 86.5 (74.9 to 98.1) | 87.4 (77.2 to 97.5) | 94.2 (85.7 to 102.7) | 75.2 (58 to 92.5) | 88.8 (80.4 to 97.2) | 63.5 (47.6 to 79.4) | 64 (52.5 to 75.4) | 67.3 (52.9 to 79.7) | 0.97 (0.96 to 0.98) |
|  | **Other** | 90.2 (81.7 to 98.6) | 95.4 (89.5 to 101.4) | 90.1 (80.2 to 99.9) | 87.7 (78.1 to 97.4) | 68.3 (52.4 to 84.2) | 73.6 (62.2 to 85) | 77.3 (64.3 to 90.2) | 76 (63.2 to 88.8) | 65.8 (59.5 to 72.2) | 63.8 (47.9 to 79.8) | 63.0 (50.9 to 74.0) | 0.96 (0.95 to 0.97) |
|  | **White** | 87.6 (85.7 to 89.5) | 87 (85.7 to 88.4) | 85.9 (84.2 to 87.7) | 82.6 (80.3 to 85) | 81.8 (80.2 to 83.4) | 78 (75.8 to 80.2) | 75.3 (71.6 to 79) | 72.6 (69.9 to 75.4) | 68.9 (66 to 71.9) | 66.9 (64.1 to 69.6) | 63.7 (61.0 to 66.4) | 0.96 (0.96 to 0.97) |
| **Transfer within first 48 hours (any direction)** | **Asian** | 20.9 (15.4 to 26.4) | 16.8 (13.1 to 20.5) | 20.3 (13.3 to 27.3) | 15.3 (9.3 to 21.3) | 13.5 (9.1 to 17.9) | 20.4 (15.8 to 25.0) | 18.6 (14.1 to 23.2) | 10.6 (5.2 to 16.0) | 11.3 (7.9 to 14.6) | 13.6 (9.2 to 18) | 13.3 (9.9 to 17.4) | 0.96 (0.94 to 0.99) |
|  | **Black** | 19.5 (14.6 to 24.5) | 17.2 (11.2 to 23.3) | 16.5 (11.4 to 21.6) | 17.6 (11.8 to 23.3) | 21.8 (15.5 to 28.0) | 19.2 (14.6 to 23.9) | 14.6 (9.4 to 19.9) | 19.8 (14.3 to 25.3) | 15.3 (8.5 to 22.2) | 15.6 (8.4 to 22.7) | 11.5 (7.8 to 16.2) | 0.99 (0.96 to 1.01) |
|  | **Mixed** | 15.4 (-3.5 to 34.4) | 20.8 (0.3 to 41.4) | 20.7 (6.8 to 34.6) | 12.4 (0.0 to 24.8) | 14 (1.4 to 26.5) | 10.9 (0.7 to 21.1) | 26.4 (13.4 to 39.3) | 9.3 (0.0 to 22.1) | 4.6 (0.0 to 10.5) | 17.1 (4.6 to 29.6) | 19.2 (9.6 to 32.5) | 1.02 (0.96 to 1.08) |
|  | **Other** | 14.7 (3.0 to 26.4) | 7.6 (0.0 to 15.4) | 9.4 (2.4 to 16.4) | 28.5 (8.7 to 48.2) | 23.3 (5.1 to 41.5) | 20.4 (4.2 to 36.5) | 13.2 (0.4 to 26) | 20.1 (0.9 to 39.4) | 17.5 (8.4 to 26.6) | 29.8 (18.1 to 41.5) | 13.7 (6.8 to 23.7) | 1.03 (0.97 to 1.08) |
|  | **White** | 21.3 (19.0 to 23.6) | 23.2 (20.8 to 25.5) | 22.5 (20.4 to 24.6) | 22.5 (20.6 to 24.5) | 22.0 (18.9 to 25.0) | 21.6 (19.6 to 23.5) | 20.5 (18.1 to 22.9) | 18.0 (15.1 to 20.9) | 20.6 (17.1 to 24.0) | 19.9 (18.3 to 21.4) | 20.1 (17.9 to 22.5) | 0.99 (0.98 to 0.99) |
| **Transfer within first 48 hours (upward)** | **Asian** | 17.9 (12.6 to 23.3) | 16.8 (13.1 to 20.5) | 19.2 (12.6 to 25.9) | 14.2 (8.9 to 19.5) | 12.2 (8.4 to 16.1) | 19.4 (14.5 to 24.3) | 15.2 (10.9 to 19.4) | 13.3 (8.6 to 17.9) | 10.9 (8.2 to 13.6) | 13.7 (10 to 17.4) | 11.6 (8.4 to 15.5) | 0.96 (0.94 to 0.99) |
|  | **Black** | 17.2 (12.5 to 21.9) | 17.2 (11.4 to 23.1) | 14.8 (10.1 to 19.6) | 15.6 (10.4 to 20.8) | 21.0 (14.9 to 27) | 18.9 (13.9 to 23.9) | 16.4 (12.1 to 20.8) | 19.9 (14.7 to 25.2) | 17.8 (11.2 to 24.4) | 17.9 (12.0 to 23.8) | 9.9 (6.4 to 14.3) | 0.99 (0.96 to 1.01) |
|  | **Mixed** | 56.7 (10.9 to 100.0) | 52.8 (6.3 to 99.3) | 41.4 (27.7 to 55.2) | 25.8 (20.4 to 31.2) | 28.4 (20.1 to 36.7) | 30.0 (15.1 to 44.9) | 41.4 (35.2 to 47.6) | 46.7 (20.5 to 72.8) | 25.0 (25.0 to 250) | 31.4 (16.2 to 46.5) | 19.2 (9.6 to 32.5) | 1.03 (0.96 to 1.09) |
|  | **Other** | 32.3 (18.0 to 46.7) | 27.8 (22.3 to 33.2) | 19.8 (15.5 to 24.1) | 50.7 (24.5 to 76.9) | 43.3 (19.0 to 67.7) | 21.7 (18.3 to 25.1) | 41.6 (11.4 to 71.8) | 60.4 (29.6 to 91.3) | 23.6 (13.6 to 33.7) | 35.3 (23.7 to 46.9) | 13.7 (6.8 to 23.7) | 1.02 (0.97 to 1.08) |
|  | **White** | 18.8 (16.8 to 20.7) | 20.5 (18.3 to 22.8) | 19.9 (18.1 to 21.7) | 20.1 (18.3 to 21.9) | 20.1 (17.2 to 23.0) | 19.1 (16.9 to 21.4) | 19.0 (16.4 to 21.6) | 17.4 (14.6 to 20.2) | 19.3 (15.9 to 22.7) | 19.0 (17.2 to 20.8) | 19.0 (16.9 to 21.3) | 0.99 (0.98 to 1.01) |
| **Transfer within first 48 hours (horizontal)** | **Asian** | 5.7  (4.5 to 6.9) | 0 | 4.2  (3.2 to 5.3) | 4.3  (3.8 to 4.8) | 7.0 (2.9 to 11.0) | 4.2 | 6.3  (3.3 to 9.2) | 3.9 | 5.6  (4.4 to 6.8) | 4.0 (3.0 to 4.9) | 1.5 (0.5 to 3.3) | 1.01 (0.91 to 1.11) |
|  | **Black** | 6.9  (5.2 to 8.7) | 9.1 (6.3 to 11.9) | 9.8 (8.4 to 11.2) | 3.7  (2.8 to 4.6) | 4.8  (3.3 to 6.3) | 4.2 | 5.6 | 4.0 | 5.6 | 5.9 | 1.2 (0.3 to 3.6) | 0.87 (0.75 to 1.02) |
|  | **Mixed** | 0 | 50.0 | 0 | 33.3 | 40.0 | 0 | 0 | 0 | 0 | 0 | 0 | 0.85 (0.68 to 1.06) |
|  | **Other** | 0 | 0 | 33.3 | 25.0 | 20.0 | 100.0 | 33.3 | 0 | 16.7 | 22.5 (17.6 to 27.4) | 0 | 1.02 (0.87 to 1.19) |
|  | **White** | 2.0  (1.3 to 2.7) | 2.5  (1.6 to 3.5) | 2.2  (1.6 to 2.8) | 2.6  (2.2 to 3.0) | 2.2  (1.4 to 2.9) | 2.3  (1.6 to 3.1) | 1.9  (1.4 to 2.4) | 1.5  (1.1 to 1.9) | 1.8  (1.1 to 2.4) | 1.6 (0.9 to 2.2) | 0.6 (0.2 to 1.1) | 0.89 (0.86 to 0.93) |
| **Transfer within first 48 hours (downward)** | **Asian** | 4.8 (2.8 to 6.7) | 0 | 0 | 9.1 | 0 | 0 | 7.9 (5.5 to 10.3) | 3.9 | 0 | 4.2 (3.5 to 4.9) | 0 | 0.95 (0.77 to 1.17) |
|  | **Black** | 6.8  (5.0 to 8.6) | 10.5 | 0 | 7.9 (0.0 to 17.0) | 0 | 4.0 | 0 | 6.7 | 0 | 4.9 (3.1 to 6.8) | 0 | 0.91 (0.73 to 1.14) |
|  | **Mixed** | 100.0 | 0 | 0 | 10.0 | 0 | 0 | 0 | 0 | 0 | 0 | 0 | 0.67 (0.33 to 1.33) |
|  | **Other** | 16.7 | 0 | 0 | 0 | 0 | 0 | 0 | 0 | 0 | 0 | 0 | Not converged |
|  | **White** | 1.2  (0.8 to 1.5) | 1.4  (0.7 to 2.0) | 0.8  (0.8 to 0.9) | 0.9  (0.9 to 1.0) | 2.0  (2.0 to 2.1) | 1.0  (0.8 to 1.2) | 1.0 | 0 | 0.9 | 1.6 | 0.2 (0 to 0.6) | 0.76 (0.64 to 0.88) |
| **Intubated respiratory support** | **Asian** | 97 (95.7 to 98.4) | 97 (94.9 to 99.2) | 97.8 (96.4 to 99.1) | 93.2 (90.4 to 96.1) | 93 (90.6 to 95.4) | 95.5 (93.3 to 97.7) | 93.3 (89.9 to 96.8) | 88.2 (83.9 to 92.4) | 87.6 (83.3 to 92) | 90.1 (86.6 to 93.6) | 87.1 (83.1 to 90.5) | 0.99 (0.99 to 0.99) |
|  | **Black** | 96.5 (94.3 to 98.7) | 96.9 (95.3 to 98.5) | 97.1 (94.8 to 99.3) | 96.3 (94.5 to 98.1) | 97.9 (95.6 to 100.2) | 96.2 (93.4 to 99.1) | 93.5 (90.6 to 96.5) | 95.1 (92.7 to 97.5) | 90 (84.4 to 95.6) | 92.6 (88.4 to 96.8) | 89.7 (85.1 to 93.2) | 0.99 (0.99 to 0.99) |
|  | **Mixed** | 95.8 (90 to 101.7) | 97 (91 to 102.9) | 85.6 (69 to 102.1) | 95.9 (90.5 to 101.3) | 95.1 (88.6 to 101.6) | 95.8 (87.7 to 104) | 95.4 (89.1 to 101.8) | 100 (100 to 100) | 87.8 (77.8 to 97.8) | 89.9 (83.4 to 96.3) | 80.8 (67.5 to 90.4) | 0.99 (0.99 to 0.99) |
|  | **Other** | 93.2 (85.9 to 100.4) | 100 (100 to 100) | 95.8 (87.7 to 104) | 97.2 (91.8 to 102.7) | 90.3 (74 to 106.6) | 90.3 (78.2 to 102.4) | 88.4 (77.3 to 99.5) | 100 (100 to 100) | 93  (88 to 97.9) | 90.4 (83.4 to 97.4) | 84.7 (74.3 to 92.1) | 0.98 (0.98 to 0.99) |
|  | **White** | 97.7 (97.1 to 98.2) | 96.7 (95.6 to 97.7) | 95.9 (94.9 to 97) | 95.6 (94.7 to 96.5) | 95.9 (94.7 to 97) | 94.6 (93.6 to 95.7) | 92.9 (92 to 93.8) | 92.6 (91.3 to 93.9) | 92 (90.4 to 93.6) | 90.6 (88.6 to 92.5) | 89.9 (88.1 to 91.6) | 0.99 (0.99 to 0.99) |
| **PDA closure** | **Asian** | 3.8  (1.7 to 6.0) | 2.6  (0.8 to 4.4) | 5.1  (2.5 to 7.7) | 3.0  (0.5 to 5.6) | 4.6  (2.6 to 6.6) | 1.9  (0.0 to 4) | 2.8  (0.7 to 4.9) | 1.4  (0.0 to 2.9) | 1.4  (0.0 to 3) | 0.3 (0.0 to 1.0) | 0.6 (0 to 2.1) | 0.84 (0.79 to 0.89) |
|  | **Black** | 7.0  (4.9 to 9.0) | 4.1  (2.0 to 6.1) | 4.8  (1.7 to 7.8) | 3.8  (1.2 to 6.3) | 2.3  (0.9 to 3.7) | 3.9  (1.0 to 6.8) | 0.9  (0.0 to 2.1) | 1.3  (0.0 to 2.7) | 1.3  (0.0 to 2.6) | 1.1 (0.0 to 2.1) | 0.8 (0.1 to 3.0) | 0.81 (0.75 to 0.88) |
|  | **Mixed** | 5.6 (0.0 to 13.9) | 9.1  (0.0 to 19) | 3.3  (0.0 to 7.8) | 1.3  (0.0 to 3.8) | 4.2 (0.0 to 12.3) | 4.2 (0.0 to 12.3) | 0  (0 to 0) | 0  (0 to 0) | 1.7  (0.0 to 4.9) | 1.4 (0.0 to 4.1) | 0 | 0.81 (0.66 to 0.99) |
|  | **Other** | 8.3 (0.0 to 17.9) | 8.9 (1.3 to 16.5) | 20.1 (0.0 to 41.7) | 7.7  (0.3 to 15) | 0  (0 to 0) | 0  (0 to 0) | 7.2 (0.0 to 15.9) | 0 (0 to 0) | 1.7  (0.0 to 4.9) | 0  (0 to 0) | 0 | 0.74 (0.64 to 0.85) |
|  | **White** | 5.8  (4.6 to 7.1) | 4.2  (3.5 to 4.9) | 4.2  (3 to 5.3) | 3.5  (2.5 to 4.4) | 3  (2.1 to 3.9) | 3  (1.9 to 4.2) | 2.1  (1.4 to 2.9) | 1.7  (0.8 to 2.6) | 1.6  (0.8 to 2.3) | 0.6 (0.1 to 1.1) | 0.9 (0.4 to 1.6) | 0.84 (0.81 to 0.86) |
| **Any own mother’s milk at discharge** | **Asian** | 46.7 (39.9 to 53.5) | 42.8 (37.0 to 48.5) | 46.6 (40.9 to 52.4) | 42.5 (36.3 to 48.6) | 44.8 (37.1 to 52.4) | 49.6 (44.3 to 54.9) | 43 (34.6 to 51.3) | 58.8 (51.9 to 65.7) | 47.9 (41.3 to 54.5) | 45.7 (40.5 to 50.9) | 58.2 (51.8 to 64.3) | 1.01 (0.99 to 1.01) |
|  | **Black** | 55.5 (51.1 to 59.9) | 52.6 (46.7 to 58.6) | 56.5 (48.7 to 64.3) | 51.6 (46.4 to 56.8) | 48.7 (40.8 to 56.6) | 51.1 (44.6 to 57.6) | 52.6 (40.8 to 64.5) | 50.5 (43.3 to 57.7) | 48.4 (41.5 to 55.3) | 51.4 (43.8 to 59.1) | 71.4 (64.4 to 77.8) | 1.0 (0.99 to 1.01) |
|  | **Mixed** | 38.3 (21.8 to 54.8) | 50 (29.7 to 70.3) | 38.9 (21.2 to 56.6) | 33.5 (14.2 to 52.8) | 41.2 (24.1 to 58.4) | 58.6 (37.3 to 79.9) | 28.4 (13.7 to 43) | 34.2 (16.3 to 52.1) | 39.6 (25.3 to 53.9) | 49.6 (33.1 to 66.2) | 63.9 (46.2 to 79.2) | 1.04 (1.01 to 1.06) |
|  | **Other** | 45.5 (33.5 to 57.5) | 52.4 (37.1 to 67.8) | 54.4 (37.5 to 71.4) | 70.3 (58.3 to 82.3) | 56.4 (38.1 to 74.7) | 61.6 (43.1 to 80.0) | 40.9 (25.1 to 56.7) | 40.9 (23.7 to 58) | 42.5 (26.6 to 58.5) | 47.7 (30.9 to 64.5) | 67.8 (54.4 to 79.4) | 0.99 (0.98 to 1.01) |
|  | **White** | 33.3 (30.6 to 36.0) | 33.0 (29.9 to 36.1) | 32.1 (29.6 to 34.6) | 35.3 (32.4 to 38.2) | 34.9 (32.3 to 37.4) | 35.9 (32.6 to 39.3) | 37.3 (34.6 to 40.0) | 37.6 (34.6 to 40.7) | 36.8 (33.9 to 39.7) | 35 (33.6 to 36.3) | 49.0 (45.8 to 52.2) | 1.02 (1.01 to 1.02) |

**Supplementary Table 6B**

**Very preterm (VPT) admissions (28-<32w gestation), care processes by maternal ethnicity and year**

Adjusted risk ratio and 95% confidence interval (aRR; 95% CI) estimated by modified Poisson regression; adjustment variables are sex, gestational age (weeks), birth weight z-score, and multiplicity; a missing confidence interval indicates that only one individual with that outcome was recorded; this analysis includes 46325 VPT admissions; 9464 VPT admissions were excluded because of missing ethnicity data

|  | **Maternal Ethnicity** | **2013**  **% (95% CI)** | **2014**  **% (95% CI)** | **2015**  **% (95% CI)** | **2016**  **% (95% CI)** | **2017**  **% (95% CI)** | **2018**  **% (95% CI)** | **2019**  **% (95% CI)** | **2020**  **% (95% CI)** | **2021**  **% (95% CI)** | **2022**  **% (95% CI)** | **2023**  **% (95% CI)** | **Overall trend, aRR (95% CI)** |
| --- | --- | --- | --- | --- | --- | --- | --- | --- | --- | --- | --- | --- | --- |
| **Antenatal steroids** | **Asian** | 88.1 (85.6 to 90.6) | 90 (88.5 to 91.4) | 91.8 (89.8 to 93.8) | 90 (87.4 to 92.6) | 90.7 (88.4 to 93) | 94.2 (92.7 to 95.8) | 94.9 (93.4 to 96.3) | 91.1 (87.9 to 94.2) | 95.4 (93.8 to 96.9) | 89.7 (86.8 to 92.6) | 92.2 (89.9 to 94.2) | 1.0 (1.0 to 1.0) |
|  | **Black** | 92.1 (90.0 to 94.2) | 92.4 (90.3 to 94.4) | 89.6 (85.2 to 94.1) | 93.7 (92.2 to 95.2) | 87.7 (84.5 to 91.) | 92.5 (88.3 to 96.7) | 93.7 (90.2 to 97.1) | 93.1 (88.8 to 97.5) | 90.1 (87.9 to 92.4) | 92.2 (88.8 to 95.7) | 94.3 (91.5 to 96.5) | 1.0 (1.0 to 1.0) |
|  | **Mixed** | 91.1 (85.5 to 96.7) | 85.8 (76.3 to 95.3) | 89.7 (80.8 to 98.6) | 90.8 (84.5 to 97.0) | 87.9 (78.0 to 97.9) | 90.6 (80.7 to 100.4) | 92.6 (88.1 to 97.1) | 93.1 (85.3 to 100.9) | 89.2 (80.5 to 98.0) | 88.8 (82.0 to 95.6) | 88.2 (79.8 to 93.9) | 1.0 (0.99 to 1.0) |
|  | **Other** | 90.2 (84.5 to 96.0) | 86.5 (77.5 to 95.4) | 91.7 (85.3 to 98.0) | 88.8 (81.6 to 95.9) | 92.4 (85.2 to 99.6) | 97.5 (94.2 to 100.8) | 91.5 (86.5 to 96.5) | 94.2 (87.8 to 100.5) | 94.8 (88.5 to 101.2) | 91.8 (84.4 to 99.2) | 93.3 (87.3 to 97.1) | 1.0 (1.0 to 1.0) |
|  | **White** | 90.0 (88.8 to 91.2) | 89.8 (89.1 to 90.6) | 91 (89.7 to 92.3) | 90 (89 to 91.1) | 91.8 (90.8 to 92.8) | 91.8 (91.1 to 92.6) | 92.4 (91.7 to 93.1) | 93.7 (93.0 to 94.4) | 92.6 (91.5 to 93.8) | 92.7 (91.5 to 93.9) | 91.2 (90.1 to 92.3) | 1.0 (1.0 to 1.0) |
| **Birth in a hospital with a neonatal intensive care unit** | **Asian** | 50.7 (45 to 56.3) | 54.3 (51.9 to 56.7) | 54.4 (48.1 to 60.7) | 56.6 (50.9 to 62.4) | 54.2 (48.9 to 59.5) | 53.3 (49 to 57.6) | 55.8 (52.3 to 59.2) | 52.5 (48.7 to 56.4) | 56.9 (53.3 to 60.4) | 54.5 (49.1 to 60.0) | 55.2 (51.2 to 59.0) | 1.01 (1.0 to 1.01) |
|  | **Black** | 48.9 (43.8 to 53.9) | 53.1 (48.6 to 57.7) | 59.9 (56.6 to 63.1) | 55 (47.5 to 62.5) | 53.2 (48.2 to 58.3) | 50.1 (41.9 to 58.2) | 52.5 (43.9 to 61) | 59.9 (53.2 to 66.5) | 66.5 (60.3 to 72.7) | 54.6 (48.6 to 60.7) | 55.5 (50.3 to 60.6) | 1.01 (1.0 to 1.02) |
|  | **Mixed** | 62.7 (50.2 to 75.1) | 46.1 (32.5 to 59.6) | 50.4 (43.5 to 57.3) | 62.6 (46.3 to 78.9) | 53.3 (40.2 to 66.4) | 60.9 (44.6 to 77.2) | 69.7 (56 to 83.3) | 48 (30.6 to 65.5) | 41.6 (27.9 to 55.2) | 54.2 (43.4 to 65) | 54.8 (44.2 to 65.2) | 0.99 (0.98 to 1.01) |
|  | **Other** | 47.8 (37.2 to 58.4) | 43.9 (31 to 56.8) | 55.5 (40.7 to 70.4) | 51.2 (39.4 to 63) | 58.3 (43.2 to 73.3) | 56.8 (46.4 to 67.3) | 65.1 (54.5 to 75.7) | 63.6 (55 to 72.3) | 48 (40.5 to 55.4) | 51.7 (38.4 to 65) | 61.1 (51.9 to 69.9) | 1.02 (1.0 to 1.03) |
|  | **White** | 45.4 (43.4 to 47.4) | 48 (46.1 to 49.9) | 48.5 (46.1 to 51) | 48 (45.5 to 50.4) | 49.5 (47.5 to 51.4) | 49.5 (48.2 to 50.9) | 48 (46.5 to 49.5) | 49.6 (48 to 51.3) | 51 (49.6 to 52.4) | 47.3 (45.8 to 48.9) | 47.1 (45.2 to 49.0) | 1.0 (1.0 to 1.01) |
| **Emergency C-Section** | **Asian** | 55 (50.6 to 59.4) | 56.8 (51.1 to 62.5) | 62.8 (58.1 to 67.5) | 56.6 (53.9 to 59.3) | 60.3 (54.9 to 65.7) | 58 (52.6 to 63.5) | 64.9 (60.1 to 69.7) | 62 (57.5 to 66.5) | 60.1 (56.4 to 63.9) | 67.8 (63.2 to 72.4) | 67.8 (63.9 to 71.4) | 1.02 (1.01 to 1.02) |
|  | **Black** | 62.5 (58.4 to 66.7) | 65.7 (61.6 to 69.8) | 58.2 (53.4 to 63) | 60.2 (53.3 to 67.1) | 66.9 (62.6 to 71.3) | 65.7 (60 to 71.4) | 67.2 (60.8 to 73.6) | 63.9 (58.3 to 69.5) | 61.9 (56.1 to 67.7) | 69.2 (62.1 to 76.3) | 70.0 (65.0 to 74.7) | 1.01 (1.01 to 1.02) |
|  | **Mixed** | 61.1 (46.7 to 75.5) | 58.9 (41.1 to 76.7) | 45.1 (35.5 to 54.7) | 57.7 (45.4 to 69.9) | 55.6 (45 to 66.2) | 51.2 (36.1 to 66.3) | 54.3 (42.5 to 66.1) | 58.1 (40.6 to 75.6) | 54.6 (41.6 to 67.6) | 64.9 (55 to 74.8) | 74.7 (64.5 to 83.2) | 1.03 (1.02 to 1.04) |
|  | **Other** | 47.8 (32.8 to 62.9) | 52.3 (38.2 to 66.4) | 55.6 (40.5 to 70.8) | 50.3 (39.5 to 61.1) | 65.2 (52.7 to 77.8) | 44.3 (30.6 to 57.9) | 57.8 (48.2 to 67.4) | 58.6 (45 to 72.2) | 70 (59.4 to 80.7) | 67.9 (57.9 to 78) | 71.7 (62.4 to 79.8) | 1.03 (1.02 to 1.04) |
|  | **White** | 52.9 (51.5 to 54.3) | 56.1 (53.1 to 59) | 54 (51.7 to 56.4) | 54.7 (52.3 to 57.2) | 55.5 (53.6 to 57.4) | 57.2 (55 to 59.5) | 55.1 (52.8 to 57.3) | 57.9 (55.9 to 59.8) | 59.4 (57.4 to 61.3) | 61.2 (58.6 to 63.7) | 66.9 (65.1 to 68.7) | 1.02 (1.02 to 1.02) |
| **Intubation in delivery room** | **Asian** | 35.2 (32.7 to 37.6) | 31.2 (27.2 to 35.3) | 32.9 (30.1 to 35.6) | 34 (29.6 to 38.5) | 31.6 (27 to 36.2) | 29.9 (26.3 to 33.4) | 27.9 (24.4 to 31.4) | 23 (20.4 to 25.6) | 25.9 (22.8 to 29) | 16.2 (11.6 to 20.9) | 18.9 (15.9 to 22.1) | 0.94 (0.93 to 0.95) |
|  | **Black** | 42.1 (36 to 48.1) | 47.1 (42.2 to 51.9) | 46.2 (41.8 to 50.6) | 45.6 (40.2 to 51.1) | 39.4 (33 to 45.7) | 33.5 (27.6 to 39.4) | 36.9 (31.7 to 42.2) | 33.4 (26.7 to 40.1) | 20.9 (15.5 to 26.2) | 22.4 (17.4 to 27.4) | 18.2 (14.4 to 22.5) | 0.92 (0.91 to 0.93) |
|  | **Mixed** | 36.8 (24.1 to 49.5) | 28.8 (14.9 to 42.7) | 45.5 (39.4 to 51.5) | 41.5 (27.9 to 55.1) | 40.5 (31.5 to 49.5) | 30.2 (19.3 to 41.2) | 27.2 (18.1 to 36.3) | 30.5 (21.4 to 39.6) | 27 (15.5 to 38.6) | 25.7 (16 to 35.4) | 20.4 (12.8 to 30.0) | 0.94 (0.91 to 0.96) |
|  | **Other** | 39.1 (33.0 to 45.3) | 28.3 (14.9 to 41.6) | 30.2 (16 to 44.4) | 34.3 (26.5 to 42.1) | 44.5 (28.2 to 60.8) | 23.1 (13.9 to 32.3) | 23.5 (12.8 to 34.2) | 21.7 (13.1 to 30.2) | 21.7 (13.3 to 30.2) | 12.0 (6.0 to 18.0) | 20.7 (13.8 to 29.0) | 0.93 (0.91 to 0.95) |
|  | **White** | 35.6 (34.3 to 37) | 32.5 (31.2 to 33.8) | 32.4 (30.5 to 34.2) | 31.1 (29.8 to 32.4) | 29.3 (27.6 to 30.9) | 26.9 (25.3 to 28.5) | 25.1 (22.5 to 27.6) | 23.5 (21.7 to 25.4) | 22.1 (20.3 to 24) | 17.8 (16 to 19.6) | 17.0 (15.6 to 18.5) | 0.93 (0.92 to 0.94) |
| **Transfer within first 48 hours (any direction)** | **Asian** | 6.6 (4.6 to 8.6) | 8.6 (6.3 to 10.9) | 11.8 (7.2 to 16.4) | 8.3 (6.6 to 10.0) | 6.4 (4.5 to 8.4) | 6.3  (4.1 to 8.5) | 6.4 (3.7 to 9.2) | 7.0 (3.3 to 10.8) | 8.9 (6.4 to 11.5) | 5.7 (3.7 to 7.7) | 7.4 (5.5 to 9.7) | 0.98 (0.95 to 1.0) |
|  | **Black** | 6.8 (4.7 to 8.9) | 5.1 (3.2 to 7.0) | 9.6 (6.2 to 13.1) | 6.1 (4.0 to 8.3) | 6.8 (5.0 to 8.5) | 9.6 (6.2 to 13.0) | 7.3 (3.4 to 11.3) | 4.8  (2.5 to 7.0) | 4.8  (2.8 to 6.8) | 6.2 (3.1 to 9.3) | 8.6 (5.9 to 11.9) | 0.99 (0.96 to 1.04) |
|  | **Mixed** | 20.6 (4.9 to 36.3) | 8.2 (0.0 to 17.4) | 5.2 (1.4 to 9.0) | 8.5 (2.0 to 15.1) | 9.6 (3.0 to 16.2) | 10.3 (3.4 to 17.2) | 5.0 (0.0 to 10.4) | 6.0 (0.0 to 14.3) | 1.8 (0.0 to 4.2) | 9.1 (2.7 to 15.5) | 9.7 (4.5 to 17.6) | 0.95 (0.88 to 1.02) |
|  | **Other** | 5.7 (1.7 to 9.8) | 13.6 (4.1 to 23.0) | 4.2 (0.5 to 7.8) | 10.2 (4.5 to 16.0) | 20.3 (3.9 to 36.6) | 13.0 (5.4 to 20.7) | 8.7 (4.6 to 12.8) | 6.1 (1.5 to 10.8) | 4.7 (0.0 to 10.9) | 7.5 (2.6 to 12.4) | 11.6 (6.5 to 18.7) | 1.02 (0.96 to 1.08) |
|  | **White** | 10.3 (9.4 to 11.3) | 10.7 (9.9 to 11.6) | 11.9 (10.8 to 13.0) | 11.4 (10.4 to 12.4) | 11.3 (10.2 to 12.4) | 11.2 (9.8 to 12.7) | 12.0 (10.7 to 13.3) | 10.3 (9.0 to 11.7) | 12.0 (10.4 to 13.6) | 11.9 (10.6 to 13.2) | 11.3 (10.1 to 12.5) | 1.01 (1.0 to 1.02) |
| **Transfer within first 48 hours (upward)** | **Asian** | 4.3 (2.4 to 6.1) | 5.8 (4.3 to 7.2) | 7.7 (4.0 to 11.3) | 5.4 (3.4 to 7.4) | 4.0 (2.7 to 5.4) | 5.3  (3.8 to 6.9) | 3.7 (2.8 to 4.5) | 5.2  (3.2 to 7.1) | 4.2  (3.2 to 5.2) | 5.3  (3 to 7.5) | 5.1 (3.5 to 7.1) | 0.98 (0.95 to 1.02) |
|  | **Black** | 5.1 (3.7 to 6.6) | 4.6 (2.9 to 6.3) | 6.7 (4.3 to 9.0) | 5.4 (3.8 to 6.9) | 6.0 (4.2 to 7.8) | 6.1  (3.6 to 8.6) | 7.5 (2.7 to 12.3) | 5.1  (4.2 to 6.0) | 4.3  (3.3 to 5.3) | 5.7 (4.1 to 7.3) | 6.7 (4.4 to 9.7) | 1.04 (0.99 to 1.08) |
|  | **Mixed** | 12.6 (10.8 to 14.4) | 50.0 | 12.9 (7.6 to 18.1) | 18.2 (10.5 to 26.0) | 10.0 (7.3 to 12.6) | 17.3 (5.9 to 28.7) | 28.6 | 24.2 (0.0 to 49.5) | 9.1 | 20.3 (13.0 to 27.6) | 5.4 (1.8 to 12.1) | 1.07 (0.96 to 1.18) |
|  | **Other** | 13.4 (10.2 to 16.7) | 21.4 (13.2 to 29.6) | 12.5 (9.0 to 16.0) | 19.1 (12.3 to 25.9) | 32.7 (6.1 to 59.4) | 50.0 | 13.0 (8.1 to 17.9) | 14.1 (7.9 to 20.4) | 14.7 (4.6 to 24.8) | 11.0 (6.9 to 15.1) | 9.9 (5.2 to 16.7) | 1.03 (0.94 to 1.12) |
|  | **White** | 7.3 (6.7 to 8.0) | 7.1 (6.3 to 7.9) | 8.0 (7.1 to 8.9) | 7.4 (6.6 to 8.2) | 7.7 (6.6 to 8.7) | 7.5  (6.1 to 8.9) | 8.5 (7.6 to 9.4) | 7.2  (6.5 to 7.9) | 8.6  (7.5 to 9.8) | 8.8 (7.6 to 10) | 8.8 (7.7 to 9.9) | 1.02 (1.01 to 1.03) |
| **Transfer within first 48 hours (horizontal)** | **Asian** | 2.5 (1.6 to 3.3) | 2.2 (1.5 to 2.9) | 2.4 (1.5 to 3.2) | 3.6 (2.2 to 5.1) | 3.2 (2.2 to 4.2) | 4.0  (1.0 to 7.0) | 3.4 (1.3 to 5.4) | 2.7  (1.4 to 4.0) | 4.5  (2.7 to 6.3) | 1.9 | 0.9 (0.3 to 2.0) | 0.97 (0.90 to 1.03) |
|  | **Black** | 4.5 (1.8 to 7.2) | 4.9 (2.7 to 7.2) | 4.1 (3.1 to 5.1) | 3.8 (3.4 to 4.2) | 3.6 (2.7 to 4.4) | 5.0  (3.6 to 6.4) | 10.0 | 0 | 4.9  (1.8 to 7.9) | 4.2 (2.9 to 5.6) | 1.1 (0.3 to 2.7) | 0.94 (0.85 to 1.04) |
|  | **Mixed** | 25.0 | 0 | 12.5 | 21.9 (10.5 to 33.3) | 28.6 | 12.8 (8.5 to 17.0) | 0 | 0 | 0 | 7.7 | 3.2 (0.7 to 9.1) | 0.95 (0.80 to 1.12) |
|  | **Other** | 0 | 15.6 (6.9 to 24.3) | 0 | 11.8 (9.3 to 14.3) | 16.7 | 14.0 (8.9 to 19.2) | 11.1 | 0 | 12.5 | 17.5 (2.8 to 32.2) | 0 | 0.99 (0.86 to 1.13) |
|  | **White** | 1.5 (1.0 to 1.9) | 1.7 (1.3 to 2.1) | 2.0 (1.4 to 2.5) | 1.9 (1.3 to 2.4) | 1.8 (1.3 to 2.3) | 1.9  (1.6 to 2.2) | 1.6 (0.9 to 2.4) | 1.4  (0.9 to 1.9) | 1.4  (0.8 to 2.0) | 1.4 (1.0 to 1.8) | 1.5 (1.0 to 2.0) | 0.98 (0.96 to 1.01) |
| **Transfer within first 48 hours (downward)** | **Asian** | 3.2 (1.9 to 4.5) | 3.8 (2.2 to 5.3) | 6.6 (3.6 to 9.6) | 2.8 (2.3 to 3.3) | 4.2 (2.3 to 6.1) | 2.9  (1.5 to 4.4) | 4.8 (2.7 to 6.9) | 4.6  (2.6 to 6.5) | 4.1  (2.9 to 5.3) | 4.0 (1.1 to 6.9) | 0.9 (0.3 to 2.0) | 0.93 (0.88 to 0.98) |
|  | **Black** | 5.0 (1.4 to 8.6) | 2.5 (2.4 to 2.7) | 6.1 (1.1 to 11.1) | 5.0 (2.2 to 7.8) | 4.0 (3.4 to 4.6) | 7.3 (4.6 to 10.0) | 5.6 (3.7 to 7.4) | 5.9 (0.0 to 12.2) | 4.4 (2.1 to 6.6) | 5.5 (4.1 to 7.0) | 1.1 (0.3 to 2.7) | 0.99 (0.92 to 1.06) |
|  | **Mixed** | 36.9 (5.2 to 68.5) | 24.3 (15.9 to 32.7) | 11.1 | 0 | 11.8 (9.6 to 13.9) | 33.3 | 16.7 | 0 | 0 | 0 | 1.1 (0 to 5.8) | 0.69 (0.55 to 0.86) |
|  | **Other** | 14.3 (14.3 to 14.3) | 25.0 (25.0 to 25.0) | 0 | 10.6 (9.5 to 11.7) | 13.3 (6.8 to 19.9) | 10.7 (6.4 to 15.0) | 13.7 (7.8 to 19.5) | 15.6 (6.9 to 24.3) | 0 | 11.1 | 0 | 0.94 (0.85 to 1.04) |
|  | **White** | 1.6 (1.1 to 2.0) | 1.8 (1.1 to 2.4) | 1.8 (1.2 to 2.5) | 2.3 (1.7 to 2.8) | 1.8 (1.4 to 2.1) | 1.6  (1.2 to 2.0) | 1.5 (1.0 to 2.1) | 1.8  (1.2 to 2.3) | 2.0  (1.0 to 3.1) | 1.6 (1.1 to 2.1) | 0.8 (0.5 to 1.2) | 0.97 (0.95 to 0.99) |
| **Intubated respiratory support** | **Asian** | 50.8 (48.4 to 53.2) | 44.6 (40.4 to 48.8) | 47 (43.3 to 50.8) | 48.7 (45 to 52.3) | 49 (44 to 54.1) | 46.8 (42.7 to 50.9) | 44.5 (40.7 to 48.2) | 40.7 (36.6 to 44.9) | 41.3 (36.9 to 45.7) | 31.5 (26.7 to 36.2) | 36.7 (33.0 to 40.6) | 0.97 (0.96 to 0.97) |
|  | **Black** | 53.1 (49.4 to 56.9) | 55.8 (50.3 to 61.3) | 59.1 (53.5 to 64.7) | 58.2 (52.2 to 64.2) | 55.4 (50.4 to 60.5) | 49.3 (42.2 to 56.5) | 53.5 (45.9 to 61.1) | 47.5 (41.8 to 53.2) | 38.1 (34 to 42.3) | 34.6 (28.1 to 41) | 34.6 (29.7 to 39.7) | 0.96 (0.95 to 0.96) |
|  | **Mixed** | 52.6 (41.2 to 64.1) | 40.5 (25.8 to 55.1) | 61.0 (52.2 to 69.9) | 51.9 (37.2 to 66.5) | 53.5 (45.5 to 61.5) | 51.1 (40.2 to 62.1) | 39.3 (30.0 to 48.6) | 45.7 (33.0 to 58.5) | 42.6 (31.0 to 54.2) | 50.4 (39.9 to 61.0) | 38.7 (28.8 to 49.4) | 0.97 (0.95 to 0.98) |
|  | **Other** | 54.5 (47.1 to 61.8) | 54.1 (40.9 to 67.2) | 54.5 (43.8 to 65.1) | 53.7 (41.5 to 65.9) | 54.8 (38.0 to 71.6) | 52.1 (42.6 to 61.7) | 37.4 (25 to 49.9) | 33.2 (23.9 to 42.6) | 37.7 (28.9 to 46.5) | 28.6 (19.4 to 37.8) | 46.2 (37.0 to 55.6) | 0.95 (0.94 to 0.97) |
|  | **White** | 55.1 (54.1 to 56.2) | 53.4 (51.8 to 54.9) | 52.8 (51.6 to 54) | 52 (50.8 to 53.3) | 51.4 (50.1 to 52.8) | 49.7 (47.7 to 51.7) | 46.9 (44.3 to 49.6) | 46.2 (44.5 to 47.9) | 44.2 (41.4 to 47.0) | 40.4 (38.5 to 42.2) | 41.1 (39.2 to 42.9) | 0.97 (0.97 to 0.97) |
| **PDA closure** | **Asian** | 0.3 (0.0 to 0.6) | 0.1 (0.0 to 0.4) | 0  (0 to 0) | 0.2 (0.0 to 0.5) | 0.5  (0 to 1.1) | 0.5  (0.0 to 1.1) | 0 | 0 | 0.4  (0.0 to 0.8) | 0 | 0 | 0.90 (0.77 to 1.06) |
|  | **Black** | 0.4 (0.0 to 1.0) | 0 | 0 | 0.2 (0.0 to 0.7) | 0.5 (0.0 to 1.2) | 0 | 0 | 0 | 0.3  (0.0 to 0.8) | 0 | 0 | 0.86 (0.64 to 1.14) |
|  | **Mixed** | 0  (0 to 0) | 0 | 0 | 2.1 (0.0 to 4.9) | 0 | 0 | 0 | 0 | 0 | 0 | 0 | Not converged |
|  | **Other** | 0 | 0 | 0 | 0 | 0 | 0 | 0 | 0 | 0 | 0 | 0 | Not converged |
|  | **White** | 0.3 (0.1 to 0.5) | 0.2  (0 to 0.4) | 0.2 (0.1 to 0.4) | 0.3 (0.2 to 0.4) | 0.2 (0.0 to 0.3) | 0.1  (0 to 0.2) | 0.1 (0.0 to 0.2) | 0.2  (0.0 to 0.3) | 0.1  (0.0 to 0.2) | 0 | 0 | 0.84 (0.77 to 0.92) |
| **Any own mother’s milk at discharge** | **Asian** | 68.2 (65.3 to 71) | 72.1 (68.5 to 75.7) | 68.8 (65.9 to 71.6) | 72.1 (67.9 to 76.3) | 70.8 (64.7 to 76.8) | 69.8 (64.8 to 74.7) | 71.4 (68 to 74.8) | 68.7 (63.7 to 73.8) | 67.5 (61.9 to 73.2) | 73.8 (70.3 to 77.2) | 75.7 (72.1 to 79.0) | 1.0 (0.99 to 1.0) |
|  | **Black** | 78.7 (73.3 to 84) | 73.8 (69.9 to 77.7) | 74.5 (70.4 to 78.6) | 71.7 (65 to 78.5) | 73.9 (67.3 to 80.5) | 72.7 (68.7 to 76.6) | 77.2 (73.3 to 81.2) | 75.9 (71.4 to 80.4) | 71.3 (64.6 to 78.1) | 74.5 (68.5 to 80.5) | 82.5 (78.2 to 86.3) | 1.0 (1.0 to 1.0) |
|  | **Mixed** | 60 (51.6 to 68.4) | 74.7 (62.1 to 87.3) | 54.6 (41.9 to 67.3) | 62.6 (44.9 to 80.3) | 60.5 (47.9 to 73.0) | 52.3 (37.7 to 66.9) | 73.4 (65.3 to 81.6) | 63.6 (48.5 to 78.6) | 71.9 (62.8 to 81.0) | 57.1 (44.4 to 69.9) | 61.3 (50.6 to 71.2) | 1.0 (0.99 to 1.02) |
|  | **Other** | 74.6 (66.0 to 83.2) | 68.2 (59.0 to 77.3) | 72.9 (62.3 to 83.5) | 74.7 (65.4 to 84.0) | 73.2 (58.3 to 88.1) | 79.9 (72.9 to 86.9) | 70.1 (60.4 to 79.9) | 88.1 (81.3 to 95.0) | 80.8 (73.3 to 88.3) | 77.6 (69.1 to 86.2) | 73.6 (64.1 to 81.7) | 1.0 (0.99 to 1.01) |
|  | **White** | 52.3 (50.9 to 53.7) | 50.9 (48.6 to 53.3) | 52.2 (49.9 to 54.4) | 51.7 (49.6 to 53.8) | 50.8 (48.4 to 53.2) | 52 (49.9 to 54.1) | 53.9 (51.3 to 56.4) | 55.9 (53.9 to 57.9) | 56.1 (54 to 58.1) | 55 (53.1 to 56.8) | 58.5 (56.6 to 60.4) | 1.01 (1.01 to 1.01) |

**Supplementary Table 7**

**Sensitivity analysis**

**Extremely preterm (EPT) admissions excluding births <24w gestation, care processes by maternal ethnicity and year**

Adjusted risk ratios and 95% confidence intervals (aRR; 95% CI) estimated by modified Poisson regression; adjustment variables are sex, gestational age (weeks), birth weight z-score, and multiplicity; a missing confidence interval indicates that only one individual with that outcome was recorded; this analysis includes 19383 EPT admissions; 3706 were excluded because of missing ethnicity data

|  | **Maternal Ethnicity** | **2013**  **% (95% CI)** | **2014**  **% (95% CI)** | **2015**  **% (95% CI)** | **2016**  **% (95% CI)** | **2017**  **% (95% CI)** | **2018**  **% (95% CI)** | **2019**  **% (95% CI)** | **2020**  **% (95% CI)** | **2021**  **% (95% CI)** | **2022**  **% (95% CI)** | **2023**  **% (95% CI)** | **Overall trend, aRR (95% CI)** |
| --- | --- | --- | --- | --- | --- | --- | --- | --- | --- | --- | --- | --- | --- |
| **Antenatal steroids** | **Asian** | 87 (83.5 to 90.5) | 90.7 (85.8 to 95.5) | 92.2 (88.8 to 95.6) | 92.9 (89.2 to 96.7) | 90.7 (85.8 to 95.6) | 91.0 (88.1 to 94.0) | 92.2 (87.4 to 97.0) | 94.6 (90.2 to 99.0) | 98.4 (96.5 to 100.0) | 89.6 (86.8 to 92.3) | 95.0 (91.8 to 97.2) | 1.0 (1.0 to 1.01) |
|  | **Black** | 91.6 (87.6 to 95.5) | 92.5 (89.2 to 95.8) | 92.8 (88.4 to 97.2) | 90.9 (86.5 to 95.3) | 91.2 (87.2 to 95.1) | 89.6 (85.1 to 94.0) | 94.9 (91.2 to 98.6) | 94.2 (90.5 to 98.0) | 95.5 (92.2 to 98.8) | 94 (90.3 to 97.7) | 91.7 (87.0 to 95.2) | 1.0 (1.0 to 1.0) |
|  | **Mixed** | 84.8 (65.9 to 100.0) | 97.5 (92.6 to 100.0) | 75.1 (58.7 to 91.5) | 89.8 (77.5 to 100.0) | 87.9 (76.2 to 99.7) | 87.9 (75.4 to 100.0) | 96.4 (91.6 to 100.0) | 97.5 (92.6 to 100.0) | 89.7 (78.9 to 100.0) | 85.7 (72.2 to 99.1) | 97.5 (86.8 to 99.9) | 1.0 (0.99 to 1.01) |
|  | **Other** | 90.6 (80.3 to 100.0) | 90.6 (80.3 to 100.0) | 96.2 (91.3 to 100.0) | 76.4 (56.5 to 96.3) | 78.9 (62.0 to 95.7) | 93 (86.6 to 99.4) | 82.1 (60.3 to 100.0) | 94.4 (87.1 to 100.0) | 89.6 (83.0 to 96.3) | 82.3 (73.0 to 91.5) | 87.9 (76.7 to 95.0) | 1.0 (0.99 to 1.0) |
|  | **White** | 89.4 (87.3 to 91.6) | 90.5 (89.0 to 92.0) | 90.1 (88.0 to 92.2) | 89.9 (88.5 to 91.3) | 91.3 (90.1 to 92.5) | 91.0 (89.5 to 92.5) | 91.9 (90.2 to 93.7) | 92.4 (90.7 to 94.0) | 93.5 (92.3 to 94.7) | 90.6 (88.6 to 92.6) | 91.1 (89.2 to 92.7) | 1.0 (1.0 to 1.0) |
| **Birth in a hospital with neonatal intensive care unit** | **Asian** | 71.4 (64.6 to 78.2) | 75.0 (70.0 to 80.0) | 70.2 (63.0 to 77.5) | 76.9 (71.1 to 82.8) | 75.8 (70.9 to 80.8) | 73.8 (66.8 to 80.9) | 76.2 (71.2 to 81.1) | 80.3 (74.7 to 85.9) | 82.1 (78.0 to 86.3) | 78.6 (73.5 to 83.8) | 79.0 (73.8 to 83.6) | 1.01 (1.0 to 1.02) |
|  | **Black** | 70.3 (63.3 to 77.2) | 72.4 (67.8 to 77.1) | 72.7 (65.7 to 79.8) | 72.6 (65.6 to 79.6) | 70.2 (62.9 to 77.5) | 71.8 (65.7 to 78.0) | 78.9 (73.4 to 84.4) | 76.3 (70.8 to 81.9) | 77.0 (69.0 to 85.1) | 79.3 (72.3 to 86.3) | 84.0 (78.1 to 88.9) | 1.01 (1.0 to 1.02) |
|  | **Mixed** | 68.3 (50.9 to 85.8) | 75.8 (55.7 to 96.0) | 59.4 (44.1 to 74.7) | 82.4 (69.7 to 95.1) | 78.5 (65.5 to 91.6) | 83.0 (70.6 to 95.5) | 68.8 (53.1 to 84.5) | 87.3 (70.7 to 100.0) | 84.6 (66.8 to 100.0) | 71.4 (57.7 to 85.1) | 75.0 (58.8 to 87.3) | 1.01 (0.99 to 1.02) |
|  | **Other** | 79.2 (63.2 to 95.3) | 79.6 (64.4 to 94.7) | 70.0 (52.7 to 87.3) | 65.3 (44.7 to 85.9) | 63.9 (42.9 to 84.9) | 82.5 (75.0 to 89.9) | 84.0 (70.7 to 97.2) | 75.0 (55.5 to 94.5) | 76.1 (66.7 to 85.5) | 67.0 (50.5 to 83.5) | 72.4 (59.1 to 83.3) | 0.99 (0.98 to 1.01) |
|  | **White** | 66.6 (64.2 to 69.0) | 66.1 (63.8 to 68.4) | 67.5 (65.0 to 70.0) | 67.2 (65.2 to 69.1) | 68.3 (64.3 to 72.3) | 71.6 (68.6 to 74.6) | 71.0 (67.6 to 74.3) | 73.5 (70.3 to 76.6) | 72.5 (69.3 to 75.7) | 72 (69.5 to 74.5) | 71.8 (69.1 to 74.5) | 1.01 (1.01 to 1.01) |
| **Emergency C-Section** | **Asian** | 41.9 (36.9 to 46.9) | 36.9 (32.1 to 41.7) | 34.9 (26.4 to 43.5) | 37.2 (30.3 to 44.1) | 34.4 (28.6 to 40.1) | 34.6 (26.2 to 42.9) | 37.9 (30.7 to 45.1) | 48.7 (39.9 to 57.5) | 55.5 (48.4 to 62.7) | 48.8 (41.3 to 56.3) | 56.6 (50.5 to 62.5) | 1.04 (1.03 to 1.05) |
|  | **Black** | 35.2 (28.5 to 42.0) | 36.7 (30.5 to 42.9) | 36.9 (30.7 to 43.0) | 36.1 (29.4 to 42.7) | 43.6 (37.7 to 49.5) | 38.2 (30.8 to 45.7) | 49.4 (40.4 to 58.4) | 45.1 (36.0 to 54.3) | 49.5 (41.4 to 57.6) | 52.4 (47.7 to 57.1) | 61.7 (54.3 to 68.7) | 1.05 (1.04 to 1.06) |
|  | **Mixed** | 51.8 (34.2 to 69.5) | 37.5 (19.7 to 55.3) | 44.3 (25.4 to 63.2) | 51.8 (28.8 to 74.8) | 40.1 (21.2 to 58.9) | 26.2 (6.7 to 45.7) | 53.8 (36.1 to 71.5) | 56.0 (38.2 to 73.8) | 55.0 (29.8 to 80.2) | 49.2 (33.5 to 65.0) | 68.4 (51.3 to 82.5) | 1.03 (1.01 to 1.06) |
|  | **Other** | 27.6 (10.6 to 44.6) | 43.9 (22.6 to 65.3) | 34.4 (16.6 to 52.1) | 46.2 (26.5 to 65.9) | 45.8 (22.9 to 68.8) | 44.4 (25.5 to 63.2) | 38.9 (17.2 to 60.5) | 47.5 (27.0 to 68.0) | 49.5 (34.8 to 64.3) | 52.5 (36.9 to 68.1) | 62.5 (48.5 to 75.1) | 1.05 (1.03 to 1.08) |
|  | **White** | 39.6 (36.6 to 42.5) | 38.5 (35.2 to 41.8) | 42.0 (39.6 to 44.3) | 42.7 (40.0 to 45.5) | 41.3 (38.0 to 44.5) | 43.3 (40.3 to 46.3) | 45.5 (41.9 to 49.1) | 47.4 (43.9 to 50.9) | 54.2 (50.1 to 58.3) | 52.0 (50.2 to 53.8) | 55.7 (52.6 to 58.7) | 1.03 (1.03 to 1.04) |
| **Intubation in delivery room** | **Asian** | 90.6 (87.2 to 94.0) | 89.6 (85.5 to 93.8) | 88.9 (84.4 to 93.4) | 84.6 (78.9 to 90.4) | 84.4 (79.5 to 89.2) | 84.3 (81.4 to 87.2) | 75 (67.0 to 83.1) | 67.1 (59.9 to 74.4) | 65.9 (60.4 to 71.4) | 65.7 (62.4 to 69.1) | 60.8 (54.9 to 66.6) | 0.96 (0.95 to 0.96) |
|  | **Black** | 90.2 (86.6 to 93.7) | 88.9 (84.4 to 93.3) | 92.6 (88.5 to 96.8) | 89.9 (86.0 to 93.8) | 86.9 (82.0 to 91.8) | 85.3 (79.3 to 91.3) | 75.7 (68.9 to 82.5) | 80.5 (75.9 to 85.1) | 72.5 (63.6 to 81.3) | 71.1 (62.3 to 79.9) | 64.4 (57.2 to 71.1) | 0.97 (0.96 to 0.97) |
|  | **Mixed** | 85.8 (73.8 to 97.7) | 94.2 (86.4 to 100.0) | 77.7 (61.5 to 93.9) | 88.6 (75.7 to 100.0) | 84.3 (72.2 to 96.5) | 93.2 (83.6 to 100.0) | 74.1 (56.8 to 91.4) | 84.8 (74.4 to 95.3) | 50.5 (32.2 to 68.7) | 55.9 (41.6 to 70.1) | 60.0 (43.3 to 75.1) | 0.96 (0.95 to 0.97) |
|  | **Other** | 89.4 (79.3 to 99.5) | 94.7 (87.6 to 101.8) | 89 (78 to 99.9) | 87.5 (77.8 to 97.2) | 61.4 (41.8 to 80.9) | 71.4 (59.3 to 83.5) | 70.3 (51.1 to 89.6) | 80.2 (66.1 to 94.3) | 65.1 (57.4 to 72.7) | 58.5 (38.5 to 78.5) | 58.6 (44.9 to 71.4) | 0.96 (0.95 to 0.97) |
|  | **White** | 87.3 (85.3 to 89.4) | 86.3 (84.8 to 87.8) | 85.6 (83.7 to 87.4) | 81.8 (79.2 to 84.3) | 80.9 (78.9 to 82.8) | 75.9 (73.7 to 78.1) | 73.5 (69.6 to 77.4) | 70.1 (67.2 to 73.0) | 66.4 (63.3 to 69.6) | 63.8 (60.8 to 66.8) | 60.1 (57.1 to 63.0) | 0.96 (0.96 to 0.97) |
| **Transfer within first 48 hours (any direction)** | **Asian** | 20.9 (15.4 to 26.4) | 16.8 (13.1 to 20.5) | 20.3 (13.3 to 27.3) | 15.3 (9.3 to 21.3) | 13.5 (9.1 to 17.9) | 20.4 (15.8 to 25) | 18.6 (14.1 to 23.2) | 10.6 (5.2 to 16.0) | 11.3 (7.9 to 14.6) | 13.6 (9.2 to 18.0) | 13.2 (9.4 to 17.7) | 0.96 (0.94 to 0.98) |
|  | **Black** | 19.5 (14.6 to 24.5) | 17.2 (11.2 to 23.3) | 16.5 (11.4 to 21.6) | 17.6 (11.8 to 23.3) | 21.8 (15.5 to 28.0) | 19.2 (14.6 to 23.9) | 14.6 (9.4 to 19.9) | 19.8 (14.3 to 25.3) | 15.3 (8.5 to 22.2) | 15.6 (8.4 to 22.7) | 12.4 (8.1 to 17.8) | 0.98 (0.96 to 1.01) |
|  | **Mixed** | 15.4 (0.0 to 34.4) | 20.8 (0.3 to 41.4) | 20.7 (6.8 to 34.6) | 12.4 (0.0 to 24.8) | 14 (1.4 to 26.5) | 10.9 (0.7 to 21.1) | 26.4 (13.4 to 39.3) | 9.3 (0.0 to 22.1) | 4.6 (0.0 to 10.5) | 17.1 (4.6 to 29.6) | 20.0 (9.0 to 35.6) | 1.01 (0.95 to 1.08) |
|  | **Other** | 14.7 (3.0 to 26.4) | 7.6 (0.0 to 15.4) | 9.4 (2.4 to 16.4) | 28.5 (8.7 to 48.2) | 23.3 (5.1 to 41.5) | 20.4 (4.2 to 36.5) | 13.2 (0.4 to 26.0) | 20.1 (0.9 to 39.4) | 17.5 (8.4 to 26.6) | 29.8 (18.1 to 41.5) | 13.8 (6.1 to 25.4) | 1.03 (0.97 to 1.08) |
|  | **White** | 21.3 (19 to 23.6) | 23.2 (20.8 to 25.5) | 22.5 (20.4 to 24.6) | 22.5 (20.6 to 24.5) | 22.0 (18.9 to 25.0) | 21.6 (19.6 to 23.5) | 20.5 (18.1 to 22.9) | 18.0 (15.1 to 20.9) | 20.6 (17.1 to 24.0) | 19.9 (18.3 to 21.4) | 19.6 (17.3 to 22.1) | 0.99 (0.98 to 0.99) |
| **Transfer within first 48 hours (upward)** | **Asian** | 17.9 (12.6 to 23.3) | 16.8 (13.1 to 20.5) | 19.2 (12.6 to 25.9) | 14.2 (8.9 to 19.5) | 12.2 (8.4 to 16.1) | 19.4 (14.5 to 24.3) | 15.2 (10.9 to 19.4) | 13.3 (8.6 to 17.9) | 10.9 (8.2 to 13.6) | 13.7 (10.0 to 17.4) | 11.0 (7.6 to 15.3) | 0.96 (0.93 to 0.98) |
|  | **Black** | 17.2 (12.5 to 21.9) | 17.2 (11.4 to 23.1) | 14.8 (10.1 to 19.6) | 15.6 (10.4 to 20.8) | 21.0 (14.9 to 27.0) | 18.9 (13.9 to 23.9) | 16.4 (12.1 to 20.8) | 19.9 (14.7 to 25.2) | 17.8 (11.2 to 24.4) | 17.9 (12.0 to 23.8) | 11.3 (7.2 to 16.7) | 0.99 (0.96 to 1.02) |
|  | **Mixed** | 56.7 (10.9 to 100.0) | 52.8 (6.3 to 99.3) | 41.4 (27.7 to 55.2) | 25.8 (20.4 to 31.2) | 28.4 (20.1 to 36.7) | 30.0 (15.1 to 44.9) | 41.4 (35.2 to 47.6) | 46.7 (20.5 to 72.8) | 25.0 (25.0 to 25.0) | 31.4 (16.2 to 46.5) | 20.0 (9.0 to 35.6) | 1.02 (0.95 to 1.09) |
|  | **Other** | 32.3 (18.0 to 46.7) | 27.8 (22.3 to 33.2) | 19.8 (15.5 to 24.1) | 50.7 (24.5 to 76.9) | 43.3 (19.0 to 67.7) | 21.7 (18.3 to 25.1) | 41.6 (11.4 to 71.8) | 60.4 (29.6 to 91.3) | 23.6 (13.6 to 33.7) | 35.3 (23.7 to 46.9) | 13.8 (6.1 to 25.4) | 1.03 (0.97 to 1.09) |
|  | **White** | 18.8 (16.8 to 20.7) | 20.5 (18.3 to 22.8) | 19.9 (18.1 to 21.7) | 20.1 (18.3 to 21.9) | 20.1 (17.2 to 23.0) | 19.1 (16.9 to 21.4) | 19.0 (16.4 to 21.6) | 17.4 (14.6 to 20.2) | 19.3 (15.9 to 22.7) | 19.0 (17.2 to 20.8) | 18.5 (16.2 to 20.9) | 0.99 (0.98 to 1.01) |
| **Transfer within first 48 hours (horizontal)** | **Asian** | 5.7  4.5 to 6.9) | 0 | 4.2  (3.2 to 5.3) | 4.3 (3.8 to 4.8) | 7.0 (2.9 to 11.0) | 4.2 | 6.3  (3.3 to 9.2) | 3.9 | 5.6  (4.4 to 6.8) | 4.0  (3 to 4.9) | 1.8 (0.6 to 4.1) | 1.01 (0.91 to 1.12) |
|  | **Black** | 6.9 (5.2 to 8.7) | 9.1 (6.3 to 11.9) | 9.8 (8.4 to 11.2) | 3.7 (2.8 to 4.6) | 4.8  (3.3 to 6.3) | 4.2 | 5.6 | 4.0 | 5.6 | 5.9 | 0.5 (0 to 2.8) | 0.82 (0.70 to 0.96) |
|  | **Mixed** | 0 | 50.0 | 0 | 33.3 | 40.0 | 0 | 0 | 0 | 0 | 0 | 0 | 0.84 (0.68 to 1.04) |
|  | **Other** | 0 | 0 | 33.3 | 25.0 | 20.0 | 100.0 | 33.3 | 0 | 16.7 | 22.5 (17.6 to 27.4) | 0 | 1.02 (0.87 to 1.20) |
|  | **White** | 2.0  (1.3 to 2.7) | 2.5  (1.6 to 3.5) | 2.2  (1.6 to 2.8) | 2.6 (2.2 to 3.0) | 2.2 (  1.4 to 2.9) | 2.3  (1.6 to 3.1) | 1.9  (1.4 to 2.4) | 1.5  (1.1 to 1.9) | 1.8  (1.1 to 2.4) | 1.6  (0.9 to 2.2) | 0.5 (0.2 to 1.2) | 0.89 (0.86 to 0.93) |
| **Transfer within first 48 hours (downward)** | **Asian** | 4.8  (2.8 to 6.7) | 0 | 0 | 9.1 | 0 | 0 | 7.9 (5.5 to 10.3) | 3.9 | 0 | 4.2  (3.5 to 4.9) | 0 | 0.96 (0.77 to 1.18) |
|  | **Black** | 6.8  (5.0 to 8.6) | 10.5 | 0 | 7.9 (0.0 to 17.0) | 0 | 4.0 | 0 | 6.7 | 0 | 4.9  (3.1 to 6.8) | 0 | 0.91 (0.73 to 1.14) |
|  | **Mixed** | 100.0 | 0 | 0 | 10.0 | 0 | 0 | 0 | 0 | 0 | 0 | 0 | 0.65 (0.31 to 1.36) |
|  | **Other** | 16.7 | 0 | 0 | 0 | 0 | 0 | 0 | 0 | 0 | 0 | 0 | Not converged |
|  | **White** | 1.2  (0.8 to 1.5) | 1.4  (0.7 to 2.0) | 0.8  (0.8 to 0.9) | 0.9 (0.9 to 1.0) | 2.0  (2.0 to 2.1) | 1.0  (0.8 to 1.2) | 1.0 | 0 | 0.9 | 1.6 | 0.2 (0 to 0.7) | 0.77 (0.66 to 0.90) |
| **Intubated respiratory support** | **Asian** | 96.8 (95.3 to 98.2) | 96.7 (94.4 to 99.0) | 97.5 (96.0 to 99.0) | 92.2 (88.9 to 95.5) | 92.0 (89.2 to 94.8) | 95.0 (92.6 to 97.5) | 92.3 (88.4 to 96.2) | 86.9 (82.5 to 91.4) | 85.3 (80.2 to 90.4) | 88.1 (83.6 to 92.5) | 84.2 (79.3 to 88.3) | 0.99 (0.98 to 0.99) |
|  | **Black** | 96.0 (93.4 to 98.5) | 96.2 (94.1 to 98.2) | 96.6 (93.8 to 99.3) | 95.7 (93.5 to 98.0) | 97.7 (95.1 to 100.0) | 95.7 (92.3 to 99.1) | 92.3 (89 to 95.7) | 94.2 (91.5 to 97.0) | 88.8 (82.3 to 95.3) | 90.4 (84.1 to 96.8) | 87.0 (81.5 to 91.4) | 0.99 (0.98 to 0.99) |
|  | **Mixed** | 95.2 (88.5 to 100.0) | 96.7 (90.1 to 100.0) | 84.4 (67.6 to 100.0) | 94.7 (87.6 to 100.) | 94.7 (87.6 to 100.0) | 95.4 (86.5 to 100.0) | 95.2 (88.5 to 100.0) | 100 (100 to 100) | 84.7 (72.5 to 96.9) | 87.0 (79.0 to 95.0) | 77.5 (61.5 to 89.2) | 0.99 (0.99 to 0.99) |
|  | **Other** | 93.9 (87.3 to 100.0) | 100 (100 to 100) | 95.8 (87.7 to 100.0) | 97.2 (91.8 to 100.0) | 90.3 (74 to 100.0) | 90.1 (78.0 to 100.0) | 83.5 (66 to 100.0) | 100 (100 to 100) | 92.2 (86.8 to 97.7) | 89.3 (81.6 to 97.0) | 82.8 (70.6 to 91.4) | 0.98 (0.98 to 0.99) |
|  | **White** | 97.6 (97.0 to 98.1) | 96.4 (95.3 to 97.5) | 95.6 (94.4 to 96.7) | 95.2 (94.2 to 96.2) | 95.5 (94.2 to 96.7) | 94.1 (93.0 to 95.1) | 92.2 (91.2 to 93.1) | 91.5 (90.0 to 93.0) | 91.0 (89.4 to 92.5) | 89.0 (86.6 to 91.4) | 88.6 (86.6 to 90.4) | 0.99 (0.99 to 0.99) |
| **PDA closure** | **Asian** | 4.4  (1.9 to 6.9) | 2.3  (0.5 to 4.1) | 3.5  (1.1 to 6.0) | 2.8 (0.5 to 5.2) | 4.7  (2.4 to 7.0) | 2.0 (0.0 to 4.3) | 2.0  (0.0 to 4.1) | 1.2  (0.0 to 2.8) | 1.2  (0.0 to 2.9) | 0.4  (0.0 to 1.1) | 0.4 (0 to 2.0) | 0.84 (0.78 to 0.90) |
|  | **Black** | 6.6  (4.5 to 8.7) | 4.1  (1.4 to 6.8) | 4.4  (1.4 to 7.4) | 3.9 (0.8 to 7.0) | 1.7  (0.2 to 3.2) | 3.4 (1.0 to 5.8) | 0.5  (0.0 to 1.4) | 1.3  (0.0 to 3.0) | 1.0  (0.0 to 2.4) | 1.2  (0.0 to 2.4) | 0.5 (0 to 2.8) | 0.81 (0.74 to 0.88) |
|  | **Mixed** | 6.4 (0.0 to 15.6) | 7.5  (0.0 to 18) | 3.3  (0.0 to 7.8) | 2.3 (0.0 to 6.7) | 4.6 (0.0 to 13.5) | 4.6 (0.0 to 13.5) | 0  (0 to 0) | 0  (0 to 0) | 0  (0 to 0) | 1.5  (0.0 to 4.5) | 0 | 0.79 (0.63 to 0.99) |
|  | **Other** | 8.3 (0.0 to 17.9) | 7.9 (0.0 to 16.1) | 20.7 (0.0 to 42.3) | 6.6 (0.0 to 13.9) | 0 (0 to 0) | 0 (0 to 0) | 2.8 (0.0 to 8.2) | 0 (0 to 0) | 2.1 (0.0 to 6.2) | 0 (0 to 0) | 0 | 0.69 (0.58 to 0.83) |
|  | **White** | 5.4  (3.9 to 6.9) | 3.4  (2.9 to 3.9) | 3.8  (2.7 to 4.8) | 3.3 (2.3 to 4.3) | 2.7  (1.9 to 3.5) | 2.6 (1.8 to 3.5) | 1.9  (1.3 to 2.5) | 1.6  (0.8 to 2.4) | 1.7  (1.0 to 2.5) | 0.5  (0.0 to 1.2) | 0.7 (0.3 to 1.5) | 0.85 (0.82 to 0.88) |
| **Any own mother’s milk at discharge** | **Asian** | 49.5 (42.0 to 57.0) | 46.8 (40.7 to 52.9) | 49.4 (42.0 to 56.9) | 44.3 (37.4 to 51.2) | 48.3 (40.1 to 56.5) | 53.3 (48.1 to 58.5) | 45.7 (37.6 to 53.9) | 60.8 (53.8 to 67.7) | 53.1 (46.0 to 60.1) | 52.9 (47.5 to 58.2) | 59.7 (53.0 to 66.2) | 1.01 (1.0 to 1.02) |
|  | **Black** | 59.5 (55.4 to 63.5) | 59.0 (50.9 to 67.1) | 60.6 (53.0 to 68.2) | 54.2 (48.7 to 59.7) | 49.4 (42.0 to 56.8) | 53.1 (46.6 to 59.7) | 56.8 (44.0 to 69.7) | 55.8 (48.8 to 62.8) | 52.3 (44.1 to 60.6) | 55.5 (47.8 to 63.1) | 74.6 (67.3 to 80.9) | 1.0 (0.99 to 1.01) |
|  | **Mixed** | 42.7 (24.5 to 60.9) | 50.0 (27.6 to 72.4) | 38.6 (21.0 to 56.3) | 37.9 (17.7 to 58.1) | 48.2 (31.6 to 64.8) | 68.3 (47.6 to 89.1) | 30.3 (14.9 to 45.7) | 41.7 (22.6 to 60.8) | 42.7 (25.6 to 59.8) | 53.6 (38.0 to 69.2) | 64.7 (46.5 to 80.2) | 1.04 (1.01 to 1.06) |
|  | **Other** | 50.9 (33.3 to 68.5) | 56.8 (39.7 to 73.9) | 61.8 (42.5 to 81.1) | 70.1 (58.2 to 82.1) | 53.6 (33.4 to 73.9) | 63.3 (45.3 to 81.3) | 51.6 (31.1 to 72.2) | 45.3 (26.9 to 63.7) | 44.7 (27.9 to 61.5) | 50.3 (33.0 to 67.5) | 68.6 (54.1 to 80.9) | 0.99 (0.98 to 1.01) |
|  | **White** | 34.4 (31.7 to 37.1) | 34.7 (31.5 to 37.8) | 32.9 (29.8 to 36.0) | 36.2 (33.2 to 39.3) | 36.5 (33.6 to 39.3) | 38.0 (33.9 to 42.1) | 39.4 (36.4 to 42.4) | 39.8 (36.5 to 43.1) | 39.9 (36.5 to 43.3) | 39.3 (37.3 to 41.2) | 49.1 (45.8 to 52.4) | 1.02 (1.01 to 1.02) |

**Supplementary Table 8A**

**Extremely preterm (EPT) admissions (<28w gestation), clinical outcomes by maternal ethnicity and year**

Adjusted risk ratios and 95% confidence intervals (aRR; 95% CI) estimated by modified Poisson regression; adjustment variables are sex, gestational age (weeks), birth weight z-score, and multiplicity; a missing confidence interval indicates that only one individual with that outcome was recorded; this analysis includes 21907 EPT admissions; 4225 EPT admissions were excluded because of missing ethnicity data

|  | **Maternal Ethnicity** | **2013**  **% (95% CI)** | **2014**  **% (95% CI)** | **2015**  **% (95% CI)** | **2016**  **% (95% CI)** | **2017**  **% (95% CI)** | **2018**  **% (95% CI)** | **2019**  **% (95% CI)** | **2020**  **% (95% CI)** | **2021**  **% (95% CI)** | **2022**  **% (95% CI)** | **2023**  **% (95% CI)** | **Overall trend, aRR (95% CI)** |
| --- | --- | --- | --- | --- | --- | --- | --- | --- | --- | --- | --- | --- | --- |
| **Mortality** | **Asian** | 20.2 (15.3 to 25.2) | 20.5 (14.8 to 26.2) | 21.4 (16.6 to 26.3) | 23.2 (18.1 to 28.3) | 22 (16.7 to 27.3) | 18.2 (13.7 to 22.6) | 19.9 (14.9 to 24.8) | 14.6 (10.5 to 18.7) | 18.9 (13.4 to 24.3) | 21.7 (15 to 28.4) | 25.2 (20.7 to 30.1) | 0.98 (0.97 to 1.01) |
|  | **Black** | 19.8 (14 to 25.5) | 21.1 (18 to 24.3) | 17.5 (13 to 22.1) | 17.6 (13.2 to 22) | 15.7 (11.8 to 19.6) | 19.2 (13.5 to 25) | 18.4 (14.1 to 22.7) | 17.2 (10.1 to 24.4) | 23.4 (17.9 to 29) | 17 (13.4 to 20.7) | 20.2 (15.3 to 25.8) | 0.98 (0.96 to 1.0) |
|  | **Mixed** | 24.9 (8.7 to 41) | 18.9 (7.1 to 30.8) | 13.9 (5 to 22.8) | 19.4 (7.6 to 31.1) | 25.3 (6.7 to 43.9) | 15.8 (0.0 to 32.9) | 15.5 (0.0 to 33.3) | 13.2 (1.2 to 25.2) | 14.2 (3.1 to 25.3) | 21.9 (5.4 to 38.5) | 25.5 (14.3 to 39.6) | 1.01 (0.95 to 1.06) |
|  | **Other** | 26.4 (12.0 to 40.7) | 17.4 (6.1 to 28.7) | 14.4 (6.2 to 22.6) | 7.9 (0.9 to 14.9) | 11.1 (3.7 to 18.5) | 9.5 (3.6 to 15.4) | 16.5 (6.9 to 26.1) | 26 (9.2 to 42.8) | 19.1 (7.9 to 30.4) | 18.8 (5.4 to 32.2) | 19.2 (10.9 to 30.1) | 0.99 (0.95 to 1.04) |
|  | **White** | 21.1 (18.2 to 24.1) | 21.2 (19.8 to 22.6) | 19.9 (17.2 to 22.5) | 19.3 (17.5 to 21.2) | 19.7 (17.4 to 22) | 19.9 (17.5 to 22.3) | 18.3 (15.5 to 21.1) | 20.1 (17.6 to 22.5) | 20.5 (19 to 22) | 23 (20.5 to 25.5) | 21.8 (19.5 to 24.2) | 0.99 (0.98 to 0.99) |
| **Bronchopulmonary dysplasia** | **Asian** | 50.2 (43.9 to 56.6) | 49.4 (43.2 to 55.5) | 49.9 (41.6 to 58.3) | 49.6 (43.2 to 56.1) | 48  (42 to 54) | 52.3 (45.9 to 58.7) | 50 (44.3 to 55.7) | 57.1 (53 to 61.2) | 52 (45.7 to 58.4) | 51.3 (46.1 to 56.6) | 53.2 (47.8 to 58.6) | 1.01 (1.0 to 1.01) |
|  | **Black** | 56.6 (50.1 to 63.1) | 53.6 (47.7 to 59.5) | 53.6 (45.8 to 61.4) | 55.3 (50.4 to 60.1) | 55.7 (50.8 to 60.5) | 58.2 (49.8 to 66.6) | 65 (59.8 to 70.2) | 68.5 (62.7 to 74.2) | 57.3 (49.9 to 64.7) | 60.2 (54.6 to 65.8) | 54.8 (48.3 to 61.3) | 1.01 (1.0 to 1.02) |
|  | **Mixed** | 56 (38.8 to 73.2) | 61.4 (49.4 to 73.3) | 47.7 (30.6 to 64.7) | 42.6 (27.7 to 57.6) | 57.1 (39.9 to 74.2) | 52.2 (26.3 to 78.2) | 55.2 (40.1 to 70.3) | 68.9 (50.6 to 87.2) | 60.6 (42.9 to 78.3) | 47.5 (33.5 to 61.5) | 51.0 (36.6 to 65.2) | 0.99 (0.98 to 1.02) |
|  | **Other** | 63.6 (50.9 to 76.3) | 58.9 (42.1 to 75.8) | 58.1 (44.0 to 72.1) | 59.7 (42.2 to 77.1) | 71.4 (55.9 to 86.9) | 55.9 (39.3 to 72.4) | 51.6 (37.4 to 65.9) | 58.3 (41.0 to 75.6) | 58 (45.6 to 70.5) | 62.5 (50.6 to 74.3) | 68.1 (56.0 to 78.6) | 1.01 (0.99 to 1.03) |
|  | **White** | 56.3 (53.8 to 58.8) | 56.5 (54.5 to 58.5) | 56.4 (53.3 to 59.6) | 55.2 (52.8 to 57.6) | 58.2 (55.1 to 61.4) | 57.3 (54.5 to 60.1) | 59.9 (56.6 to 63.2) | 59.4 (56.3 to 62.4) | 58.3 (55.2 to 61.4) | 55.2 (51.8 to 58.6) | 59.4 (56.6 to 62.1) | 1.01 (1.0 to 1.01) |
| **Severe brain injury** | **Asian** | 23.7 (18.6 to 28.9) | 24.7 (19.3 to 30) | 24.8 (20.5 to 29) | 27.6 (21.9 to 33.3) | 22.5 (18 to 27) | 27.7 (21.3 to 34.1) | 25 (21.7 to 28.3) | 19  (14 to 24) | 24.1 (17.6 to 30.5) | 25.6 (20 to 31.2) | 28.4 (23.7 to 33.5) | 0.99 (0.98 to 1.02) |
|  | **Black** | 21.9 (16.5 to 27.2) | 25.2 (19 to 31.4) | 23.8 (16.8 to 30.9) | 29.5 (21.9 to 37.1) | 28.6 (22.7 to 34.5) | 25.8 (17.1 to 34.5) | 23.6 (18.4 to 28.8) | 23.5 (18.2 to 28.8) | 21.6 (15.2 to 27.9) | 26.2 (20.9 to 31.6) | 19.8 (14.9 to 25.3) | 0.99 (0.97 to 1.01) |
|  | **Mixed** | 24.9 (7.7 to 42) | 24.2 (10.1 to 38.4) | 22 (11.5 to 32.6) | 16.5 (5.3 to 27.7) | 30.1 (10.6 to 49.7) | 16.9 (0.0 to 34.0) | 34.9 (16.6 to 53.1) | 33.9 (12.0 to 55.9) | 31.0 (15.0 to 46.9) | 23.2 (5.6 to 40.8) | 19.2 (9.6 to 32.5) | 1.0 (0.96 to 1.04) |
|  | **Other** | 11.6 (1.7 to 21.4) | 12.6 (3.4 to 21.7) | 33.5 (18.2 to 48.7) | 31.5 (12.7 to 50.3) | 42.8 (24.5 to 61.1) | 15.2 (7.9 to 22.5) | 20.7 (9.2 to 32.2) | 36.8 (17.7 to 55.9) | 18.5 (7.8 to 29.2) | 27.8 (16.9 to 38.7) | 16.4 (8.8 to 27.0) | 0.99 (0.95 to 1.03) |
|  | **White** | 23.6 (20.8 to 26.4) | 23.5 (21.8 to 25.2) | 22.4 (20.2 to 24.6) | 22 (19.6 to 24.3) | 24.9 (22.8 to 26.9) | 23.6 (21.9 to 25.2) | 23.4 (21.2 to 25.5) | 21.5 (18.4 to 24.7) | 24.3 (22 to 26.6) | 24.3 (21 to 27.5) | 24.4 (22.0 to 26.8) | 1.0 (0.99 to 1.01) |
| **Late onset bloodstream infection** | **Asian** | 9.7 (6.4 to 13) | 9.2 (5.9 to 12.6) | 10 (6.4 to 13.5) | 11.6 (7.8 to 15.5) | 10.1 (7.1 to 13.2) | 11.1 (5.9 to 16.4) | 11 (7.5 to 14.6) | 9.8 (6 to 13.6) | 13 (8.4 to 17.5) | 13.5 (9.1 to 17.9) | 13.3 (9.9 to 17.4) | 1.03 (0.99 to 1.06) |
|  | **Black** | 11.3 (7.0 to 15.6) | 10.6 (6.8 to 14.4) | 8.1 (3.5 to 12.8) | 14.5 (8.0 to 21.1) | 14.1 (7.8 to 20.4) | 15.3 (10.6 to 20.1) | 12.8 (7.8 to 17.7) | 18.0 (12.2 to 23.7) | 9.8 (6.1 to 13.6) | 14.5 (10.0 to 18.9) | 17.3 (12.7 to 22.6) | 1.05 (1.02 to 1.08) |
|  | **Mixed** | 5.6 (0.0 to 12.9) | 4.6 (0.0 to 10.5) | 7.1 (0.0 to 14.7) | 3.1 (0.0 to 7.3) | 11.2 (3.6 to 18.9) | 4.4 (0.0 to 10.5) | 23.3 (10.6 to 35.9) | 22.0 (5.0 to 38.9) | 19.0 (1.7 to 36.3) | 9.5 (1.9 to 17.1) | 9.6 (3.2 to 21.0) | 1.07 (0.99 to 1.16) |
|  | **Other** | 17.6 (7.8 to 27.5) | 5.6 (0.0 to 11.4) | 11.8 (0.0 to 28.2) | 20.2 (7.3 to 33.1) | 14.7 (3.8 to 25.7) | 14.2 (5.2 to 23.2) | 5.2 (0.0 to 12) | 24.7 (8.5 to 40.9) | 15.9 (6.7 to 25) | 8.4 (0.3 to 16.5) | 2.7 (0.3 to 9.5) | 0.96 (0.90 to 1.01) |
|  | **White** | 9.2 (7.7 to 10.6) | 8 (6.3 to 9.7) | 9.2 (7.3 to 11.1) | 9.7 (8.8 to 10.7) | 10.1 (8.8 to 11.5) | 10.2 (7.9 to 12.5) | 10.4 (8.1 to 12.6) | 13.7 (11.9 to 15.5) | 11.7 (9.8 to 13.6) | 11.3 (9.5 to 13.1) | 8.8 (7.3 to 10.5) | 1.02 (1.01 to 1.04) |
| **Severe necrotising enterocolitis** | **Asian** | 9.3 (5.8 to 12.7) | 9.3 (5.6 to 13) | 11.2 (7.4 to 15.1) | 7.2  (5.3 to 9) | 10.8 (7 to 14.7) | 9.1 (6.8 to 11.4) | 11.5 (6.8 to 16.3) | 6.4 (3.7 to 9.1) | 9.2 (5.5 to 12.9) | 6.6 (3.8 to 9.4) | 7.5 (5.0 to 10.8) | 0.97 (0.94 to 1.01) |
|  | **Black** | 8.4 (4.6 to 12.1) | 10 (6.5 to 13.5) | 8.3 (5.3 to 11.3) | 9.6 (5.5 to 13.8) | 10.3 (5.4 to 15.2) | 7.9 (4.8 to 11) | 9.5 (6.3 to 12.7) | 10.6 (6.1 to 15.1) | 7.2 (3.3 to 11) | 9.3 (6.4 to 12.2) | 5.8 (3.2 to 9.5) | 0.98 (0.95 to 1.02) |
|  | **Mixed** | 14.4 (0.0 to 30.5) | 0.0 (0.0 to 0.0) | 1.7 (0.0 to 4.9) | 5.6 (0.0 to 13.1) | 3.6 (0.0 to 9.2) | 3.1 (0.0 to 7.1) | 10.6 (0.0 to 21.6) | 23 (0.0 to 46.4) | 17.9 (0.8 to 35.1) | 14.5 (0.0 to 30.6) | 11.5 (4.3 to 23.4) | 1.09 (0.98 to 1.20) |
|  | **Other** | 8 (0.0 to 17.5) | 1.8 (0.0 to 5.4) | 16.8 (0.0 to 34.5) | 6.9 (0.0 to 16.3) | 8.1 (0.6 to 15.5) | 7.4 (0.6 to 14.3) | 11.6 (0.0 to 28.1) | 8.9 (2.0 to 15.9) | 14.4 (0.0 to 30.5) | 14.4 (3.1 to 25.8) | 4.1 (0.9 to 11.5) | 1.0 (0.93 to 1.08) |
|  | **White** | 8.8 (6.9 to 10.7) | 8.1 (6.9 to 9.3) | 8 (6.1 to 10) | 7.5 (6.1 to 8.8) | 9.5 (8 to 10.9) | 8.4 (7.2 to 9.6) | 9.8 (8.3 to 11.2) | 8 (6.7 to 9.3) | 8.4 (6.6 to 10.2) | 7.2 (6.2 to 8.3) | 7.4 (6.0 to 9.0) | 0.99 (0.97 to 1.0) |
| **Treated retinopathy of prematurity** | **Asian** | 17 (12.8 to 21.1) | 16.8 (13.2 to 20.4) | 20.4 (16.2 to 24.6) | 12.8 (8.6 to 17) | 12.3 (8.8 to 15.7) | 16.6 (11.4 to 21.8) | 16.3 (11 to 21.5) | 14.8 (9.9 to 19.8) | 19.8 (15 to 24.6) | 13.6 (10.9 to 16.3) | 15.6 (12.0 to 20.0) | 0.99 (0.96 to 1.01) |
|  | **Black** | 8.9 (5.5 to 12.4) | 8.8 (6 to 11.6) | 7.1 (3.4 to 10.9) | 6.1 (3.4 to 8.8) | 5.6 (4.0 to 7.3) | 5.3 (2.4 to 8.2) | 7.5 (4.7 to 10.4) | 6.9 (4.4 to 9.4) | 6.8 (3.6 to 9.9) | 7.0 (4.2 to 9.9) | 11.9 (8.1 to 16.7) | 1.02 (0.97 to 1.06) |
|  | **Mixed** | 5.8 (0.0 to 14.3) | 11.4 (0.0 to 29.3) | 6.1 (0.0 to 12.5) | 8 (0.0 to 16.8) | 9.2 (0.0 to 20.1) | 2.8 (0.0 to 8.2) | 12.1 (0.0 to 24.6) | 6.4 (0.0 to 13.2) | 24.6 (6.0 to 43.1) | 10.9 (2.4 to 19.4) | 7.7 (2.1 to 18.5) | 1.08 (0.99 to 1.17) |
|  | **Other** | 12.8 (4.7 to 20.9) | 14.4 (0.5 to 28.3) | 18.1 (1.2 to 35.0) | 21.1 (2.7 to 39.5) | 16.4 (5.7 to 27) | 6.5 (-0.3 to 13.4) | 14.0 (4.4 to 23.6) | 22.3 (3.6 to 41.0) | 10.2 (3.4 to 17.1) | 16.1 (4.9 to 27.2) | 16.4 (8.8 to 26.9) | 1.02 (0.96 to 1.08) |
|  | **White** | 11.8 (10.3 to 13.3) | 12.7 (10.7 to 14.7) | 12.9 (10.2 to 15.5) | 9.4 (8.0 to 10.7) | 8.8 (6.8 to 10.9) | 10.0 (7.9 to 12) | 10.2 (8.4 to 12) | 11.7 (9 to 14.4) | 13 (10.5 to 15.4) | 12.8 (10.6 to 15) | 11.3 (9.6 to 13.2) | 0.99 (0.98 to 1.01) |
| **Survival without major morbidity** | **Asian** | 21.7 (17.7 to 25.7) | 23.5 (20.3 to 26.7) | 23.6 (19.3 to 27.8) | 22.1 (17.6 to 26.6) | 25.8 (21.9 to 29.7) | 25.6 (19.1 to 32.0) | 22.4 (18.8 to 25.9) | 24.4 (18.6 to 30.3) | 20.2 (14.5 to 26) | 21.5 (16.3 to 26.7) | 18.4 (14.4 to 22.9) | 0.99 (0.97 to 1.01) |
|  | **Black** | 20.5 (14.1 to 26.9) | 21.7 (15.6 to 27.8) | 23.9 (18.7 to 29.2) | 22.2 (16.0 to 28.5) | 22.7 (18.9 to 26.5) | 18.1 (11.6 to 24.5) | 15.1 (10.8 to 19.4) | 14 (9.6 to 18.3) | 20 (14.8 to 25.2) | 16.9 (11.6 to 22.3) | 23.1 (18.0 to 29.0) | 0.99 (0.97 to 1.01) |
|  | **Mixed** | 17.5 (5.8 to 29.2) | 19.7 (4.1 to 35.3) | 26.8 (9.8 to 43.7) | 33.5 (15.3 to 51.6) | 11.4 (-0.1 to 22.9) | 30.6 (7.2 to 53.9) | 20.2 (6.4 to 34.1) | 15.3 (1.7 to 28.9) | 23.6 (8.3 to 39) | 25.1 (11.7 to 38.5) | 19.6 (9.8 to 33.1) | 1.0 (0.96 to 1.04) |
|  | **Other** | 12.3 (2.3 to 22.4) | 18.0 (6.3 to 29.8) | 19.0 (8.4 to 29.6) | 22.0 (4.8 to 39.3) | 13.1 (1.5 to 24.6) | 33.8 (18.2 to 49.3) | 24.7 (12.7 to 36.6) | 10.0 (0.7 to 19.4) | 21.5 (8.2 to 34.8) | 14.5 (5.6 to 23.5) | 11.0 (4.8 to 20.5) | 0.99 (0.95 to 1.03) |
|  | **White** | 17.0 (15.3 to 18.7) | 18.5 (16.9 to 20.2) | 18.5 (16.2 to 20.8) | 19.8 (17 to 22.5) | 16.6 (14.8 to 18.3) | 18.6 (16.7 to 20.6) | 18 (15.3 to 20.6) | 17.6 (15.1 to 20.0) | 17.0 (13.8 to 20.1) | 17.2 (14.7 to 19.7) | 16.0 (14.0 to 18.2) | 0.99 (0.98 to 1.01) |

**Supplementary Table 8B**

**Very preterm (VPT) admissions (28 - <32w gestation), clinical outcomes by maternal ethnicity and year**

Adjusted risk ratio and 95% confidence interval (aRR; 95% CI) estimated by modified Poisson regression; adjustment variables are sex, gestational age (weeks), birth weight z-score, and multiplicity; a missing confidence interval indicates that only one individual with that outcome was recorded; this analysis includes 46325 VPT admissions; 9464 VPT admissions were excluded because of missing ethnicity data

|  | **Maternal Ethnicity** | **2013**  **%**  **(95% CI)** | **2014**  **%**  **(95% CI)** | **2015**  **%**  **(95% CI)** | **2016**  **%**  **(95% CI)** | **2017**  **%**  **(95% CI)** | **2018**  **%**  **(95% CI)** | **2019**  **%**  **(95% CI)** | **2020**  **%**  **(95% CI)** | **2021**  **%**  **(95% CI)** | **2022**  **%**  **(95% CI)** | **2023**  **% (95% CI)** | **Overall trend, aRR (95% CI)** |
| --- | --- | --- | --- | --- | --- | --- | --- | --- | --- | --- | --- | --- | --- |
| **Mortality** | **Asian** | 3  (1.6 to 4.3) | 3.4  (2 to 4.9) | 1.9  (0.9 to 3) | 3.2 (1.9 to 4.6) | 4.3 (2.8 to 5.7) | 5.2  (2.3 to 8) | 3.6 (1.6 to 5.6) | 3.2  (2.5 to 4) | 3.7 (1.6 to 5.8) | 2.5  (1 to 4.1) | 2.8 (16.6 to 4.4) | 1.0 (0.96 to 1.04) |
|  | **Black** | 3.3 (1.9 to 4.7) | 3  (1.3 to 4.7) | 4.2 (2.1 to 6.3) | 2.7 (1.7 to 3.6) | 2.9  (0.8 to 5) | 2.5  (0.6 to 4.5) | 1.9 (0.1 to 3.7) | 2.1 (0.5 to 3.7) | 4  (2.2 to 5.7) | 3.6 (1.5 to 5.7) | 1.9 (0.8 to 3.8) | 0.97 (0.91 to 1.02) |
|  | **Mixed** | 2.9  (-1.1 to 6.9) | 1.7  (-1.6 to 4.9) | 0.6  (-0.6 to 1.9) | 0.9  (-0.9 to 2.8) | 1.4  (-1.3 to 4.1) | 3.1  (-0.1 to 6.2) | 1.2  (-1.1 to 3.5) | 1.7  (-0.6 to 4.1) | 1.2  (-1.1 to 3.5) | 6.8  (-0.1 to 13.6) | 0 | 1.02 (0.88 to 1.18) |
|  | **Other** | 11.5 (3.2 to 19.8) | 2  (-0.6 to 4.6) | 0  (0 to 0) | 3.3 (0.4 to 6.3) | 2.  4 (-1 to 5.9) | 1.4  (-1.3 to 4.1) | 4.1  (-0.6 to 8.7) | 0  (0 to 0) | 2.6 (-0.1 to 5.4) | 2  (-0.7 to 4.7) | 8.3 (4.0 to 14.7) | 1.01 (0.90 to 1.15) |
|  | **White** | 3.1 (2.5 to 3.8) | 2.8 (2.3 to 3.2) | 3.2 (2.8 to 3.6) | 2.9 (2.4 to 3.4) | 3  (2.3 to 3.7) | 2.3  (1.7 to 2.9) | 2.5  (2 to 3) | 2.7 (2.2 to 3.3) | 2.6  (2 to 3.1) | 2.9 (2.1 to 3.6) | 2.5 (1.9 to 3.1) | 0.98 (0.96 to 1.0) |
| **Bronchopulmonary dysplasia** | **Asian** | 10.4 (7.8 to 13) | 11.2 (8.9 to 13.4) | 12.3 (9.6 to 15) | 13.5 (10.5 to 16.5) | 11.3 (8.3 to 14.3) | 13.1 (9.6 to 16.6) | 14.4 (11 to 17.8) | 14.5 (11.7 to 17.4) | 13.2  (9.1 to 17.2) | 14.7 (11.9 to 17.5) | 13.7 (11.1 to 16.6) | 1.02 (1.01 to 1.04) |
|  | **Black** | 13.3 (10.1 to 16.5) | 13.4 (9.4 to 17.4) | 11.1 (7.6 to 14.6) | 15.3 (10 to 20.5) | 15.1 (11.3 to 18.9) | 16.7 (11.9 to 21.5) | 16.4 (12.4 to 20.4) | 10.8 (7.9 to 13.6) | 14.7 (9.1 to 20.4) | 11 (6 to 16) | 14.8 (11.4 to 18.9) | 1.01 (0.99 to 1.03) |
|  | **Mixed** | 11.8 (5.6 to 18) | 11.9 (4.2 to 19.6) | 20.8 (10.3 to 31.3) | 14.3 (8 to 20.5) | 9.3 (4.7 to 13.9) | 11.5 (6 to 17) | 16.9 (7.3 to 26.5) | 22.1 (10.3 to 33.8) | 17.1 (4.9 to 29.4) | 16.1 (6.9 to 25.3) | 14.0 (7.7 to 22.7) | 1.0 (0.96 to 1.05) |
|  | **Other** | 9.1 (3.9 to 14.3) | 9.9 (1.3 to 18.6) | 15.4 (6.8 to 24) | 19 (12.2 to 25.9) | 30.9 (12.8 to 49) | 23.3 (11.2 to 35.4) | 14.2 (8.8 to 19.6) | 16.3 (8.1 to 24.5) | 14.4 (7.2 to 21.6) | 9.6 (3.1 to 16.2) | 21.8 (14.8 to 30.3) | 1.02 (0.98 to 1.06) |
|  | **White** | 17.4 (16.5 to 18.4) | 18.7 (17.6 to 19.7) | 17.8 (16.2 to 19.4) | 17.1 (15.4 to 18.9) | 18.5 (17.3 to 19.7) | 19 (17.2 to 20.8) | 19.3 (17.8 to 20.9) | 20.2 (17.8 to 22.5) | 19.1 (17.4 to 20.8) | 20.2 (18.8 to 21.6) | 21.0 (19.4 to 22.5) | 1.02 (1.01 to 1.02) |
| **Severe brain injury** | **Asian** | 4.9 (3.5 to 6.3) | 5.4 (4.3 to 6.5) | 4.3 (2.8 to 5.9) | 4.6  (3.2 to 6) | 5.1  (3.1 to 7) | 6.8  (5 to 8.6) | 4.2 (2.3 to 6.1) | 6.2 (4.6 to 7.8) | 6.2 (3.8 to 8.6) | 4.3 (2.8 to 5.8) | 5.3 (3.7 to 7.3) | 1.01 (0.98 to 1.04) |
|  | **Black** | 5.3 (2.8 to 7.8) | 5.6 (2.9 to 8.2) | 6.2 (4.2 to 8.2) | 6.3 (3.8 to 8.8) | 5.4 (2.8 to 8.1) | 4.2 (2.3 to 6.1) | 2.9 (0.7 to 5.1) | 4.4 (2 to 6.8) | 3.8 (1.8 to 5.7) | 4.4 (2.1 to 6.6) | 4.3 (2.5 to 6.8) | 0.96 (0.92 to 1.01) |
|  | **Mixed** | 7.7 (1.2 to 14.2) | 9.3 (0.0 to 20.2) | 8.2 (2.1 to 14.4) | 6  (0.0 to 12) | 8.3 (3.2 to 13.5) | 5.2  (0.4 to 9.9) | 2.9 (0.0 to 6.7) | 10.4 (0.9 to 19.8) | 9.6 (3.7 to 15.5) | 8.3 (1.2 to 15.4) | 5.4 (1.8 to 12.1) | 0.97 (0.90 to 1.05) |
|  | **Other** | 9.5 (2.7 to 16.3) | 7.8 (1.7 to 13.8) | 4.3 (-0.6 to 9.2) | 7.7 (2.2 to 13.2) | 5.2 (1.3 to 9.2) | 4.3  (0.4 to 8.3) | 5.1 (0.1 to 10) | 1.5 (-0.6 to 3.5) | 2.2 (-0.7 to 5.2) | 6.2 (-0.3 to 12.8) | 11.6 (6.5 to 18.7) | 0.98 (0.90 to 1.06) |
|  | **White** | 5.7 (4.7 to 6.8) | 6.2  (5 to 7.4) | 6.1 (5.5 to 6.7) | 6.4  (5.8 to 7) | 5.8 (5.1 to 6.5) | 6  (5.1 to 7) | 6  (4.7 to 7.2) | 5.7 (4.9 to 6.5) | 5.6 (4.6 to 6.6) | 6.8 (5.9 to 7.7) | 6.3 (5.4 to 7.3) | 1.0 (0.99 to 1.01) |
| **Late onset bloodstream infection** | **Asian** | 1.4  (0.8 to 2) | 1.5 (0.7 to 2.3) | 2  (1.3 to 2.6) | 1.8 (0.8 to 2.8) | 1.6 (-0.1 to 3.3) | 1.3  (0.4 to 2.2) | 2.3 (0.7 to 3.9) | 2.3 (0.9 to 3.6) | 3.2 (1.2 to 5.3) | 1.1  (0.2 to 2) | 1.8 (1.0 to 3.2) | 1.03 (0.98 to 1.09) |
|  | **Black** | 1.6 (0.3 to 2.9) | 2.6 (1.3 to 3.9) | 2.7 (0.8 to 4.6) | 1.5 (0.2 to 2.8) | 1.2  (0.3 to 2) | 1.6  (0.2 to 3) | 2.6 (0.7 to 4.5) | 2.8 (1.1 to 4.6) | 2.5 (1.1 to 3.8) | 1.4 (0.2 to 2.6) | 2.1 (0.9 to 4.2) | 0.99 (0.93 to 1.07) |
|  | **Mixed** | 3.  (-1.9 to 9.3) | 1.4  (-1.3 to 4.1) | 2.7  (-0.2 to 5.5) | 2.7  (-0.1 to 5.6) | 1.2  (-1.1 to 3.5) | 1  (-1 to 3.1) | 1  (-1 to 3.1) | 1.2 (-1.1 to 3.5) | 5.5 (-0.7 to 11.7) | 1.7 (-1.6 to 4.9) | 0 | 0.89 (0.78 to 1.03) |
|  | **Other** | 2  (-0.6 to 4.6) | 1.6  (-0.6 to 3.8) | 3  (-0.2 to 6.2) | 0.9  (-0.9 to 2.8) | 3.3  (-0.4 to 6.9) | 0.8  (-0.8 to 2.5) | 1.4  (-1.3 to 4.1) | 1  (-1 to 3.1) | 3.2  (-0.4 to 6.8) | 0  (0 to 0) | 1.6 (0.2 to 5.8) | 0.93 (0.81 to 1.07) |
|  | **White** | 1.9 (1.4 to 2.4) | 2.2 (1.7 to 2.7) | 1.8 (1.3 to 2.2) | 1.9 (1.3 to 2.4) | 2  (1.7 to 2.4) | 2  (1.6 to 2.4) | 1.9 (1.4 to 2.3) | 2  (1.6 to 2.4) | 1.6 (1.1 to 2.1) | 1.8 (1.3 to 2.2) | 1.5 (1.1 to 2.1) | 0.99 (0.96 to 1.01) |
| **Severe necrotising enterocolitis** | **Asian** | 1.3 (0.4 to 2.2) | 1.5 (0.6 to 2.3) | 1.5 (0.5 to 2.5) | 1.6 (0.7 to 2.6) | 1.4 (0.5 to 2.4) | 0.9  (0.1 to 1.8) | 1.4 (0.3 to 2.4) | 1.8 (0.7 to 2.9) | 1.5 (0.2 to 2.9) | 1.2 (0.3 to 2.1) | 1.2 (0.5 to 2.4) | 0.99 (0.93 to 1.05) |
|  | **Black** | 1.4  (0 to 2.7) | 2.1 (0.5 to 3.8) | 3.1 (1.4 to 4.8) | 2.2 (0.6 to 3.7) | 1.1 (0.2 to 2.1) | 0.8  (-0.3 to 1.9) | 1.5 (-0.2 to 3.2) | 1.4 (-0.2 to 3.1) | 2.6 (1.2 to 4.1) | 1.9 (-0.1 to 3.9) | 0.8 (0.2 to 2.3) | 0.98 (0.91 to 1.05) |
|  | **Mixed** | 0  (0 to 0) | 0  (0 to 0) | 0.8  (-0.8 to 2.5) | 1.2  (-1.1 to 3.5) | 3.5  (-0.1 to 7.2) | 0  (0 to 0) | 2.2  (-0.7 to 5.2) | 1.9  (-0.6 to 4.4) | 2.4  (-0.8 to 5.7) | 1.7  (-1.6 to 4.9) | 0 | 1.04 (0.91 to 1.19) |
|  | **Other** | 2.4  (-2.3 to 7) | 0  (0 to 0) | 1.4  (-1.3 to 4.1) | 1.5  (-1.5 to 4.5) | 2.2  (-0.8 to 5.3) | 0  (0 to 0) | 1.6  (-0.5 to 3.7) | 1.2  (-1.1 to 3.5) | 0  (0 to 0) | 2.5  (-0.3 to 5.3) | 0.8 (0 to 4.5) | 0.98 (0.83 to 1.15) |
|  | **White** | 1.4  (1 to 1.7) | 1.6 (0.9 to 2.3) | 1.2 (0.9 to 1.5) | 1.4 (1.1 to 1.6) | 1.4 (0.9 to 1.8) | 1.4  (0.8 to 2) | 1.3 (0.8 to 1.9) | 1.3 (0.8 to 1.8) | 0.9 (0.5 to 1.3) | 1  (0.6 to 1.5) | 0.8 (0.5 to 1.2) | 0.96 (0.93 to 0.98) |
| **Treated retinopathy of prematurity** | **Asian** | 0.6 (0.1 to 1.2) | 1  (0.2 to 1.8) | 1  (0.2 to 1.7) | 0.5  (0 to 1.1) | 0.2  (-0.2 to 0.5) | 0.7  (0 to 1.3) | 0.7  (0 to 1.4) | 0.8  (0 to 1.7) | 0.4  (-0.1 to 1) | 0.5  (0 to 1) | 0.9 (0.3 to 2.0) | 0.99 (0.90 to 1.09) |
|  | **Black** | 0.2  (-0.2 to 0.6) | 0.5  (-0.2 to 1.1) | 0.4  (-0.1 to 1) | 0.5  (-0.2 to 1.1) | 1  (-0.9 to 2.8) | 0  (0 to 0) | 0.6  (-0.2 to 1.3) | 0.7  (-0.3 to 1.7) | 0  (0 to 0) | 0.7  (-0.2 to 1.7) | 1.6 (0.6 to 3.5) | 1.11 (0.96 to 1.27) |
|  | **Mixed** | 0  (0 to 0) | 0  (0 to 0) | 0  (0 to 0) | 3.7  (-1.9 to 9.3) | 0  (0 to 0) | 0  (0 to 0) | 1.7  (-1.6 to 4.9) | 0  (0 to 0) | 0  (0 to 0) | 0  (0 to 0) | 1.1 (0 to 5.8) | 1.01 (0.73 to 1.40) |
|  | **Other** | 1.4  (-1.3 to 4.1) | 0  (0 to 0) | 1.8  (-0.6 to 4.1) | 0  (0 to 0) | 0  (0 to 0) | 2.8  (-2.7 to 8.2) | 0  (0 to 0) | 0  (0 to 0) | 1.9  (-0.6 to 4.4) | 1.2  (-1.1 to 3.5) | 2.5 (0.5 to 7.1) | 1.11 (0.87 to 1.43) |
|  | **White** | 0.9 (0.6 to 1.1) | 0.8  (0.5 to 1) | 0.7  (0.4 to 1) | 0.5 (0.2 to 0.7) | 0.3 (0.1 to 0.5) | 0.4  (0.2 to 0.6) | 0.5 (0.3 to 0.7) | 0.6 (0.3 to 0.9) | 0.7  (0.3 to 1) | 0.6 (0.3 to 0.9) | 0.5 (0.3 to 0.9) | 0.96 (0.91 to 1.0) |
| **Survival without major morbidity** | **Asian** | 82 (80.1 to 84) | 80.9 (78.3 to 83.6) | 79.2 (76.7 to 81.8) | 78.7 (74.8 to 82.6) | 81.2 (77.3 to 85.1) | 77.8 (74.1 to 81.6) | 79.7 (75.5 to 83.9) | 77.4 (73.9 to 80.8) | 78.6 (75 to 82.2) | 79.2 (75.2 to 83.1) | 79.0 (75.6 to 82.1) | 0.99 (0.99 to 0.99) |
|  | **Black** | 79.2 (76.1 to 82.4) | 79.5 (75.4 to 83.7) | 78.9 (74.2 to 83.6) | 78.4 (73.3 to 83.5) | 77.6 (71.9 to 83.2) | 77.3 (72.3 to 82.3) | 80.2 (75.5 to 85) | 81.4 (75.8 to 87) | 76.5 (70.5 to 82.4) | 79.7 (74.2 to 85.2) | 77.6 (73.0 to 81.8) | 0.99 (0.99 to 1.0) |
|  | **Mixed** | 78.7 (68.6 to 88.9) | 79.3 (67.2 to 91.3) | 69.6 (57.3 to 81.9) | 77.3 (66.2 to 88.5) | 81.8 (74.5 to 89.1) | 81.3 (72 to 90.6) | 78.7 (68.6 to 88.9) | 71.7 (60.8 to 82.7) | 73.9 (63.1 to 84.7) | 73.7 (57.6 to 89.9) | 79.6 (69.9 to 87.2) | 1.0 (0.99 to 1.01) |
|  | **Other** | 72.1 (64.2 to 80.1) | 82.3 (73.6 to 91) | 77.3 (66.2 to 88.4) | 72.1 (65.2 to 79) | 61.7 (44.3 to 79) | 72.3 (61.7 to 83) | 78 (71.9 to 84.2) | 80.2 (70.9 to 89.4) | 80.1 (73 to 87.1) | 81.8 (74.3 to 89.2) | 62.2 (52.8 to 70.9) | 0.99 (0.98 to 1.0) |
|  | **White** | 74.2 (72.7 to 75.6) | 73 (71.4 to 74.5) | 74.3 (72.8 to 75.9) | 75.1 (73.6 to 76.6) | 74.5 (73 to 76) | 74.7 (72.7 to 76.8) | 73.9 (71.9 to 75.9) | 72.1 (70 to 74.2) | 73.6 (71.7 to 75.5) | 72.4 (71.1 to 73.7) | 72.8 (71.0 to 74.4) | 0.99 (0.99 to 0.99) |

**Supplementary Table 9**

**Sensitivity analysis**

**Extremely preterm (EPT) admissions, excluding births <24 weeks gestation, clinical outcomes by maternal ethnicity and year**

Adjusted risk ratios and 95% confidence intervals (aRR; 95% CI) estimated by modified Poisson regression; adjustment variables are sex, gestational age (weeks), birth weight z-score, and multiplicity; a missing confidence interval indicates that only one individual with that outcome was recorded; this analysis includes 19383 EPT admissions; 3,706 EPT admissions were excluded because of missing ethnicity data

|  | **Maternal Ethnicity** | **2013**  **% (95% CI)** | **2014**  **% (95% CI)** | **2015**  **% (95% CI)** | **2016**  **% (95% CI)** | **2017**  **% (95% CI)** | **2018**  **% (95% CI)** | **2019**  **% (95% CI)** | **2020**  **% (95% CI)** | **2021**  **%**  **(95% CI)** | **2022**  **%**  **(95% CI)** | **2023**  **% (95% CI)** | **Overall trend, aRR (95% CI)** |
| --- | --- | --- | --- | --- | --- | --- | --- | --- | --- | --- | --- | --- | --- |
| **Bronchopulmonary dysplasia** | **Asian** | 50.9 (44.7 to 57.0) | 49.8 (43.4 to 56.3) | 49.9 (40.8 to 58.9) | 50.7 (43.0 to 58.4) | 48.6 (43.5 to 53.8) | 52.1 (45.8 to 58.4) | 51.0 (45.7 to 56.3) | 56.7 (51.8 to 61.6) | 55.1 (48.5 to 61.8) | 54.4 (50.2 to 58.6) | 55.9 (49.9 to 61.8) | 1.01 (1.0 to 1.02) |
|  | **Black** | 56.9 (49.7 to 64.0) | 55.9 (49.3 to 62.5) | 54.1 (46.8 to 61.4) | 56.8 (51.3 to 62.2) | 52.4 (45.3 to 59.6) | 57.6 (47.6 to 67.5) | 66.7 (60.5 to 73.0) | 70.0 (63.4 to 76.6) | 59.8 (50.7 to 68.8) | 61.8 (52.7 to 70.8) | 58.5 (51.1 to 65.6) | 1.01 (1.0 to 1.02) |
|  | **Mixed** | 59.7 (43.8 to 75.6) | 65.0 (50.2 to 79.8) | 45.2 (27.1 to 63.3) | 40.3 (20.3 to 60.3) | 57.1 (36.9 to 77.3) | 56.7 (30.3 to 83.0) | 60.5 (43.2 to 77.7) | 63.0 (43.9 to 82.1) | 51.4 (31.5 to 71.2) | 55.3 (41.4 to 69.1) | 60.0 (43.3 to 75.1) | 1.0 (0.98 to 1.02) |
|  | **Other** | 67.9 (53.7 to 82.0) | 61.1 (42.1 to 80.0) | 63.4 (50.8 to 76.1) | 59.7 (42.5 to 77.0) | 65.8 (46.4 to 85.2) | 56.8 (40.6 to 73.0) | 40.6 (23.6 to 57.7) | 60.6 (42.5 to 78.8) | 64.7 (50.0 to 79.4) | 71.5 (60.2 to 82.9) | 72.4 (59.1 to 83.3) | 1.01 (0.99 to 1.03) |
|  | **White** | 57.4 (54.6 to 60.1) | 57.8 (55.6 to 59.9) | 57.3 (54.4 to 60.2) | 56.6 (53.8 to 59.4) | 60.1 (56.7 to 63.4) | 59.0 (56.2 to 61.7) | 61.0 (57.5 to 64.5) | 61.1 (58.3 to 64.0) | 60.3 (56.6 to 64.0) | 58.1 (54.7 to 61.5) | 62.2 (59.2 to 65.0) | 1.01 (1.0 to 1.01) |
| **Severe brain Injury** | **Asian** | 23.0 (18.8 to 27.2) | 24.0 (17.5 to 30.5) | 24.2 (20.2 to 28.3) | 25.0 (19.4 to 30.5) | 20.2 (16.5 to 23.9) | 28.1 (19.5 to 36.6) | 22.2 (17.5 to 26.9) | 17.9 (12.9 to 22.9) | 21.2 (13.9 to 28.4) | 23.4 (17.0 to 29.7) | 23.5 (18.7 to 28.9) | 0.99 (0.98 to 1.02) |
|  | **Black** | 19.2 (13.8 to 24.6) | 21.5 (15.0 to 28.0) | 21.7 (15.9 to 27.4) | 27.7 (18.9 to 36.5) | 26.4 (21.4 to 31.3) | 26.0 (15.3 to 36.7) | 23.0 (17.8 to 28.2) | 21.3 (16.7 to 25.9) | 17.9 (10.8 to 25.0) | 21.8 (15.6 to 28.0) | 19.1 (13.8 to 25.3) | 0.99 (0.98 to 1.02) |
|  | **Mixed** | 12.4 (0.0 to 25) | 21.7 (7 to 36.3) | 17.0 (6.0 to 28.0) | 14.6 (2.7 to 26.4) | 34.2 (12.9 to 55.4) | 19.2 (0.8 to 37.7) | 31.4 (12 to 50.7) | 29.2 (9.0 to 49.4) | 13.5 (4.0 to 23.0) | 16.7 (4.4 to 29) | 15.0 (5.7 to 29.8) | 1.01 (0.96 to 1.06) |
|  | **Other** | 10.4 (1.3 to 19.6) | 10.8 (1.5 to 20.0) | 32.6 (15.8 to 49.5) | 31.6 (12.9 to 50.3) | 42.8 (21.2 to 64.4) | 11.9 (4.2 to 19.6) | 22.5 (5.5 to 39.5) | 31.1 (11.3 to 51) | 18.8 (7.2 to 30.5) | 29.6 (13.1 to 46.1) | 12.1 (5.0 to 23.3) | 0.99 (0.94 to 1.03) |
|  | **White** | 23.0 (20.0 to 25.9) | 22.4 (20.6 to 24.1) | 21.6 (19.3 to 23.9) | 21.0 (18.5 to 23.5) | 23.6 (21.5 to 25.7) | 23.2 (21.3 to 25.0) | 21.8 (20.0 to 23.7) | 19.5 (16.5 to 22.4) | 22.8 (21.0 to 24.7) | 20.3 (17.6 to 23.0) | 23.0 (20.5 to 25.6) | 1.0 (0.99 to 1.01) |
| **Late onset bloodstream infection** | **Asian** | 8.9 (5.7 to 12) | 9.9 (5.8 to 14.1) | 9.3 (5.8 to 12.9) | 10.9 (7.6 to 14.3) | 10.2 (7.4 to 13) | 9.6 (5.5 to 13.7) | 10.7 (6.2 to 15.3) | 10 (5.9 to 14.1) | 10.1 (5 to 15.2) | 13 (8.9 to 17.2) | 10.7 (7.3 to 14.9) | 1.03 (0.99 to 1.06) |
|  | **Black** | 11.2 (6.8 to 15.5) | 7.2 (3.4 to 11.0) | 6.7 (3.6 to 9.9) | 12.1 (6.4 to 17.8) | 9.4 (5.8 to 12.9) | 15.7 (10.9 to 20.4) | 12.4 (8.0 to 16.9) | 14.6 (7.5 to 21.7) | 9.3 (6.3 to 12.2) | 13.5 (7.2 to 19.7) | 16.5 (11.6 to 22.5) | 1.06 (1.02 to 1.10) |
|  | **Mixed** | 4.8 (0.0 to 11.5) | 2.5 (0.0 to 7.4) | 3.8 (0.0 to 8.7) | 0  (0 to 0) | 6.7 (0.0 to 13.7) | 5.3 (0.0 to 12.4) | 23.8 (11.2 to 36.4) | 15 (0.0 to 34.6) | 9.1 (0.0 to 19) | 10.7 (0.8 to 20.5) | 10.0 (2.8 to 23.7) | 1.11 (1.01 to 1.23) |
|  | **Other** | 16.2 (7.0 to 25.4) | 5.6 (0.0 to 11.4) | 12.1 (0.0 to 28.5) | 20.5 (7.6 to 33.3) | 9.2 (0.0 to 19.2) | 14.3 (5.4 to 23.3) | 5.2 (0.0 to 12.0) | 22.5 (5.9 to 39.2) | 19.5 (8.2 to 30.8) | 8.8 (0.6 to 17.1) | 1.7 (0 to 9.2) | 0.97 (0.91 to 1.03) |
|  | **White** | 9.0 (7.3 to 10.6) | 7.4 (5.7 to 9.0) | 9.0 (7.1 to 10.8) | 8.8 (7.8 to 9.8) | 9.4 (7.9 to 11.0) | 9.8 (7.6 to 12.1) | 9.7 (7.8 to 11.5) | 12.5 (11.1 to 13.8) | 10.9 (9.0 to 12.8) | 10.5 (8.7 to 12.3) | 8.2 (6.6 to 10.0) | 1.03 (1.01 to 1.04) |
| **Mortality** | **Asian** | 15.9 (10.6 to 21.2) | 16.7 (11.9 to 21.5) | 17.8 (11.8 to 23.9) | 20.4 (16.2 to 24.6) | 17.5 (13.1 to 21.8) | 14.7 (10.0 to 19.4) | 14.7 (11.0 to 18.4) | 12.0 (7.5 to 16.5) | 11.1 (7.2 to 14.9) | 14.3 (8.9 to 19.7) | 17.4 (13.2 to 22.4) | 0.97 (0.95 to 1.0) |
|  | **Black** | 15.5 (10.3 to 20.8) | 13.0 (9.5 to 16.5) | 12.3 (9.0 to 15.7) | 12.5 (7.1 to 17.9) | 13.8 (9.3 to 18.3) | 16.2 (10.8 to 21.6) | 12.7 (9.1 to 16.4) | 12.2 (7.2 to 17.1) | 17.8 (14.3 to 21.3) | 11.2 (8.7 to 13.7) | 11.3 (7.2 to 16.7) | 0.98 (0.95 to 1.01) |
|  | **Mixed** | 15.0 (3.7 to 26.3) | 13.3 (1.9 to 24.8) | 10.6 (1.4 to 19.7) | 20.4 (2.4 to 38.5) | 18.2 (0.0 to 36.6) | 4.6 (0.0 to 13.5) | 9.1 (0.0 to 26.9) | 13.7 (1.9 to 25.4) | 12.1 (1.5 to 22.8) | 8.6 (2.5 to 14.7) | 10.3 (2.9 to 24.2) | 0.99 (0.92 to 1.07) |
|  | **Other** | 22.0 (7.3 to 36.6) | 13.8 (3.0 to 24.6) | 4.9 (0.0 to 10.2) | 7.3 (0.6 to 14.0) | 11.1 (3.7 to 18.5) | 6.2 (1.0 to 11.4) | 11.3 (3.2 to 19.4) | 21.9 (5.5 to 38.2) | 10.3 (1.5 to 19.0) | 9.4 (1.9 to 16.8) | 12.1 (5.0 to 23.3) | 0.98 (0.92 to 1.05) |
|  | **White** | 18.9 (16.0 to 21.8) | 18.2 (16.5 to 19.9) | 17.2 (14.7 to 19.7) | 16.3 (14.3 to 18.3) | 15.9 (13.7 to 18.0) | 15.7 (13.4 to 18.1) | 15.0 (12.8 to 17.2) | 14.9 (12.4 to 17.4) | 15.7 (14.6 to 16.7) | 15.6 (13.0 to 18.2) | 16.1 (14.0 to 18.4) | 0.98 (0.97 to 0.99) |
| **Severe necrotising enterocolitis** | **Asian** | 8.6 (4.4 to 12.9) | 8.3 (4.1 to 12.6) | 11.3 (7.3 to 15.4) | 7.1 (4.8 to 9.3) | 10.6 (6.3 to 14.9) | 7.6 (4.7 to 10.5) | 11.9 (6.5 to 17.4) | 6.1 (3.0 to 9.1) | 7.0 (3.4 to 10.5) | 6.0 (2.9 to 9.0) | 6.8 (1 to 10.4) | 0.98 (0.94 to 1.01) |
|  | **Black** | 6.8 (2.7 to 10.8) | 8.8 (5.3 to 12.2) | 6.9 (3.2 to 10.7) | 10.2 (6.3 to 14.2) | 10.0 (4.3 to 15.6) | 7.0 (3.9 to 10.2) | 9.2 (5.9 to 12.5) | 8.8 (4.0 to 13.5) | 6.4 (3.3 to 9.5) | 8.6 (5.3 to 11.9) | 5.2 (2.5 to 9.3) | 0.98 (0.94 to 1.03) |
|  | **Mixed** | 3.8 (0.0 to 8.9) | 0  (0 to 0) | 0  (0 to 0) | 0  (0 to 0) | 3.6 (0.0 to 10.8) | 4.1 (0.0 to 9.5) | 10.6 (0.0 to 21.6) | 12.0 (0.0 to 31.6) | 6.4 (0.0 to 12.8) | 5.8 (0.0 to 11.8) | 10.0 (2.8 to 23.7) | 1.20 (1.05 to 1.37) |
|  | **Other** | 8.3 (0.0 to 17.9) | 1.8 (0.0 to 5.4) | 16.4 (0.0 to 33.5) | 6.9 (0.0 to 16.3) | 8.1 (0.6 to 15.5) | 6.5 (0.0 to 13.4) | 10.7 (0.0 to 27.3) | 14.0 (0.0 to 30.0) | 16.0 (0.0 to 33.3) | 19.0 (1.7 to 36.3) | 3.4 (0.4 to 11.9) | 0.99 (0.91 to 1.07) |
|  | **White** | 8.9 (7.1 to 10.8) | 7.6 (6.3 to 8.9) | 7.8 (6.0 to 9.6) | 7.1 (5.8 to 8.4) | 9.0 (7.3 to 10.6) | 8.2 (6.7 to 9.8) | 8.9 (7.6 to 10.3) | 7.1 (5.9 to 8.3) | 7.7 (6.3 to 9.0) | 6.1 (5.4 to 6.9) | 6.9 (5.5 to 8.6) | 0.98 (0.97 to 1.0) |
| **Treated retinopathy of prematurity** | **Asian** | 14.8 (11.7 to 17.9) | 15.6 (11.8 to 19.3) | 18.8 (14.9 to 22.7) | 11.5 (7.4 to 15.5) | 11.2 (7.7 to 14.7) | 14.8 (9.3 to 20.3) | 14.7 (9.6 to 19.8) | 12.7 (7.2 to 18.1) | 17.9 (13.1 to 22.7) | 12.7 (9.2 to 16.2) | 13.2 (9.4 to 17.7) | 0.99 (0.96 to 1.02) |
|  | **Black** | 7.4 (4.4 to 10.4) | 5.2 (2.8 to 7.7) | 5.1 (2.7 to 7.5) | 5.3 (2.0 to 8.6) | 3.8 (1.6 to 6.0) | 3.2 (0.3 to 6.1) | 3.9 (0.2 to 7.6) | 5.3 (2.9 to 7.6) | 4.4 (0.9 to 7.8) | 6.0 (2.3 to 9.6) | 9.3 (5.6 to 14.3) | 1.01 (0.95 to 1.07) |
|  | **Mixed** | 7.6 (0.0 to 17.8) | 12.5 (0.0 to 32.2) | 6.1 (0.0 to 12.5) | 4.6 (0.0 to 13.5) | 9.1 (0.0 to 21.0) | 3.6 (0.0 to 10.8) | 12.1 (0.0 to 24.6) | 2.0 (0.0 to 5.9) | 6.8 (0.0 to 16.4) | 10.6 (3.7 to 17.6) | 7.5 (1.6 to 20.4) | 1.05 (0.95 to 1.17) |
|  | **Other** | 8.6 (1.2 to 16.1) | 9.1 (0.0 to 19.3) | 17.0 (0.0 to 34.1) | 20.1 (1.8 to 38.5) | 19.2 (1.7 to 36.7) | 6.9 (0.0 to 14.1) | 13.0 (2.9 to 23.1) | 22.5 (3.6 to 41.4) | 10.4 (2.5 to 18.4) | 22.1 (4.1 to 40.1) | 15.5 (7.3 to 27.4) | 1.05 (0.99 to 1.12) |
|  | **White** | 11.4 (9.8 to 13.1) | 11.6 (9.7 to 13.6) | 12.3 (9.8 to 14.7) | 8.8 (7.5 to 10.1) | 8.1 (5.8 to 10.3) | 8.8 (7.4 to 10.3) | 9.2 (7.4 to 11.0) | 9.6 (7.2 to 12.1) | 11.8 (9.1 to 14.6) | 11.5 (9.8 to 13.2) | 10.4 (8.6 to 12.3) | 0.99 (0.98 to 1.01) |
| **Survival without major morbidity** | **Asian** | 24.6 (20.1 to 29.2) | 25.8 (22.7 to 28.8) | 26.1 (21.3 to 31.0) | 24.2 (18.6 to 29.8) | 28.6 (24.3 to 32.9) | 27.8 (21.2 to 34.5) | 26.1 (21.3 to 31.0) | 26.6 (20.2 to 32.9) | 23.9 (17.7 to 30.1) | 24.6 (19.1 to 30.2) | 22.6 (17.8 to 27.9) | 0.99 (0.98 to 1.01) |
|  | **Black** | 23.5 (16.2 to 30.8) | 27.0 (19.5 to 34.5) | 27.0 (21.2 to 32.8) | 24.4 (18.2 to 30.7) | 26.7 (22.3 to 31.1) | 20.8 (12.9 to 28.6) | 18.3 (13.1 to 23.6) | 16.5 (11.4 to 21.7) | 23.0 (17.1 to 28.9) | 20.3 (14.8 to 25.9) | 28.5 (22.2 to 35.4) | 0.99 (0.97 to 1.01) |
|  | **Mixed** | 22.3 (7.6 to 36.9) | 21.7 (5.0 to 38.4) | 30.1 (9.9 to 50.2) | 34.7 (16.7 to 52.7) | 17 (0.0 to 35.1) | 37 (9.9 to 64.1) | 21.4 (7.3 to 35.4) | 18.5 (2.8 to 34.2) | 34.2 (11.1 to 57.4) | 29.4 (15.7 to 43.2) | 25.0 (12.7 to 41.2) | 1.0 (0.96 to 1.04) |
|  | **Other** | 13.2 (2.9 to 23.4) | 18.8 (6.8 to 30.8) | 21.4 (10.0 to 32.8) | 22.6 (5.3 to 39.9) | 18.6 (0.4 to 36.8) | 36.0 (20.1 to 51.8) | 36.0 (15.5 to 56.5) | 11.4 (1.3 to 21.5) | 23.8 (9.8 to 37.8) | 14.7 (4.4 to 24.9) | 13.8 (6.1 to 25.4) | 0.99 (0.94 to 1.03) |
|  | **White** | 17.8 (16.2 to 19.4) | 20.1 (18.1 to 22.0) | 19.9 (17.5 to 22.3) | 21.1 (18.2 to 24.0) | 18.2 (16.3 to 20.0) | 20.7 (18.7 to 22.7) | 19.9 (17.2 to 22.5) | 20.2 (17.5 to 22.9) | 19.3 (16.0 to 22.6) | 20.5 (17.5 to 23.6) | 18.3 (16.0 to 20.7) | 0.99 (0.98 to 1.01) |

**Supplementary Table 10**

**Model-fit statistics for temporal trend models, 2013–2023**

|  | **Model Type** | **Year Specification** | **AIC (Year continuous)** | **AIC2 (Year categorical)** | **Pearson** $\boldsymbol{\chi^{2}}$ **/df** | **Over- or under-dispersion Interpretation** | **Notes** |
| --- | --- | --- | --- | --- | --- | --- | --- |
| **Admissions**  **EPT**  **VPT**  **Pooled** | Poisson + log-offset | Continuous | 9.06  9.88  51.15 | 9.06  9.88  51.14 | 1.92  1.98  43.01 | Mild  Mild  Severe | Population-level, Offset = log (Live Birth) |
| **Mortality**  **EPT**  **VPT** | Modified Poisson | Continuous | 0.95  0.25 | 0.95  0.26 | 0.80  1.0 | Acceptable  Acceptable | Individual-level, Binary outcome |
| **Bronchopulmonary dysplasia**  **EPT**  **VPT** | Modified Poisson | Continuous | 1.77  0.87 | 1.77  0.87 | 0.43  0.84 | Severe  Acceptable | Individual-level, Binary outcome |
| **Severe brain injury**  **EPT**  **VPT** | Modified Poisson | Continuous | 1.12  0.44 | 1.12  0.44 | 0.76  0.94 | Acceptable Acceptable | Individual-level, Binary outcome |
| **Late onset bloodstream infection**  **EPT**  **VPT** | Modified Poisson | Continuous | 0.68  0.18 | 0.68  0.18 | 0.89  0.99 | Acceptable  Acceptable | Individual-level, Binary outcome |
| **Severe necrotising enterocolitis**  **EPT**  **VPT** | Modified Poisson | Continuous | 0.56  0.13 | 0.57  0.13 | 0.91  1.02 | Acceptable  Acceptable | Individual-level, Binary outcome |
| **Treated retinopathy of prematurity**  **EPT**  **VPT** | Modified Poisson | Continuous | 0.67  0.07 | 0.67  0.07 | 0.87  1.09 | Acceptable  Acceptable | Individual-level, Binary outcome |
| **Survival without major morbidity**  **EPT**  **VPT** | Modified Poisson | Continuous | 0.91  1.91 | 0.91  1.91 | 0.76  0.24 | Acceptable  Acceptable | Individual-level, Binary outcome |
| **Any antenatal steroids**  **EPT**  **VPT** | Modified Poisson | Continuous | 2.0  2.0 | 2.0  2.0 | 0.09  0.08 | Severe  Severe | Individual-level, Binary outcome |
| **Birth in hospital with a Neonatal Intensive Care Unit (tertiary neonatal unit)**  **EPT**  **VPT** | Modified Poisson | Continuous | 1.9  1.67 | 1.9  1.67 | 0.28  0.51 | Severe  Mild | Individual-level, Binary outcome |
| **Birth by emergency Caesarean section**  **EPT**  **VPT** | Modified Poisson | Continuous | 1.48  1.80 | 1.48  1.81 | 0.56  0.39 | Mild  Severe | Individual-level, Binary outcome |
| **Intubation in delivery room**  **EPT**  **VPT** | Modified Poisson | Continuous | 1.93  1.18 | 1.93  1.18 | 0.22  0.72 | Severe  Mild | Individual-level, Binary outcome |
| **Transfer within first 48 hours (any direction)**  **EPT**  **VPT** | Modified Poisson | Continuous | 1.02  0.67 | 1.02  0.67 | 0.80  0.90 | Acceptable  Acceptable | Individual-level, Binary outcome |
| **Transfer within first 48 hours (upwards)**  **EPT**  **VPT** | Modified Poisson | Continuous | 0.96  0.51 | 0.96  0.51 | 0.82  0.93 | Acceptable Acceptable | Individual-level, Binary outcome |
| **Transfer within first 48 hours (horizontal)**  **EPT**  **VPT** | Modified Poisson | Continuous | 0.13  0.15 | 0.14  0.15 | 1.0  0.98 | Acceptable  Acceptable | Individual-level, Binary outcome |
| **Transfer within first 48 hours (downwards)**  **EPT**  **VPT** | Modified Poisson | Continuous | 0.04  0.16 | 0.04  0.16 | 1.05  0.98 | Acceptable  Acceptable | Individual-level, Binary outcome |
| **Any intubated respiratory support**  **EPT**  **VPT** | Modified Poisson | Continuous | 2.0  1.60 | 2.0  1.60 | 0.06  0.52 | Severe  Mild | Individual-level, Binary outcome |
| **PDA closure by ligation or device**  **EPT**  **VPT** | Modified Poisson | Continuous | 0.23  0.36 | 0.24  0.37 | 0.96  1.43 | Acceptable  Mild | Individual-level, Binary outcome |
| **Receiving any own mother’s milk at discharge**  **EPT**  **VPT** | Modified Poisson | Continuous | 1.71  1.82 | 1.71  1.82 | 0.49  0.39 | Severe  Severe | Individual-level, Binary outcome |

**Supplementary Table 11**

**Sensitivity analyses using Negative binomial regression**

To assess the robustness of results from the modified Poisson models for individual-level care-process and clinical outcomes, we conducted sensitivity analyses using negative binomial regression. Although the primary outcomes were binary, negative binomial models were fitted as an alternative specification that relaxes the Poisson mean–variance equality and permits extra-Poisson variation. Models included the same covariates as the primary analyses and modelled birth year as a continuous variable to estimate the average annual change. Results from the negative binomial models were compared with those from the modified Poisson regression in terms of direction, magnitude, and statistical inference; findings were highly consistent across modelling approaches.

|  | **Overall trend** | **95% CI** |
| --- | --- | --- |
| **Admissions**  **EPT**  **VPT**  **Pooled** | 1.01  0.99  1.0 | 1.0 to 1.02  0.99 to 1.0  0.99 to 1.01 |
| **Mortality**  **EPT**  **VPT** | 0.98  0.98 | 0.97 to 0.99  0.97 to 1.0 |
| **Bronchopulmonary dysplasia**  **EPT**  **VPT** | 1.01  1.01 | 1.0 to 1.01  1.0 to 1.02 |
| **Severe brain injury**  **EPT**  **VPT** | 1.0  0.99 | 0.99 to 1.01  0.98 to 1.01 |
| **Late onset bloodstream infection**  **EPT**  **VPT** | 1.03  0.99 | 1.02 to 1.04  0.97 to 1.01 |
| **Severe necrotising enterocolitis**  **EPT**  **VPT** | 0.99  0.96 | 0.98 to 1.0  0.94 to 0.98 |
| **Treated retinopathy of prematurity**  **EPT**  **VPT** | 0.99  0.96 | 0.98 to 1.01  0.93 to 1.0 |
| **Survival without major morbidity**  **EPT**  **VPT** | 0.99  0.99 | 0.98 to 1.0  0.99 to 0.99 |
| **Any antenatal steroids**  **EPT**  **VPT** | 1.0  1.0 | 1.0 to 1.01  1.0 to 1.0 |
| **Birth in hospital with a Neonatal Intensive Care Unit (tertiary neonatal unit)**  **EPT**  **VPT** | 1.01  1.01 | 1.0 to 1.01  1.0 to 1.01 |
| **Birth by emergency Caesarean section**  **EPT**  **VPT** | 1.04  1.02 | 1.03 to 1.04  1.01 to 1.02 |
| **Intubation in delivery room**  **EPT**  **VPT** | 0.96  0.93 | 0.96 to 0.97  0.93 to 0.93 |
| **Transfer within first 48 hours (any direction)**  **EPT**  **VPT** | 0.98  1.0 | 0.97 to 0.99  0.99 to 1.01 |
| **Transfer within first 48 hours (upwards)**  **EPT**  **VPT** | 0.98  1.01 | 0.97 to 0.99  1.0 to 1.02 |
| **Transfer within first 48 hours (horizontal)**  **EPT**  **VPT** | 0.91  0.97 | 0.88 to 0.95  0.95 to 0.99 |
| **Transfer within first 48 hours (downwards)**  **EPT**  **VPT** | 0.81  0.96 | 0.73 to 0.90  0.94 to 0.98 |
| **Any intubated respiratory support**  **EPT**  **VPT** | 0.99  0.97 | 0.98 to 0.99  0.96 to 0.97 |
| **PDA closure by ligation or device**  **EPT**  **VPT** | 0.82  0.86 | 0.80 to 0.84  0.81 to 0.92 |
| **Receiving any own mother’s milk at discharge**  **EPT**  **VPT** | 1.01  1.01 | 1.01 to 1.02  1.0 to 1.01 |

**Supplementary Table 12**

**Baseline characteristics of infants included in complete-case analyses compared with those with missing baseline data**

| **Characteristic** | **Complete cases** | **Incomplete cases** | **Overall** |
| --- | --- | --- | --- |
| **Number of infants, n** | **67637** | **14284** | **81921** |
| **Gestational weeks, Mean (SD)** | **28.4 (2.4)** | **28.3 (2.5)** | **28.4 (2.4)** |
| **Birth weight z-score, Mean (SD)** | **-0.34 (0.94)** | **-0.33 (0.94)** | **-0.34 (0.94)** |
| **Male, n (%)** | **37115 (54.9)** | **7828 (54.8)** | **44943 (54.9)** |
| **Multiplicity, n (%)** | **16995 (25.1)** | **3499 (24.5)** | **20494 (25.0)** |
| **Antenatal steroids, n (%)** | **61838 (91.4)** | **12644 (90.4)** | **74482 (91.2)** |

**Complete cases were defined as infants with non-missing data on maternal ethnicity and baseline covariates included in the regression models (gestational age, birth weight z-score, sex, multiplicity, antenatal steroids). Outcome and care-process variables were not used to define completeness to avoid conditioning on post-baseline information.**

**Supplementary Table 13**

**Care processes by year; extremely preterm (EPT) admissions**

Adjusted risk ratio and 95% confidence interval (aRR; 95% CI) estimated by modified Poisson regression; reference year is 2013; adjustment variables are sex, gestational age (weeks), birth weight z-score, and multiplicity; this analysis includes 26132 EPT admissions

|  | **2013**  **% (95% CI)** | **2014**  **% (95% CI)** | **2015**  **% (95% CI)** | **2016**  **% (95% CI)** | **2017**  **% (95% CI)** | **2018**  **% (95% CI)** | **2019**  **% (95% CI)** | **2020**  **% (95% CI)** | **2021**  **% (95% CI)** | **2022**  **% (95% CI)** | **2023**  **% (95% CI)** | **Overall trend** |
| --- | --- | --- | --- | --- | --- | --- | --- | --- | --- | --- | --- | --- |
| **Any antenatal steroids**  **aRR (95% CI)** | 88.0 (86.76 to 89.33)  **Ref** | 89.56 (88.34 to 90.79)  1.02 (1.0 to 1.03) | 89.12 (87.89 to 90.35)  1.01 (1.0 to 1.02) | 89.19 (87.97 to 90.41)  1.01 (1.0 to1.02) | 90.16 (88.97 to 91.35)  1.02 (1.01 to 1.04) | 90.37 (89.18 to 91.56)  1.03 (1.01 to 1.04) | 91.38 (90.24 to 92.52)  1.04 (1.03 to 1.05) | 92.78 (91.68 to 93.87)  1.06 (1.04 to 1.07) | 92.51 (91.44 to 93.58)  1.06 (1.04 to 1.07) | 89.90 (88.68 to 91.12)  1.03 (1.01 to 1.04) | 90.92 (89.75 to 92.10)  1.04 (1.03 to 1.05) | 90.38 (90.04 to 90.73)  1.0 (1.0 to 1.01) |
| **Birth in hospital with a Neonatal Intensive Care Unit (tertiary neonatal unit)**  **aRR (95% CI** | 67.90 (66.05 to 69.75)  **Ref** | 68.27 (66.41 to 70.13)  1.0 (0.98 to 1.03) | 69.0 (67.18 to 70.82)  1.01 (0.99 to 1.04) | 70.18 (68.38 to 71.98)  1.04 (1.01 to 1.07) | 69.84 (68.0 to 71.67)  1.03 (1.0 to 1.06) | 72.31 (70.51 to 74.11)  1.06 (1.03 to 1.09) | 73.92 (72.14,75.70)  1.08 (1.05 to 1.11) | 76.74 (74.96 to 78.53)  1.12 (1.10 to 1.15) | 75.28 (73.52 to 77.03)  1.11 (1.08 to 1.14) | 74.40 (72.64 to 76.17)  1.09 (1.06 to 1.12) | 75.48 (73.72 to 77.23)  1.10 (1.07 to 1.12) | 72.24 (71.71 to 72.77)  1.01 (1.0 to 1.01) |
| **Birth by emergency Caesarean section**  **aRR (95% CI** | 37.62 (35.66 to 39.58)  **Ref** | 36.80 (34.83 to 38.77)  1.0 (0.94 to 1.06) | 38.74 (36.79 to 40.70)  1.05 (0.99 to 1.11) | 39.39 (37.44 to 41.35)  1.05 (0.99 to 1.11) | 37.84 (35.86 to 39.81)  1.03 (0.97 to 1.09) | 40.13 (38.12 to 42.14)  1.10 (1.04 to 1.16) | 40.68 (38.67 to 42.69)  1.11 (1.05 to 1.17) | 42.80 (40.68 to 44.92)  1.18 (1.12 to 1.25) | 50.11 (48.02 to 52.20)  1.36 (1.29 to 1.43) | 47.63 (45.55 to 49.71)  1.36 (1.29 to 1.43) | 50.40 (48.32 to 52.49)  1.39 (1.32 to 1.47) | 42.34 (41.74 to 42.93)  1.04 (1.03 to 1.04) |
| **Intubation in delivery room**  **aRR (95% CI** | 87.67 (86.37 to 88.97)  **Ref** | 87.29 (85.95 to 88.62)  0.99 (0.98 to 1.0) | 87.20 (85.88 to 88.51)  0.99 (0.98 to 1.0) | 84.37 (82.95 to 85.80)  0.96 (0.95 to 0.98) | 82.84 (81.33 to 84.34)  0.94 (0.92 to 0.95) | 79.52 (77.89 to 81.14)  0.90 (0.88 to 0.91) | 76.64 (74.93 to 78.35)  0.87 (0.85 to 0.88) | 73.78 (71.93 to 75.64)  0.83 (0.81 to 0.84) | 68.48 (66.59 to 70.37)  0.77 (0.76 to 0.79) | 67.78 (65.89 to 69.67)  0.75 (0.74 to 0.77) | 64.12 (62.16 to 66.08)  0.71 (0.70 to 0.73) | 77.08 (76.59 to 77.58)  0.96 (0.96 to 0.97) |
| **Transfer within first 48 hours (any direction)**  **aRR (95% CI** | 21.19  (19.58 to 22.81)  **Ref** | 20.98  (19.35 to 22.61)  0.98 (0.89 to 1.09) | 20.73  (19.14 to 22.33)  0.97 (0.87 to 1.07) | 20.35  (18.77 to 21.93)  0.96 (0.87 to 1.06) | 20.97  (19.34 to 22.59)  0.99 (0.89 to 1.10) | 19.93  (18.32 to 21.54)  0.94 (0.84 to 1.04) | 18.75  (17.17 to 20.34)  0.87 (0.78 to 0.97) | 16.37  (14.80 to 17.93)  0.76 (0.68 to 0.86) | 17.99  (16.43 to 19.55)  0.84 (0.75 to 0.94) | 18.10  (16.54 to 19.66)  0.85 (0.76 to 0.95) | 17.14 (15.60 to 18.67)  0.84 (0.75 to 0.93) | 19.29 (18.83 to 19.76)  0.98 (0.97 to 0.99) |
| **Transfer within first 48 hours (upwards)**  **aRR (95% CI** | 19.04  (17.49 to 20.59)  **Ref** | 18.99  (17.42 to 20.55)  0.99 (0.89 to 1.11) | 18.72  (17.18 to 20.25)  0.97 (0.87 to 1.08) | 18.23  (16.71 to 19.74)  0.96 (0.86 to 1.07) | 18.94  (17.38 to 20.50)  0.99 (0.89 to 1.11) | 18.24  (16.69 to 19.80)  0.95 (0.85 to 1.06) | 17.22  (15.69 to 18.75)  0.88 (0.79 to 0.99) | 15.72  (14.18 to 17.25)  0.81 (0.72 to 0.92) | 16.79  (15.27 to 18.31)  0.86 (0.77 to 0.97) | 17.04  (15.52 to 18.56)  0.87 (0.77 to 0.98) | 15.75 (14.26 to 17.24)  0.85 (0.76 to 0.96) | 17.62 (17.17 to 18.06)  0.98 (0.97 to 0.99) |
| **Transfer within first 48 hours (horizontal)**  **aRR (95% CI** | 1.74  (1.23 to 2.27)  **Ref** | 1.50  (1.01 to 1.98)  0.87 (0.56 to 1.34) | 1.81  (1.28 to 2.33)  1.01 (0.67 to 1.54) | 1.84  (1.31 to 2.37)  1.04 (0.69 to 1.57) | 1.82  (1.29 to 2.35)  1.05 (0.69 to 1.60) | 1.18  (0.74 to 1.61)  0.71 (0.44 to 1.14) | 1.24  (0.78 to 1.68)  0.74 (0.46 to 1.18) | 0.46  (0.18 to 0.75)  0.28 (0.14 to 0.56) | 0.94  (0.55 to 1.33)  0.57 (0.34 to 0.95) | 0.72  (0.38 to 1.06)  0.45 (0.26 to 0.79) | 0.78 (0.42 to 1.14)  0.48 (0.28 to 0.83) | 1.25 (1.12 to 1.38)  0.91 (0.88 to 0.95) |
| **Transfer within first 48 hours (downwards)**  **aRR (95% CI** | 0.89  (0.52 to 1.27)  **Ref** | 0.54  (0.25 to 0.83)  0.60 (0.30 to1.19) | 0.08  (0 to 0.19)  0.09 (0.02 to 0.38) | 0.24  (0.05 to 0.43)  0.26 (0.11 to 0.65) | 0.21  (0.02 to 0.39)  0.23 (0.09 to 0.61) | 0.17  (0 to 0.33)  0.19 (0.06 to 0.55) | 0.17  (0 to 0.34)  0.19 (0.07 to 0.57) | 0.09  (0 to 0.22)  0.10 (0.02 to 0.45) | 0.08  (0 to 0.20)  0.10 (0.02 to 0.41) | 0.21 (  0.02 to 0.40)  0.25 (0.10 to 0.67) | 0.13 (0 to 0.28)  0.15 (0.05 to 0.51) | 0.26 (0.20 to 0.32)  0.81 (0.73 to 0.90) |
| **Any intubated respiratory support**  **aRR (95% CI** | 97.53 (96.92 to 98.15)  **Ref** | 97.03 (96.35 to 97.71)  0.99 (0.99 to 0.99) | 96.35 (95.62 to 97.09)  0.99 (0.98 to 0.99) | 95.46 (94.64 to 96.28)  0.98 (0.97 to 0.98) | 95.73 (94.92 to 96.53)  0.98 (0.97 to 0.98) | 94.66 (93.75 to 95.57)  0.97 (0.96 to 0.97) | 93.74 (92.76 to 94.72)  0.96 (0.95 to 0.96) | 93.43 (92.38 to 94.48)  0.95 (0.94 to 0.96) | 90.96 (89.79 to 92.13)  0.93 (0.92 to 0.94) | 91.11 (89.96 to 92.27)  0.92 (0.92 to 0.93) | 88.65 (87.34 to 89.95)  0.90 (0.89 to 0.91) | 93.70 (93.41 to 93.98)  0.99  (0.99 to 0.99) |
| **PDA closure by ligation or device**  **aRR (95% CI** | 5.66 (4.74 to 6.58)  **Ref** | 4.10 (3.29 to 4.89)  0.72 (0.56 to 0.92) | 4.43 (3.61 to 5.24)  0.77 (0.60 to 0.98) | 3.57 (2.83 to 4.30)  0.63 (0.49 to 0.82) | 2.84 (2.18 to 3.51)  0.50 (0.37 to 0.66) | 2.82 (2.15 to 3.49)  0.47 (0.35 to 0.63) | 2.0 (1.42 to 2.57)  0.34 (0.24 to 0.47) | 1.42 (0.92 to 1.93) 0.24 (0.16 to 0.36) | 1.49 (1.0 to 1.98) 0.26 (0.18 to 0.38) | 0.61 (0.29 to 0.93) 0.11 (0.06 to 0.18) | 0.66 (0.33 to 1.0)  0.10 (0.06 to 0.18) | 2.58 (2.39 to 2.76)  0.82 (0.80 to 0.84) |
| **Receiving any own mother’s milk at discharge**  **aRR (95% CI** | 49.20 (46.94 to 51.46)  **Ref** | 47.89 (45.61 to 50.17)  0.97 (0.92 to 1.03) | 47.68 (45.45 to 49.92)  0.97 (0.92 to 1.02) | 50.28 (48.04 to 52.52)  1.02 (0.97 to 1.08) | 48.62 (46.37 to 50.88)  0.99 (0.94 to 1.04) | 51.43 (49.15 to 53.72)  1.05 (0.99 to 1.10) | 51.46 (49.13 to 53.78)  1.05 (0.99 to 1.10) | 56.44 (54.03 to 58.85)  1.15 (1.10 to 1.21) | 52.11 (49.77 to 54.44)  1.06 (1.01 to 1.12) | 53.26 (50.94 to 55.58)  1.10 (1.05 to 1.16) | 55.13 (52.77 to 57.48)  1.12 (1.06 to 1.18) | 51.37 (50.69 to 52.04)  1.01 (1.01 to 1.02) |

**The “Overall trend” column represents the adjusted relative risk (aRR) for each one-year increase in birth year, derived from modified Poisson regression models adjusted for baseline covariates.**

**Supplementary Table 14**

**Care processes by year; very preterm (VPT) admissions**

Adjusted risk ratio and 95% confidence interval (aRR; 95% CI) estimated by modified Poisson regression; reference year is 2013; adjustment variables are sex, gestational age (weeks), birth weight z-score, and multiplicity; this analysis includes 55789 VPT admissions

|  | **2013**  **% (95% CI)** | **2014**  **% (95% CI)** | **2015**  **% (95% CI)** | **2016**  **% (95% CI)** | **2017**  **% (95% CI)** | **2018**  **% (95% CI)** | **2019**  **% (95% CI)** | **2020**  **% (95% CI)** | **2021**  **% (95% CI)** | **2022**  **% (95% CI)** | **2023**  **% (95% CI)** | Overall trend |
| --- | --- | --- | --- | --- | --- | --- | --- | --- | --- | --- | --- | --- |
| **Any antenatal steroids**  **aRR (95% CI)** | 90.18 (89.38 to 90.97)  **Ref** | 89.90 (89.10 to 90.72)  0.99 (0.98 to 1.0) | 91.02 (90.26 to 91.77)  1.0 (1.0 to 1.01) | 90.71 (89.94 to 91.49)  1.0 (0.99 to1.0) | 91.57 (90.83 to 92.31)  1.01 (1.0 to 1.02) | 92.27 (91.53 to 93.0)  1.02 (1.01 to 1.03) | 92.88 (92.17 to 93.58)  1.03 (1.02 to 1.03) | 93.30 (92.58 to 94.03)  1.03 (1.02 to 1.04) | 92.68 (91.93 to 93.42)  1.03 (1.02 to 1.04) | 92.69 (91.94 to 93.44)  1.03 (1.02 to 1.03) | 91.96 (91.17 to 92.74)  1.02 (1.01 to 1.03) | 91.67 (91.45 to 91.90)  1.0 (1.0 to 1.0) |
| **Birth in hospital with a Neonatal Intensive Care Unit (tertiary level unit)**  **aRR (95% CI)** | 46.03 (44.70 to 47.36)  **Ref** | 47.70 (46.36 to 49.04)  1.03 (99 to 1.07) | 49.01 (47.69 to 50.33)  1.06 (1.03 to 1.10) | 48.60 (47.26 to 49.92)  1.05 (1.02 to 1.09) | 49.70 (48.38 to 51.03)  1.07 (1.04 to 1.11) | 49.76 (48.38 to 51.14)  1.08 (1.04 to 1.12) | 49.90 (48.52 to 51.28)  1.08 (1.04 to 1.12) | 49.77 (48.32 to 51.22)  1.08 (1.04 to 1.11) | 51.20 (49.77 to 52.63)  1.12 (1.08 to 1.15) | 49.17 (47.72 to 50.62)  1.08 (1.04 to 1.12) | 49.42 (47.98 to 50.87)  1.07 (1.04 to 1.11) | 49.13 (48.72 to 49.53)  1.0 (1.0 to 1.01) |
| **Birth by emergency Caesarean section**  **aRR (95% CI)** | 56.43 (55.07 to 57.78)  **Ref** | 58.65 (57.30 to 60.0)  1.03 (1.0 to 1.06) | 57.58 (56.25 to 58.91)  1.02 (0.99 to 1.04) | 56.92 (55.57 to 58.26)  1.01 (0.98 to 1.03) | 59.25 (57.91 to 60.59)  1.04 (1.02 to 1.07) | 60.19 (58.80 to 61.57)  1.06 (1.04 to 1.09) | 59.40 (58.01 to 60.78)  1.04 (1.02 to 1.07) | 62.24 (60.79 to 63.70)  1.10 (1.07 to 1.13) | 64.21 (62.79 to 65.64)  1.14 (1.11 to 1.17) | 67.53 (66.12 to 68.94)  1.20 (1.17 to 1.23) | 67.99 (66.60 to 69.37)  1.20 (1.17 to 1.23) | 61.11 (60.70 to 61.51)  1.02 (1.01 to 1.02) |
| **Intubation in delivery room**  **aRR (95% CI)** | 35.89 (34.62 to 37.17)  **Ref** | 33.35 (31.94 to 34.43)  0.94 (0.90 to 0.98) | 33.18 (31.94 to 34.43)  0.94 (0.90 to 0.98) | 32.63 (31.38 to 33.87)  0.91 (0.87 to 0.95) | 30.11 (28.89 to 31.33)  0.84 (0.80 to 0.88) | 27.76 (26.52 to 28.99)  0.78 (0.74 to 0.82) | 25.45 (24.25 to 26.65)  0.71 (0.68 to 0.75) | 23.77 (22.54 to 25.0)  0.65 (0.62 to 0.69) | 22.40 (21.20 to 23.60)  0.63 (0.59 to 0.67) | 17.93 (16.82 to 19.04)  0.50 (0.47 to 0.54) | 16.99 (15.91 to 18.08)  0.47 (0.45 to 0.51) | 26.93 (26.57 to 27.29)  0.93 (0.93 to 0.93) |
| **Transfer within first 48 hours (any direction)**  **aRR (95% CI)** | 9.61  (8.83 to 10.39)  **Ref** | 9.98  (9.17 to 10.78)  1.05 (0.94 to 1.17) | 11.33  (10.49 to 12.16)  1.19 (1.07 to 1.33) | 10.27  (9.47 to 11.08)  1.07 (0.96 to 1.19) | 10.31  (9.50 to 11.12)  1.08 (0.96 to 1.20) | 10.30  (9.46 to 11.13)  1.08 (0.97 to 1.21) | 10.92  (10.06 to 11.78)  1.14 (1.02 to 1.28) | 9.69  (8.83 to 10.55)  1.0 (0.89 to 1.13) | 10.69  (9.80 to 11.57)  1.12 (0.99 to 1.25) | 10.15  (9.28 to 11.02)  1.06 (0.94 to 1.19) | 10.0 (9.14 to 10.87)  1.05 (0.93 to 1.18) | 10.36 (10.12 to 10.60)  1.0 (0.99 to 1.01) |
| **Transfer within first 48 hours (upwards)**  **aRR (95% CI)** | 6.58  (5.92 to 7.24)  **Ref** | 6.67  (6.0 to 7.34)  1.04 (0.90 to 1.19) | 7.53  (6.83 to 8.22)  1.17 (1.02 to 1.34) | 6.55  (5.89 to 7.20)  1.0 (0.87 to 1.16) | 7.09  (6.41 to 7.78)  1.10 (0.95 to 1.26) | 6.66  (5.97 to 7.35)  1.03 (0.89 to 1.19) | 7.62  (6.89 to 8.36)  1.10 (0.96 to 1.27) | 7.20 (6.45 to 7.95)  1.06 (0.91 to 1.22) | 7.34  (6.60 to 8.09)  1.09 (0.95 to 1.26) | 7.37  (6.61 to 8.12)  1.10 (0.96 to 1.28) | 7.66 (6.89 to 8.43)  1.17 (1.02 to 1.35) | 7.14 (6.93 to 7.34)  1.01 (1.0 to 1.02) |
| **Transfer within first 48 hours (horizontal)**  **aRR (95% CI)** | 1.29  (0.99 to 1.59)  **Ref** | 1.56  (1.22 to 1.88)  1.19 (0.87 to 1.64) | 1.76  (1.41 to 2.10)  1.35 (1.0 to 1.83) | 1.83  (1.48 to 2.19)  1.40 (1.04 to 1.90) | 1.52  (1.20 to 1.85)  1.17 (0.85 to 1.60) | 1.56  (1.22 to 1.90)  1.20 (0.88 to 1.66) | 1.24  (0.94 to 1.55)  0.96 (0.68 to 1.35) | 1.04  (0.75 to 1.34)  0.80 (0.56 to 1.15) | 1.26  (0.94 to 1.57)  0.98 (0.70 to 1.38) | 1.28  (0.96 to 1.61)  1.0 (0.71 to 1.41) | 1.23 (0.91 to 1.55)  0.96 (0.68 to 1.36) | 1.42 (1.32 to 1.51)  0.97 (0.95 to 0.99) |
| **Transfer within first 48 hours (downwards)**  **aRR (95% CI)** | 1.64  (1.31 to 19.84)  **Ref** | 1.61  (1.27 to 1.94)  0.95 (0.71 to1.27) | 2.01  (1.64 to 2.38)  1.19 (0.90 to 1.57) | 1.96  (1.59 to 2.33)  1.17 (0.89 to 1.54) | 1.56  (1.23 to 1.89)  0.93 (0.69 to 1.25) | 1.70  (1.34 to 2.06)  1.02 (0.76 to 1.37) | 1.52  (1.18 to 1.86)  0.90 (0.66 to 1.22) | 1.31  (0.98 to 1.64)  0.80 (0.58 to 1.11) | 1.66  (1.29 to 2.03)  1.02 (0.75 to 1.37) | 1.19  (0.88 to 1.51)  0.73 (0.52 to 1.02) | 0.89 (0.62 to 1.15)  0.54 (0.37 to 0.78) | 1.59 (1.49 to 1.69)  0.96 (0.94 to 0.98) |
| **Any intubated respiratory support**  **aRR (95% CI)** | 54.37 (53.04 to 55.70)  **Ref** | 52.58 (51.24 to 53.92)  0.98 (0.95 to 1.0) | 52.22 (50.91 to 53.55)  0.97 (0.95 to 1.0) | 52.18 (50.85 to 53.51)  0.96 (0.93 to 0.99) | 51.23 (49.89 to 52.56)  0.94 (0.92 to 0.97) | 49.77 (48.39 to 51.15)  0.92 (0.90 to 0.95) | 45.96 (44.58 to 47.34)  0.85 (0.82 to 0.87) | 44.92 (43.47 to 46.36)  0.82 (0.79 to 0.84) | 43.33 (41.91 to 44.76)  0.80 (0.78 to 0.83) | 38.86 (37.45 to 40.28)  0.72 (0.70 to 0.75) | 39.04 (37.63 to 40.45)  0.72 (0.70 to 0.75) | 47.34 (46.94 to 47.74)  0.96 (0.96 to 0.97) |
| **PDA closure by ligation or device**  **aRR (95% CI)** | 0.28 (0.14 to 0.42)  **Ref** | 0.17 (0.06 to 0.28)  0.61 (0.27 to 1.40) | 0.16 (0.06 to 0.27)  0.59 (0.26 to 1.34) | 0.33 (0.18 to 0.49)  1.17 (0.59 to 2.32) | 0.26 (0.12 to 0.39)  0.89 (0.43 to 1.85) | 0.12 (0.02 to 0.21)  0.43 (0.17 to 1.11) | 0.08 (0.0 to 0.16)  0.28 (0.09 to 0.83) | 0.11  (0.01 to 0.21)  0.38 (0.14 to 1.05) | 0.15 (0.04 to 0.26)  0.55 (0.22 to 0.1.35) | 0.0  (0.0 to 0.0)  0.0 (0.0 to 0.0) | 0.04 (0 to 0.10  0.16 (0.04 to 0.69) | 0.15 (0.12 to 0.18)  0.86 (0.81 to 0.92) |
| **Receiving any own mother’s milk at discharge**  **aRR (95% CI)** | 59.43 (58.10 to 60.78)  **Ref** | 58.31 (56.96 to 59.67)  0.98 (0.95 to 1.01) | 58.84 (57.51 to 60.17)  0.99 (0.96 to 1.01) | 59.36 (58.02 to 60.70)  1.0 (0.97 to 1.02) | 58.30 (56.96 to 59.63)  0.98 (0.96 to 1.01) | 59.06 (57.67 to 60.44)  0.99 (0.97 to 1.02) | 62.04 (60.68 to 63.41)  1.04 (1.02 to 1.07) | 64.61 (63.18 to 66.04)  1.09 (1.06 to 1.12) | 62.53 (61.11 to 63.94)  1.05 (1.02 to 1.08) | 63.46 (62.04 to 64.88)  1.07 (1.04 to 1.09) | 64.41 (63.0 to 65.82)  1.08 (1.06 to 1.11) | 61.09 (60.68 to 61.50)  1.01 (1.0 to 1.01) |

**The “Overall trend” column represents the adjusted relative risk (aRR) for each one-year increase in birth year, derived from modified Poisson regression models adjusted for baseline covariates.**

**Supplemental Figure legends**

Figure S1

The proportion of Extremely Preterm and Very Preterm admissions transferred within the first 48 hours, 2013 to 2023.

Upwards transfer: Infant transferred from a neonatal unit to another unit providing a higher designated level of care;

Downwards transfer: Infant transferred from a neonatal unit to another unit providing a lower designated level of care;

Horizontal transfer: Infant transferred between neonatal units of the same designated level of care.
