# Supplementary material for: Changes in admissions, care processes and outcomes for very and extremely preterm infants in England and Wales: an 11-year whole population study
Source: BMJ Public Health. 2026 Mar 31;4(1):e004256. doi: 10.1136/bmjph-2025-004256 (PMC13052741; doi:10.1136/bmjph-2025-004256)
Supplement: online supplemental file 1 [file bmjph-4-1-s003.docx]

**Supplementary Analysis Code**

This Supplement provides fully annotated Stata (version 18) code used to conduct all statistical analyses, sensitivity analyses, model diagnostics, and figure generation reported in the main manuscript and Supplementary Tables and Figures. The code is structured to mirror the analytic workflow of the study, including missing-data assessment and complete-case comparisons, population-level admission-rate analyses using offset-based Poisson regression, individual-level modified Poisson regression for temporal trends in care processes and clinical outcomes, sensitivity analyses using negative binomial models, formal assessment of model fit and dispersion, and construction of smoothed trend plots with pointwise 95% confidence intervals. Variable names correspond to those used in the National Neonatal Research Database extract analysed for this study. All analyses are fully reproducible subject to data-access permissions.

/********************************************************************

Supplementary Analysis Code

Title: Changes in admissions, care processes and clinical outcomes

for very and extremely preterm infants in England and Wales

Data source: National Neonatal Research Database (NNRD)

Software: Stata 18

Purpose: Reproducible code supporting all analyses reported in the

main manuscript and Supplementary Materials

********************************************************************/

version 18

clear all

set more off

/********************************************************************

SECTION S1. Missing data assessment and complete-case comparison

Purpose:

- Define complete-case indicator using baseline covariates only

- Compare baseline characteristics between complete and incomplete

cases to assess potential selection bias

Corresponds to:

Statistical Analysis – Missing data

********************************************************************/

* Missing maternal ethnicity by birth year and gestational age group

* ga_cat == 1 : Extremely preterm

* ga_cat == 2 : Very preterm

foreach ga in 1 2 {

di "--------------------------------------------------"

di "Gestational age group (ga_cat) = `ga'"

di "--------------------------------------------------"

forvalues y = 2013/2023 {

di "Birth year = `y'"

codebook MumEthnicityReduced_2 if BirthYear == `y' & ga_cat == `ga'

}

}

* Define complete-case indicator using baseline covariates only

gen complete = !missing(MumEthnicityReduced_2, GestationWeeks, BW_UKWHO, ///

Gender, Multiplicity, SteroidsAntenatal)

label define comp 0 "Missing ≥1 baseline variable" 1 "Complete case"

label values complete comp

* Compare baseline characteristics by completeness

tab complete

tab Gender complete, col

tab Multiplicity complete, col

tab SteroidsAntenatal complete, col

tabstat GestationWeeks, by(complete) stat(n mean sd)

tabstat BW_UKWHO, by(complete) stat(n mean sd)

/********************************************************************

SECTION S2. Population-level admission rate analyses

Purpose:

- Estimate annual admission rates for EPT and VPT infants

- Fit Poisson regression models with log-offset for live births

- Model birth year as a continuous variable to estimate

average annual relative change

Notes:

- Offset-based models are used only for population-level counts

Corresponds to:

Statistical Analysis – Annual admission rates

********************************************************************/

* Annual admission rates using Poisson regression with live-birth offset

glm counts birthyear if ga_cat == 1, family(poisson) link(log) ///

exposure(livebirth) eform vfactor(1.385)

glm counts birthyear if ga_cat == 2, family(poisson) link(log) ///

exposure(livebirth) eform vfactor(1.407)

* Ethnicity-specific admission rates using ethnicity-specific denominators

glm Asian birthyear if ga_cat==1, family(poisson) link(log) exposure(AsianLB) eform vfactor(1.238)

glm Asian birthyear if ga_cat==2, family(poisson) link(log) exposure(AsianLB) eform vfactor(1.238)

glm Black birthyear if ga_cat==1, family(poisson) link(log) exposure(BlackLB) eform vfactor(1.10)

glm Black birthyear if ga_cat==2, family(poisson) link(log) exposure(BlackLB) eform vfactor(1.248)

glm Mixed birthyear if ga_cat==1, family(poisson) link(log) exposure(MixedLB) eform vfactor(1.10)

glm Mixed birthyear if ga_cat==2, family(poisson) link(log) exposure(MixedLB) eform vfactor(1.125)

glm Other birthyear if ga_cat==1, family(poisson) link(log) exposure(OtherLB) eform vfactor(1.08)

glm Other birthyear if ga_cat==2, family(poisson) link(log) exposure(OtherLB) eform vfactor(1.14)

glm White birthyear if ga_cat==1, family(poisson) link(log) exposure(WhiteLB) eform vfactor(1.342)

glm White birthyear if ga_cat==2, family(poisson) link(log) exposure(WhiteLB) eform vfactor(1.366)

* admission rate by year and mother's ethnicity

* denominator the total live birth

glm admission year if ga_group==0 & ethnicity==0, family(poisson) link(log) exposure(livebirth) eform vfactor(1.238)

glm admission year if ga_group==1 & ethnicity==0, family(poisson) link(log) exposure(livebirth) eform vfactor(1.238)

glm admission year if ga_group==0 & ethnicity==1, family(poisson) link(log) exposure(livebirth) eform vfactor(1.10)

glm admission year if ga_group==1 & ethnicity==1, family(poisson) link(log) exposure(livebirth) eform vfactor(1.248)

glm admission year if ga_group==0 & ethnicity==2, family(poisson) link(log) exposure(livebirth) eform vfactor(1.10)

glm admission year if ga_group==1 & ethnicity==2, family(poisson) link(log) exposure(livebirth) eform vfactor(1.125)

glm admission year if ga_group==0 & ethnicity==3, family(poisson) link(log) exposure(livebirth) eform vfactor(1.08)

glm admission year if ga_group==1 & ethnicity==3, family(poisson) link(log) exposure(livebirth) eform vfactor(1.14)

glm admission year if ga_group==0 & ethnicity==4, family(poisson) link(log) exposure(livebirth) eform vfactor(1.342)

glm admission year if ga_group==1 & ethnicity==4, family(poisson) link(log) exposure(livebirth) eform vfactor(1.366)

/********************************************************************

SECTION S3. Individual-level regression analyses (primary models)

Purpose:

- Estimate temporal trends in care processes and clinical outcomes

- Use modified Poisson regression with robust variance estimation

- Adjust for baseline covariates

Notes:

- Birth year is modelled as a continuous variable

- No population offset is used in these models

Corresponds to:

Statistical Analysis – Temporal trends in outcomes

********************************************************************/

* Modified Poisson regression with robust variance

foreach g in 1 2 {

glm Clinical_outcome_var i.Gender GestationWeeks i.Multiplicity ///

BW_UKWHO BirthYear if ga_cat == `g', ///

family(poisson) link(log) vce(robust) eform

glm Care_process_var i.Gender GestationWeeks i.Multiplicity ///

BW_UKWHO BirthYear if ga_cat == `g', ///

family(poisson) link(log) vce(robust) eform

}

* Ethnicity-stratified models

foreach g in 1 2 {

bys MumEthnicityReduced_2: ///

glm Clinical_outcome_var i.Gender GestationWeeks i.Multiplicity ///

BW_UKWHO BirthYear if ga_cat == `g' & MumEthnicityReduced_2 < ., ///

family(poisson) link(log) vce(robust) eform

bys MumEthnicityReduced_2: ///

glm Care_process_var i.Gender GestationWeeks i.Multiplicity ///

BW_UKWHO BirthYear if ga_cat == `g' & MumEthnicityReduced_2 < ., ///

family(poisson) link(log) vce(robust) eform

}

/********************************************************************

SECTION S4. Sensitivity analyses

Purpose:

- Assess robustness of findings to alternative model assumptions

- Fit negative binomial regression models allowing for

extra-Poisson variation

- Repeat analyses under alternative specifications

Corresponds to:

Statistical Analysis – Sensitivity analyses

********************************************************************/

* Negative binomial models for admission counts

nbreg counts BirthYear if ga_cat == 1, exposure(livebirth) vce(robust) irr

nbreg counts BirthYear if ga_cat == 2, exposure(livebirth) vce(robust) irr

* Excluding extremely preterm infants <24 weeks

glm Clinical_outcome_var i.Gender GestationWeeks i.Multiplicity ///

BW_UKWHO BirthYear if ga_cat == 1 & GestationWeeks >= 24, ///

family(poisson) link(log) vce(robust) eform

glm Care_process_var i.Gender GestationWeeks i.Multiplicity ///

BW_UKWHO BirthYear if ga_cat == 1 & GestationWeeks >= 24, ///

family(poisson) link(log) vce(robust) eform

bys MumEthnicityReduced_2: glm SteroidsAntenatal i.Gender GestationWeeks i.Multiplicity///

BW_UKWHO BirthYear if GestationWeeks>=24 & ga_cat==1 & MumEthnicityReduced_2!=., ///

family(poisson) link(log) vce(robust) eform

bys MumEthnicityReduced_2: glm Level3Birth i.Gender GestationWeeks i.Multiplicity ///

BW_UKWHO BirthYear if GestationWeeks>=24 & ga_cat==1 & MumEthnicityReduced_2!=., ///

family(poisson) link(log) vce(robust) eform

* Negative binomial sensitivity for individual-level outcomes

foreach g in 1 2 {

nbreg Clinical_outcome_var i.Gender GestationWeeks i.Multiplicity ///

BW_UKWHO BirthYear if ga_cat == `g', vce(robust) irr

nbreg Care_process_var i.Gender GestationWeeks i.Multiplicity ///

BW_UKWHO BirthYear if ga_cat == `g', vce(robust) irr

}

/********************************************************************

SECTION S5. Model diagnostics and goodness-of-fit

Purpose:

- Assess Poisson model assumptions

- Evaluate dispersion using Pearson chi-squared / df

- Extract model-fit statistics reported in the Supplement

Dispersion was assessed using the Pearson chi-squared statistic divided by the degrees of freedom from Poisson models fitted without robust variance estimation; values close to 1 indicate adequate fit.

Corresponds to:

Statistical Analysis – Model adequacy

********************************************************************/

* NOTE: Replace OUTCOME_VAR with the relevant outcome variable

glm OUTCOME_VAR i.Gender GestationWeeks i.Multiplicity ///

BW_UKWHO BirthYear, family(poisson) link(log) eform

scalar phi = e(deviance_p) / e(df)

display "Pearson chi-squared / df = " phi

display "sqrt(Pearson chi-squared / df) = " sqrt(phi)

* AIC and Pearson chi-squared / df are also obtained directly

* from the Poisson regression output

/********************************************************************

SECTION S6. Figures and graphical presentation

Purpose:

- Generate smoothed temporal trend plots

- Add pointwise 95% confidence intervals

- Produce figures shown in the main manuscript and Supplement

Corresponds to:

Figures 1–X and Supplementary Figures

********************************************************************/

* Smoothed monthly admission trends with 95% CIs

* NOTE: Replace OUTCOME_VAR with the relevant outcome variable

twoway ///

lpolyci OUTCOME_VAR time_months if ga_cat == 1, kernel(gaussian) bwidth(0.5) level(95) ///

|| scatter counts time_months if ga_cat == 1, msymbol(o) ///

|| lpolyci counts time_months if ga_cat == 2, kernel(gaussian) bwidth(0.5) level(95) ///

|| scatter counts time_months if ga_cat == 2, msymbol(o) ///

ytitle("Number of monthly admissions N") ///

xtitle("Year") ///

xlabel(2013(1)2024) ylabel(50(50)600) ///

legend(size(tiny)) ///

legend(order(3 "{bf:Extremely preterm admissions; Observed}" ///

2 "{bf:Fitted regression line}" 1 "{bf:95% Confidence interval}" ///

5 "{bf:Very preterm admissions; Observed}" 4 "{bf:Fitted regression line}")) ///

legend(position(0) bplacement(neast) region(lstyle(solid)))

* Offset-based Poisson model for ethnicity-specific admission rates

poisson admission i.ga_group##i.ethnicity##i.year, exposure(livebirth)

margins ga_group#ethnicity#year, predict(ir)

twoway ///

rcap _ci_lb _ci_ub year if ethnicity == 0 || ///

line admrate year if ethnicity == 0 || ///

rcap _ci_lb _ci_ub year if ethnicity == 1 || ///

line admrate year if ethnicity == 1 || ///

rcap _ci_lb _ci_ub year if ethnicity == 2 || ///

line admrate year if ethnicity == 2 || ///

rcap _ci_lb _ci_ub year if ethnicity == 3 || ///

line admrate year if ethnicity == 3 || ///

rcap _ci_lb _ci_ub year if ethnicity == 4 || ///

line admrate year if ethnicity == 4, ///

by(ga_group) ///

ytitle("Admission rate by maternal ethnicity (%)") ///

xtitle("Year") ///

xlabel(2013(2)2024) ///

ylabel(0(0.25)1.25) ///

legend(size(tiny))
